# The role of climate change and urban development on compound dry-hot extremes across US cities

**DOI:** 10.1038/s41467-023-39205-x

**Published:** 2023-06-14

**Authors:** Mahshid Ghanbari, Mazdak Arabi, Matei Georgescu, Ashley M. Broadbent

**Affiliations:** 1 Civil and Environmental Engineering Department, Colorado State University, Fort Collins, CO USA; 2 School of Geographical Sciences and Urban Planning, Arizona State University, Tempe, AZ USA; 3 Urban Climate Research Center, Arizona State University, Tempe, AZ USA; 4 National Institute of Weather and Atmospheric Research, Wellington, New Zealand

**Keywords:** Natural hazards, Projection and prediction

## Abstract

Compound dry-hot extreme (CDHE) events pose greater risks to the environment, society, and human health than their univariate counterparts. Here, we project decadal-length changes in the frequency and duration of CDHE events for major U.S. cities during the 21st century. Using the Weather Research and Forecasting (WRF) model coupled to an urban canopy parameterization, we find a considerable increase in the frequency and duration of future CDHE events across all U.S. major cities under the compound effect of high-intensity GHG- and urban development-induced warming. Our results indicate that while GHG-induced warming is the primary driver of the increased frequency and duration of CDHE events, urban development amplifies this effect and should not be neglected. Furthermore, We show that the highest frequency amplification of major CDHE events is expected for U.S. cities across the Great Plains South, Southwest, and the southern part of the Northwest National Climate Assessment regions.

## Abstract (web summary)

This study projects a significant increase in the frequency and duration of compound dry-hot extreme (CDHE) events in major U.S. cities during the 21st century. GHG-induced warming is the main driver, amplified by urban development.

## Introduction

Extreme hot or dry conditions (i.e., low precipitation) can impair ecosystems and threaten human wellbeing^1,2^. Due to a strong negative correlation between temperature and precipitation, dry conditions usually coincide with hot extremes during warm seasons^3–5^. The concurrent occurrence of hot and dry events, which is considered a compound event^6,7^, can exacerbate the harmful effects of hot and dry conditions on human health, environment, and economies^7–9^. Contiguous hot days without precipitation can cause increased demand for water and energy resources^10^, as well as promote and intensify the formation of unfavorable urban climates by increasing allergens and particulate matter concentration that could be a serious threat to urban dwellers’ health^11^. Among the adverse ecosystem consequences of prolonged hot and dry episodes are the increasing frequency and intensity of wildfires and significant plant loss^9,12–14^.

Global greenhouse gas (GHG) emissions as well as land use and land cover change (LULCC) resulting from urbanization can alter precipitation and temperature patterns, leading to more frequent and severe heat extremes and droughts^15–17^. In recent years, the world has witnessed several compound dry-hot extreme (CDHE) events in different locations including the United States^18,19^, Europe^20^, China^21^, Russia^22^, Australia^23,24^, and Brazil^14,25^. Additionally, the increased occurrence of CDHEs in observational records has been reported by several studies^26–30^. These observations call for an improved understanding of the future characteristics and variations of CDHE events in order to enable effective adaptation to future changes in the characteristics of these extreme events.

Under rapidly changing environmental conditions, rigorous characterization of nonstationary CDHE events is predicated upon the incorporation of not only observational data over historical conditions but also model-driven climate simulations for future conditions. The use of global climate models (GCMs) has enabled considerable research focused on characterizing future CDHE events at the country^31–33^, continent^34,35^, and global^4,36,37^ scales. The findings, however, are dependent on the accuracy and resolution of climate projections, the definition of CDHE events as well as specific event characteristics that have been investigated. For example, Zscheischler and Seneviratne (2017) show that in many regions across the globe, under the high-intensity GHG concentration scenario, the frequency of hot and dry warm seasons could increase by a factor of 10 between the period 1870–1969 and 2001–2100. More recently, Bevacqua et al. (2022) demonstrated that with a 2 °C increase in global average temperature compared to preindustrial conditions (in line with the Paris Agreement), the frequency of CDHE events would increase fourfold compared to a historical period centered on 1950–1980. In addition, a robust increase in the frequency of summertime CDHE events between historically observed and future periods are reported over most parts of China^32^ and central Europe^35^. Virtually all of these studies have focused on assessing the frequency of future CDHE events due to independent impacts of GHG-induced warming.

However, the present work differs from past research in two main respects. First, this study provides the analysis of projected changes in frequency and duration of CDHE events across the US with consideration of the compound effect of GHG- and urban development-induced warming. Urban development-induced LULCC has had and is expected to continue having impacts on local to regional warming, a phenomenon referred to as the urban heat island (UHI), by virtue of replacing natural landscapes with roads, buildings, and an enhancement in anthropogenic heat release^38–40^. As a result, the absorption and storage of solar radiation are enhanced within the built environment, and evaporative cooling is reduced. Studies show that GHG and urban development-induced impacts can be of comparable magnitudes over cities^16,38,41^. Despite this evidence, the impacts of urban development-induced warming have been largely ignored in previous efforts to evaluate the characteristics of future CDHE events. Omission of urban development-induced effects may lead to inaccurate estimation of the regional-scale characteristics of future CDHE events. Here we account for the effects of GHG emissions and urban development on CDHE events, both individually and in combination, across ecohydrological regions undergoing disparate climate and urban development trajectories. As such, our study decomposes the impacts of urban development and GHG emissions on future CDHE events and analyses the potential for both processes to cause a synergistic amplification of future CDHE events.

Second, this study differs from previous research in the field by specifically focusing on persistent short-term dry spell events that typically last several days to several weeks, rather than long-term drought events that develop slowly over months or even years. Previous studies focusing on CDHE events have predominantly defined drought at a relatively coarse temporal scale, such as monthly, seasonal, or yearly, without precisely considering their duration^4,19,26,35,42–45^. In contrast, our study defines dry events as contiguous days with low precipitation, enabling us to investigate the change in duration of these short-term dry spell events. Unlike long-term drought events, short-term dry spell events develop rapidly and intensively^46,47^, and their duration is a critical factor in determining their potential impacts on the environment and society. Whether these events last for a few days or extend over a more prolonged period can have direct implications for the severity and scope of subsequent impacts on human health and environment.

Here, we use a suite of 10-year regional climate model simulations to investigate the effect of GHG- and urban development-induced warming on the frequency and duration of CDHE events across 50 major United States cities in varying ecohydrological regions during the end-of-century (2090–2099) relative to the start-of-century (2000–2009) period. Climate projections are conducted with the Advanced Research version (v 3.6) of the Weather Research and Forecasting (hereafter WRF) regional climate model dynamically coupled to a single-layer urban canopy model^38^. We establish an extended capability of WRF, by characterizing the joint probability of high temperature and low precipitation events for major US cities. We define a dry event as consecutive days with daily total precipitation of less than one millimeter and a hot event as periods of consecutive days with the daily maximum temperature above the 90th percentile of the daily maximum of the baseline period during the extended summer (May–October, Figure S1) as the highest heat-related stresses occur during this time of year in the United States^48,49^. Subsequently, CDHE events are identified based on the concurrence of hot and dry events^27,37,50^. In this fashion, we investigate the response of CDHE events, in terms of changes in frequency and duration, to urban development- and GHG-induced warming across 50 major US cities and highlight regions that are projected to undergo amplification of CDHE events. Our results show that under high-intensity GHG emissions and urban development, the frequency and duration of CDHE events would increase across all cities. However, the magnitude of projected change varies regionally. The increased frequency and duration are mainly driven by GHG-induced warming; however, urban development amplifies the effect of GHG-induced warming and should not be ignored in the characterization of future CDHE events.

## Results

### Historical changes in the occurrence of CDHE days (1950–2020)

**Fig. 1:**
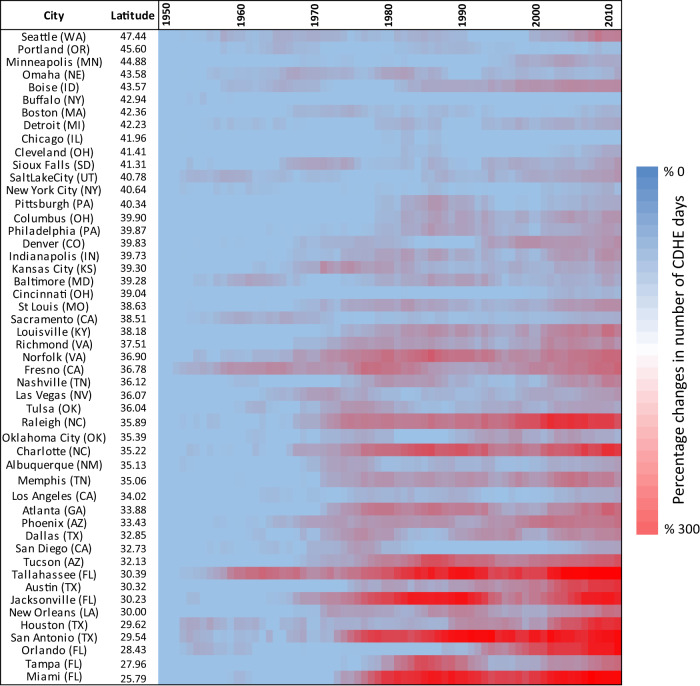
Historical percentage changes in the average annual number of compound dry-hot extreme (CDHE) days per extended summer. The climate data range from 1950 to 2020, analyzed using a 10-year overlapping window (e.g., 1950 indicates data from 1950 to 1959). Source data are provided as a Source Data file.

According to the IPCC’s Sixth Assessment Report’s Summary for Policymakers^6^, climate change due to human influence has likely increased the global frequency of concurrent heatwaves and droughts since the 1950s. We first provide observational evidence indicating the change in the number of CDHE days during the period 1950 to 2020 at the scale of individual cities. Observed daily maximum precipitation and temperature data from NOAA’s National Climatic Data Center (NCDC^51^) are used to evaluate changes in the average annual number of CDHE days per extended summer (hereafter ${\bar{f}}_{{CDHE}}$) from 1950 to 2020 based on a 10-year overlapping window (i.e., 1950 indicates data from 1950 to 1959). Fig. 1 displays the percent change in the ${\bar{f}}_{{CDHE}}$ in 50 major cities of the United States, sorted vertically according to their latitudes. Here, the percent change is defined as the difference in the number of CDHE days in each window relative to the window of 1950–1959, divided by the total number of CDHE days. An increasing trend in the ${\bar{f}}_{{CDHE}}$ is found in 48 out of the 50 cities (the lone exceptions being Buffalo and Chicago). The largest change is found across sunbelt cities, which have undergone a 100 to 300 percent increase in ${\bar{f}}_{{CDHE}}$ in 2011–2020 relative to 1950–1959. Among cities at higher latitudes, northwestern cities (e.g., Seattle and Boise) have experienced a noticeable increase in the number of CDHE events, especially during the last two decades. Given the historical evidence demonstrating a considerable increase in compound hot and dry conditions, it is imperative that we examine future changes.

### Future characteristics of CDHE events

We use WRF simulation results to characterize CDHE events over future climatic and urban landscape conditions. Previous research has found satisfactory model performance based on comparisons of the contemporary WRF simulation against 10 years of observed temperature^38^ and precipitation^15^. Here, the performance validity of the model is established for simulation of the joint probability of high temperature and low precipitation events. We achieve this by testing the hypothesis that the distributions of CHDE events based on observed and simulation data are from the same statistical distribution using the two-sample Kolmogorov–Smirnov (KS) test. The null hypothesis is rejected when the *p* value of the test is less than the significance level of 0.05. The closer the *p* value is to 1, the better the fit between the observed data and model simulations. The KS test shows that the probabilistic characteristics of the observed data and contemporary WRF simulation of CDHE events are statistically the same in all 50 cities, with the *p* value ranging from ~0.2 to 1, with almost two-thirds of cities having *p* values >0.8 (Figure S2). Visual comparison between cumulative distribution functions (CDF) of observed and simulated CDHE events, for each city independently, are provided in Supplementary information (Top panel in Figures S3 to S52). The goodness-of-fit of the simulated CDHE events with observed data indicates that the decadal-length contemporary WRF simulation accurately reproduces both the frequency as well as duration of CDHE events across US cities. We use WRF end-of-century simulation results to project compound CDHE events under a future climate condition characterized by increased emissions of GHGs and urban development.

**Fig. 2:**
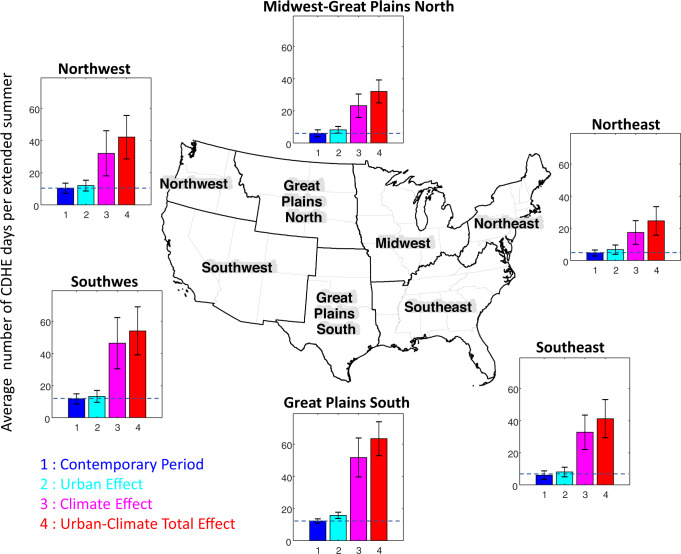
The average number of compound dry-hot extreme (CDHE) days per extended summer (${\bar{{{{{{\rm{f}}}}}}}}_{{{{{{\rm{CDHE}}}}}}}$) over the future period of 2090–2099 relative to the contemporary period of 2000–2009. Dark blue represents the contemporary period, while light blue, pink, and red, respectively represent the future period under the urban effect, climate effect, and urban-climate total effect. Source data are provided as a Source Data file.

We use US National Climate Assessment (NCA) regions to group cities with similar climatological conditions. The results reveal a significant increase in the ${\bar{f}}_{{CDHE}}$, during 2090–2099 relative to 2000–2009 in all NCA regions (Fig. 2). During 2000–2009, ${\bar{f}}_{{CDHE}}$ ranges from 4.7 days for cities located in the Northeast to 12.3 days for cities across the Great Plains South region (Fig. 2, the first [blue] bar in each panel). This baseline CDHE event duration is represented by a dashed blue line against which future impacts can be compared. The value of each bar minus the value of the dashed blue line illustrates changes in ${\bar{f}}_{{CDHE}}$ relative to the baseline ${\bar{f}}_{{CDHE}}$ in response to urban development-induced warming (hereafter urban effect), GHG-induced climate change (hereafter climate effect), and the total effect of urban development-induced warming, and GHG-induced climate change (hereafter urban-climate total effect). The urban effect and climate effect represent the direct effects of increasing urban development or GHG concentrations from 2010 to 2100 levels, respectively (e.g., urban development held constant for climate effect). The urban-climate total effect represents the simultaneous effects of increasing urban development and GHG concentrations from 2010 to 2100 levels.

The climate effect (Fig. 2, the third [magenta] bar) would significantly increase ${\bar{f}}_{{CDH}E}$ in all NCA regions. The largest increase is projected in cities within the Great Plain South (additional 39.5 CDHE days) followed by the Southwest (additional 34.6 CDHE days), Southeast (additional 26.6 CDHE days), and Northwest (additional 21.6 CDHE days) regions. In comparison, the urban effect is likely to cause a considerably less increase (up to additional 3.5 CDHE days) in ${\bar{f}}_{{CDHE}}$ in all regions (Fig. 2, the second [light blue] bar). The additional CDHE days due to the urban-climate total effect (Fig. 2, the fourth [red] bar) reveal that climate and urban effect do not add up linearly; instead, they interact nonlinearly to increase projected ${\bar{f}}_{{CDHE}}$. In other words, the interaction of the urban and climate effect has a positive impact on the increase in ${\bar{f}}_{{CDHE}}$ in all NCA regions. For instance, while the value of ${\bar{f}}_{{CDHE}}$ is projected to increase in cities within the Great Plains South from 12.2 to 63.4 days due to the urban-climate total effect, the projected value ${\bar{f}}_{{CDHE}}$ based on the linear summation of the urban and climate effect is 55.2 days. Also, in the Southwest region ${\bar{f}}_{{CDHE}}$ is projected to increase from 11.7 to 54 days to the urban-climate total effect (additional 42.3 days). However, linear summation of the urban and climate effect lead to an additional 36.1 days. We further explore the changes in the characterization of future CDHE events in terms of frequency and duration.

**Fig. 3:**
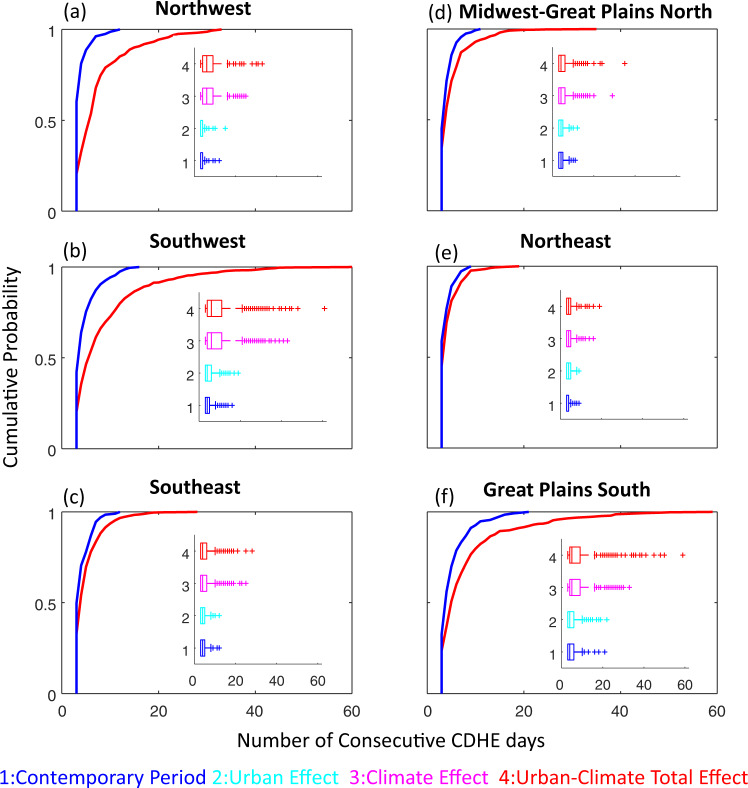
The empirical cumulative distribution functions (CDF) and boxplots of compound dry-hot extreme (CDHE) events over the future period of 2090–2099 relative to the contemporary period of 2000–2009. **a** Northwest, **b** Southwest, **c** Southeast, **d** Midwest-Great Plains North, **e** Northeast, **f** Great Plains South National Climate Assessment (NCA) regions. Dark blue represents the contemporary period, while light blue, pink, and red, respectively, represent the future period under the urban effect, climate effect, and urban-climate total effect. The lower and upper lines of the boxes indicate the 25th (Q1) and 75th (Q3) quartiles, whereas the middle line of the box indicates the median value. For each boxplot, the whiskers extend to the most extreme data point which is no >1.5 times the interquartile range. Source data are provided as a Source Data file.

We project that the median duration of CDHE events will increase by the end-of-century in all NCA regions (Fig. 3, inset). Cities located within the Northwest NCA region are projected to undergo the largest increase in the median duration of CDHE events, from an average of 3 days to 6 days. Changes are greater and more noticeable for higher quantiles (i.e., less-frequent events) in all regions. For instance, the 0.95 quantile of CDHE event duration is projected to increase notably due to the urban-climate total effect from 12.3 to 26 days in the Great Plains South, from 11 to 26 days in the Southwest, and from 7 to 22 days in the Northwest NCA regions. Although less noticeable, the 0.95 quantile is also likely to increase in the Southeast from 8 to 11 days, in the Midwest-Great Plains North from 7 to 13 days, and in the Northeast from 7 to 9 days. This increase in quantiles implies that the duration of CDHE events will increase during the 21st century. In particular, less frequent events are likely to become significantly longer in the Great Plains South, Southwest, and Northwest NCA regions.

In terms of increase in event duration, the urban effect independently is small compared to the climate effect in all regions. However, the urban-climate total effect is likely to exacerbate less-frequent longer-duration events compared to the climate effect alone (Fig. 3). Specifically, the 0.95 quantile of the duration of CDHE events due to the urban-climate total effect compared to when only the climate effect is considered, could increase in cities within the Northwest by 2.9 days (from 19.1 to 22 days), the Southwest by 4 days (from 22 to 26 days), and the Great Plains South by 5.1 days (from 23 to 26 days). This finding highlights the importance of the urban effect and its interaction with the climate effect on the increasing duration of CDHE events, particularly for the Northwest, Southwest, and Great Plains South NCA regions. Detailed information for each city is provided in Supplementary information (Middle panel in Figures S3 to S52).

We also show that the probability distributions of CDHE event duration will shift to the right under the urban-climate total effect for all regions (Fig. 3). Such a shift also indicates longer duration events during the 2090–2099 period relative to the 2000–2009 period. The contemporary and future probability distribution functions of event duration are statistically different based on a two-sample KS test (*p* value < 0.05) in all regions. Notably, in the Southwest and Great Plains South NCA regions, the divergence is highly pronounced in the upper tail of the empirical distribution, indicating that especially long-duration events will increase more than short-duration events.

### Frequency and duration amplification of a 20-year CDHE event

**Fig. 4:**
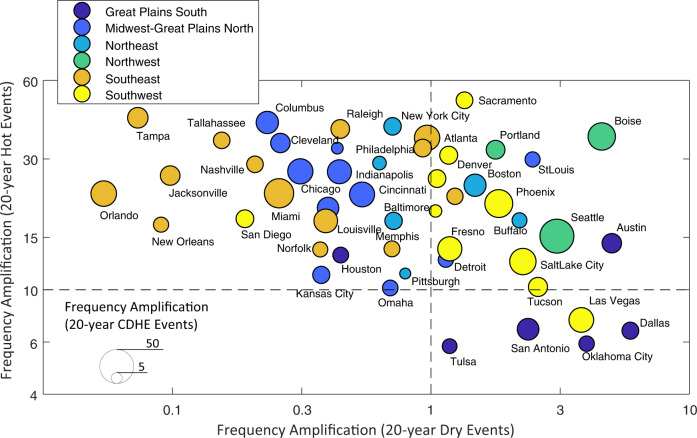
Frequency amplification of 20-year hot, dry, and compound dry-hot extreme (CDHE) events over the future period of 2090–2099 relative to the contemporary period of 2000–2009 under the urban-climate total effect. The color coding represents different National Climate Assessment (NCA) regions. The *x* axis represents the frequency amplification of dry events, the *y* axis represents the frequency amplification of hot events, and the marker size indicates the frequency amplification of CDHE events. Source data are provided as a Source Data file.

We model the probability distribution of the duration of CDHE events for each city using the Generalized Pareto Distribution (GPD) to estimate the return period associated with a given duration (See Materials and Methods, also the bottom panel in Figures S3 to S52). We define frequency amplification as the ratio of the contemporary return period of a given event (the 2000–2009 period) to the future return period of that event (the 2090–2099 period). Frequency amplification is computed for 20-year hot, 20-year dry, and 20-year CDHE events for the last decade of the 21st century under the urban-climate total effect relative to the contemporary period (Fig. 4). The results show that although the frequency of a 20-year hot event is expected to increase in all cities, a 20-year dry event is expected to become less frequent in most cities located in the Southeast, Midwest-Great Plains North NCA region, and have no significant change in some cities within the Northeast and Southwest NCA regions, implying domination of the warming trend as a driver for the increased frequency amplification of CDHE events^30^ in these cities.

**Fig. 5:**
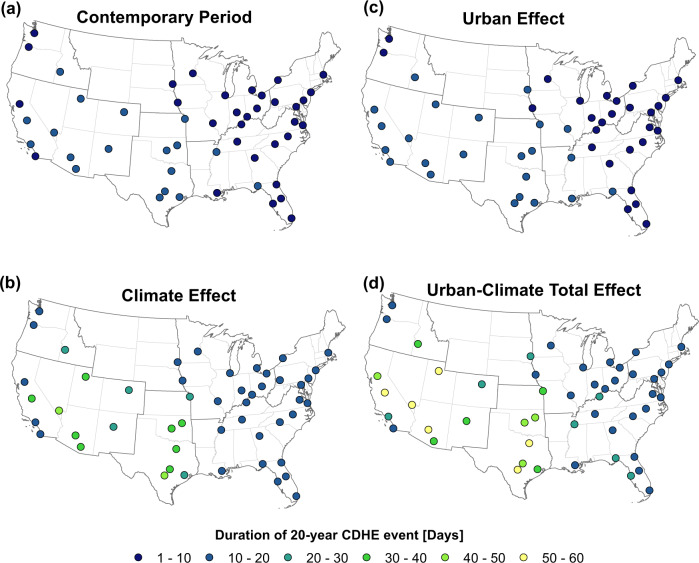
Duration of 20-year compound dry-hot extreme (CDHE) events over the future period of 2090–2099 and the contemporary period of 2000–2009. **a** The contemporary period, **b** the future period under the climate effect, **c** the future period under the urban effect, and **d** the future period under the urban-climate total effect. Source data are provided as a Source Data file.

We also project that the duration of a 20-year CDHE event will increase by the end-of-century (Fig. 5). During the contemporary period, the duration of 20-year CDHE events in cities within the Great Plains South and Southwest NCA regions range from 10 to 20 days. This duration will increase to 40–60 days by the end of century under the urban-climate total effect with the exception of Denver and southwestern Pacific coastal cities. The duration of 20-year events in cities within Southeast, Northeast, and Midwest-Great Plains north are projected to increase from 1–10 days to 10–20 days. The urban effect on the duration of 20-year events is negligible. However, our results show that the urban effect exacerbates the climate effect by increasing the duration of 20-year events in cities across the Great Plains South and Southwest NCA regions, and Boise located in the southern part of the Northwest regions (Fig. 5c, d).

Although investigating changes in the frequency of 20-year events could provide important insights into frequency amplification of these events, a comparison over all regions is difficult to obtain since there is no exact correspondence between the duration of the events and return periods in all regions^52^. The duration of a 20-year CDHE event across different cities under the contemporary period underlines this consideration (Fig. 5a). While the duration of a 20-year event ranges between 20 to 30 days in southern cities located in the Great Plains South and Southwest region, cities across the Northeast, Midwest-Great Plains north, and the southern part of the Southeast region experience 20-year events with durations of 1 to 10 days. The duration of a CDHE event is an important factor in determining the impacts, and as these findings reveal, the level of impact of a 20-year event is not equal in different regions. Thus, for improved assessment of regional frequency amplification, we investigate the changes in the return period of minor and major CDHE events, defined as events with durations of at least 10 and 30 consecutive CDHE days, respectively, across 50 major US cities by the end-of-century.

### Frequency amplification of minor and major CDHE events

**Fig. 6:**
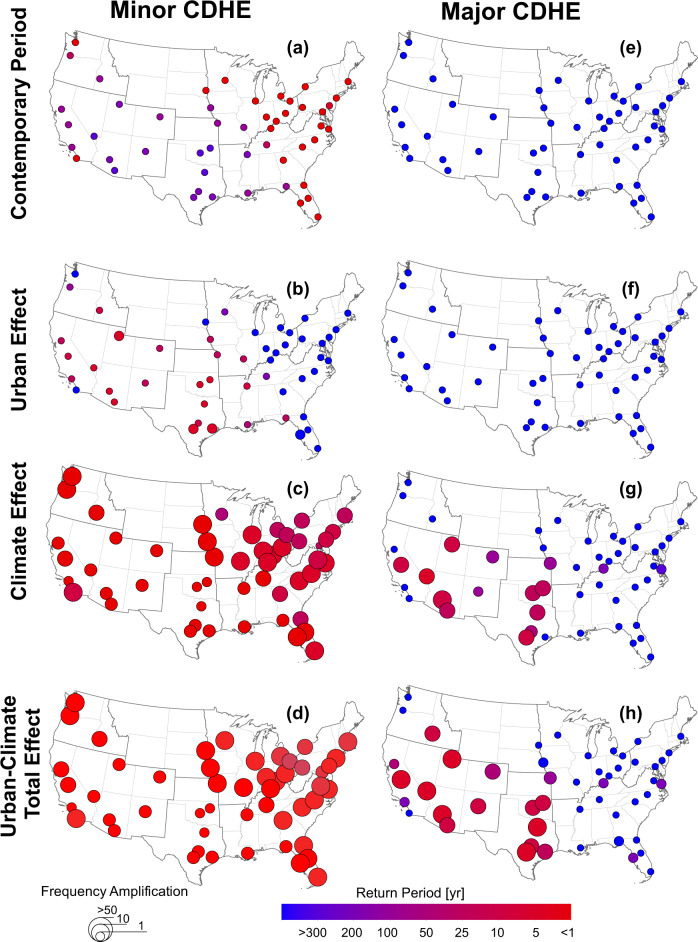
The return period and frequency amplification of minor and major compound dry-hot extreme (CDHE) events over the future period of 2090–2099 and the contemporary period of 2000–2009. **a** minor CDHE events over the contemporary period **b** minor CDHE events over the future period under the urban effect, **c** minor CDHE events over the future period under the climate effect, **d** minor CDHE events over the future period under the urban-climate total effect, **e** major CDHE events over the contemporary period, **f** major CDHE events over the future period under the urban effect, **g** major CDHE events over the future period under the climate effect, and **h** major CDHE events over the future period under the urban-climate total effect. Source data are provided as a Source Data file.

During the contemporary period of 2000–2009, minor CDHE events (i.e., duration >10 consecutive days) occurred with return periods ranging from <1–25 years in cities located across the Great Plains south and Southwest NCA regions (Fig. 6a). Minor contemporary CDHE events are less frequent in cities in other NCA regions with return periods ranging from 100 to 200 years. It should be noted that a T-year return period does not mean that only one event should occur every T-years, but rather that the probability of the T-year event being exceeded is 1/T in every year. By the end-of-century due to the urban-climate total effect (Fig. 6d), all regions are projected to experience minor CHDE events with return periods >10 years. The highest amplification in the frequency of minor events (10–40 times) is projected for cities within the Northeast, Midwest, and southern Southeast NCA regions.

Major CDHE events with a duration greater than 30 consecutive days occurred with return periods greater than 300 years in all cities (Fig. 6e) meaning that these prolonged CDHE events were unlikely to occur during the contemporary period. However, under the urban-climate total effect, these events will become more common in the Great Plains South, Southwest, and the Southern Northwest NCA region: the return period of major events is projected to decrease from over 300 years during 2000–2009 to >25 years during 2090–2099 in these NCA regions, with the lone exception being southern Pacific coastal cities (Fig. 6h). The urban effect, independently, does not alter the frequency of major events. However, the urban effect combines to worsen the climate effect by increasing the frequency of major events in cities across the Great Plains South and Southwest regions, as well as Boise located in the southern Northwest region. The relatively small effect of urban development can be attributed to the fact that urban-induced warming mostly increases evening and nighttime temperatures^38^. Since in this study, daily maximum temperature is used to define CDHE days, the effect of urban development is less pronounced. Although less impactful compared to the climate effect, our results show that ignoring the urban effect leads to underestimation of changes in the frequency and duration of future CDHE events since the interaction between the urban and climate effect does play a meaningful role in increasing the frequency and duration of CDHE events for a number of cities.

The projected changes in the frequency of CDHE events vary regionally. Cities located in the Northeast, Midwest, and Southern Southeast NCA regions are projected to undergo the largest amplification in the frequency of minor events. This finding also holds for coastal cities along the Pacific coasts (e.g., San Diego and Seattle). Previous studies show that these minor (short-term) CDHE events are expected to be amplified more in humid regions^53^. On the other hand, most of the cities located within the Great Plains South, Southwest, and Southern Northwest NCA regions are projected to undergo the largest amplification in the frequency of major CDHE events. Prolonged major CDHE events are projected to occur much more frequently by the end-of-century in regions that currently do not experience major CHDE events (Fig. 6h). These regions may not be adequately prepared for the catastrophic effects of these unprecedented events, and urgent actions are needed to mitigate potential negative impacts. Therefore, it is crucial for policymakers and urban planners to consider regional differences and to tailor mitigation and adaptation strategies to the specific challenges faced by each region.

## Discussion

We present an analysis of changes in frequency and duration of CDHE events during the 21st century in 50 major US cities by accounting for the effect of GHG-induced climate change and urban development individually and in combination. We show the increasing trend in the frequency and duration of CDHE events observed in the United States^29,30,54^. is likely to continue under the effect of GHG- and urban development-induced warming. The impact of urban development has been largely neglected in the characterization of future CDHE events in the United States^31,55^ and across the world^34–37^. Our findings suggest that while GHG-induced warming is the primary driver of the increased frequency and duration of CDHE events, neglecting the impact of urban development-induced warming may lead to an underestimation of changes in the frequency and duration of these events. Therefore, it is crucial to account for the climatic consequences associated with the expansion of the built environment in tandem with GHG emissions to predict future characteristics of CDHE events accurately. We also show that urban development amplifies the effect of GHG-induced warming in all cities although this effect is magnified for cities across the Great Plains South and Southwest NCA regions where ongoing and more extensive urban development is projected. This amplification is due to the replacement of natural vegetation with engineered materials, which enhances urban-induced warming^16,38,39,41^ (as a result of a greater fraction of heat-retaining surfaces and greater flux of waste heat in the urban environment) and exacerbates the heat stress on residents and infrastructure.

Moreover, our findings demonstrate that major CDHE events lasting at least thirty consecutive days are expected to have a higher frequency amplification in cities located in the Great Plains South and Southwest NCA regions^31,54^. Conversely, the amplification of minor CDHE events with at least ten consecutive CDHE days is likely to be higher in cities located within the Northeast, Midwest, and southern part of the Southeast NCA regions^29^. The observed spatial variability in the amplification of CDHE events in different NCA regions can be attributed to several factors, including regional climate, topography, vegetation cover, and proximity to water coastlines. The hot and dry climate conditions during the warm season make non-coastal cities in the Great Plains South and Southwest NCA regions particularly vulnerable to the compounded effects of GHG- and urban development-induced warming. As a result, these regions are likely to experience a more significant increase in the frequency of major CDHE events compared to other regions. On the other hand, cities located in the Northeast, Midwest, and southern part of the Southeast NCA regions, with their more humid and temperate climate, are likely to experience more amplification in minor CDHE events and by the end of the century, the amplification in frequency of major CDHE events in cities located in these regions is unlikely. The observed spatial variability in the amplification of CDHE events in different NCA regions can also be attributed to the replacement of contemporary vegetation with engineered structures, which reduces the cooling effect of vegetation cover (i.e., regulating the surface energy and water balance), and can affect local and regional climate conditions.

The definition of dry conditions used in this study is based on persistent dry spell events, which are continuous days with low precipitation lasting several days to several weeks rather than long-term drought events that develop slowly due to precipitation deficit propagation over months or even years^30^. Although long-term drought events may have more widespread and long-lasting effects, short-term dry spell events have a more immediate impact on the urban environment and human health. The concurrence of these short-term dry spell events with hot conditions promotes and intensifies the formation of unfavorable urban climates, leading to increased allergens and particulate matter concentration. This, in turn, leads to hazardous respiratory health problems for residents^11,56^, particularly those who are vulnerable, such as lower-income individuals lacking access to cooling systems and proper healthcare facilities. During these events, the demand for water, energy, and power intensifies, which can lead to water scarcity and reduce the availability of clean drinking water for residents. The projected increase in frequency and duration of CDHE events could further aggravate these issues in urban areas. While our analysis has focused on urban areas, it is worth noting that rural areas are likely susceptible to similar trends in frequency and duration of CDHE events, albeit with a reduced interaction, and therefore reduced total effect. Given the historical sensitivity of agriculture to episodes of hot and dry conditions, increasing the frequency and duration of major CDHE events can cause substantial negative yield shocks and subsequently increase yield volatility^57,58^. Wildfires, many of which are in close proximity to major urban areas, are likely to become more frequent and problematic as cities expand and encroach upon wildlands^59^. The rural-urban interface (RUI) can play a significant role in driving wildfire occurrence^60^ and the expansion of the RUI due to urbanization can increase the risk of wildfire occurrence by creating more ignition points and making it more difficult to control fires. The expansion of cities can also alter land use and vegetation patterns, which can affect fuel availability and vegetation structure, further increasing the risk of wildfire at the RUI.

By accounting for the effects of both GHG-induced climate change and urban development, our work enhances the understanding of the regional variations in changes in the frequency and duration of CDHE events in the US during the 21st century. The identification of locations where amplification in these events is expected to be greatest can provide policymakers with valuable information for developing appropriate adaptation strategies. Regions most at risk of major CDHE events must plan appropriately to deal with this growing threat by minimizing the heat burden in urban areas while enhancing emergency preparedness plans. Adaptation plans and policies play a critical role to mitigate these impacts in urban areas and improve their resilience to increased CDHE events. Enhanced coordination among scientific communities and local stakeholders is necessary. Solutions will need to be integrative (i.e., cutting across disciplinary boundaries^61^) and will necessitate a holistic characterization of impacts to appropriately examine co-benefits and tradeoffs associated with various adaptation measures aimed at improving urban sustainability. Our findings are an initial step to alert policymakers to the need to respond appropriately to the intensification of CDHE events and suggest appropriate adaptation policies that ameliorate their impacts. Future research should evaluate the effectiveness of heat-mitigating measures in different regions of the country and examine the geographical dependency of these measures^16^ at high spatiotemporal resolution. Such research would provide valuable insights into the most effective strategies to reduce the impact of CDHE events in different regions. Additionally, further investigation is needed to identify vulnerable populations and develop targeted interventions to reduce the adverse health impacts of CDHE events on these groups^62^. We emphasize that we selected the most aggressive climate change (RCP 8.5) and urban development (Integrated Climate and Land-Use Scenarios (ICLUS) A2) projections. Thus, our results are based on the continuity of the present level of CO_2_ emissions and should be interpreted as an upper limit of potential increase in frequency and duration of CDHE events by the end-of-century. In addition, we do not account for GCM uncertainty in evaluating impacts of future climate (only CESM RCP 8.5 is used to define atmospheric boundary conditions).

## Methods

### Climate data and regions

We use the Advanced Research version (v 3.6) of the Weather Research and Forecasting (WRF-ARW) regional climate model which is applied to dynamically downscale the contemporary period (2000–2009) and end-of-century period (2090–2099) urban and regional climate for the contiguous United States^38,39^. The WRF-ARW is a fully compressible non-hydrostatic regional weather forecasting and climate model^63^ which has an extensive history of integration with urban modeling systems^64^. The model domain encompasses the contiguous US at 20 km horizontal grid spacing and contains 310 grid squares in the east-west and 190 grid squares in the north-south directions, respectively. Our simulations represent two decadal periods as bookends for the 21st century, and may not be representative of longer-term natural climatic variability, due to, for example, impacts resulting from the Pacific Decadal Oscillation (PDO) or Atlantic Multidecadal Oscillation (AMO). Indeed, such simulations are warranted but are impractical at the spatiotemporal scale considered (i.e., temporal frequency of three hours and grid spacing of 20 km). Critically, our simulations examine impacts during the summer season, when land-atmosphere interactions dominate local to regional scale climate, and longer-term simulations (e.g., multi-decadal), would not change the overall significance of our results and are therefore not warranted.

Initial and time-dependent boundary conditions for the start-of-century simulation are obtained from the European Centre for Medium-Range Weather Forecasts ‘ERA Interim’ reanalysis^65^. As forcing for end-of-century simulations, the Community Earth System Model Coupled Model Intercomparison Project 5 (CESM CMIP5) RCP 8.5 projection is used^66^. CESM approximates the CMIP5 median in terms of US summertime warming by the end-of-century. RCP 8.5 assumes no explicit climate policy and represents the highest RCP scenario in terms of GHG emissions^67^. Projections of future urban building density are available from the US Environmental Protection Agency Integrated Climate and Land Use Scenarios (ICLUS) dataset^68^. The ICLUS projections are based on the socio-economic storylines from the Special Report on Emissions Scenarios (SRES). Here, we use a single scenario based on the ICLUS A2 projection, which closely mirrors the RCP 8.5 scenario in terms of temporal evolution and radiative forcing magnitude^38^. The scenarios accounted for in our work are based on the most aggressive urban development and GHG-induced warming. To represent urban temperatures (*T*_CITY_) we use a subgrid representation of urban pixels that enables assessing local urban climate impacts undiluted by rural areas in the same 20 km grid square. T_CITY_ is near-surface temperatures at 2 meters of height and corresponds more directly with temperatures experienced by city dwellers (see Krayenhoff et al. (2018) for more detailed information about WRF simulation inputs and outputs).

In order to provide observational evidence indicating historical changes in CDHEs as well as establishing the performance validity of the model for simulation of the joint probability of high temperature and low precipitation events for each city we obtained observed daily maximum temperature data from the closest weather station to grid cells that represent 50 cities with available observed precipitation and temperature data from 1950 to 2020 from NOAA’s National Climatic Data Center (NCDC^51^, Table S1).

### Definition of CDHE events

Dry events are defined as consecutive days with daily total precipitation less than one millimeter (CDD)^69^. The CDD index, recommended by The Expert Team on Climate Change Detection and Indices (ETCCDI) is a useful and simple index for characterizing short-term dry conditions both in terms of frequency and duration^70–73^. Hot events are defined as periods of consecutive days with the daily maximum temperature above the 90th percentile of the daily maximum of the baseline period during the extended summer^48,55,74,75^. If the 90th percentile is lower than 30 °C, the threshold is set to 30 °C^34^. Subsequently, CDHE events are identified based on the concurrence of hot and dry events^27,37,50^. The duration of a CDHE event refers to the number of consecutive days that meet the hot and dry conditions. It should be noted that we allow for short breaks of a cooler day (1 day) within the heatwave event since heatwave events may often continue after a break of a day and using the definition of consecutive hot days could underestimate the actual length of heat waves. Thus, we use a heat wave definition that allows for one break day^74,76^, which also ensures we obtain independent events^55,77^.

During a 10-year period, the occurrence of a CDHE is defined as a binary variable such that if the condition of the hot and dry day is met, CDHE is equal to 1; otherwise is equal to 0^37^. Then, the consecutive days have been summed to represent the CDHE events with different durations. In other words, the duration of CDHE events is defined as the length of consecutive days with maximum temperature exceeding 90th percentile of the daily maximum temperature during the extended summer and daily total precipitation >1 millimeter.

CDHE events are identified for the climate model simulations during the contemporary period (2000–2009 climate forcing with 2010 urban extent), the end-of-century period under climate effect (2090–2099 CESM RCP 8.5 climate forcing with 2010 urban development), the end-of-century period under urban effect (2000–2009 climate forcing with ICLUS A2 2100 urban development), the end-of-century period under urban-climate total effect (2090–2099 CESM RCP 8.5 climate forcing with ICLUS A2 2100 urban development) as well as observed data obtained from NOAA weather stations. In this fashion, we first provide observational evidence indicating historical changes in CDHEs, then establish the performance validity of WRF for simulation of the joint probability of high temperature and low precipitation events and finally evaluate the changes in the frequency and duration of CDHE events in 50 major US cities under combinations of climate forcing (the start-of-century versus the end-of-century) and urban development (ICLUS 2010 versus A2 2100 urban development). Note that our focus is on the extended summer (May–October) season.

### Distribution tests

We use the two-sample KS test to assess differences between the CDFs of CDHE events based on (1) start-of-century observed and simulated climate data, and (2) start-of-century and end-of-century simulated climate data. The former evaluates the accuracy of the WRF climate contemporary simulation in characterization of CDHE events against observed climate data. The latter indicates if there are significant changes in probabilistic characteristics of start- and end-of-century CDHE events. KS is a nonparametric test comparing the CDF of two data sets based on the distance between their empirical distribution functions. The null hypothesis is that the two distribution functions come from the same distribution at a certain significance level (here, *α* = 0.05).

### Return period of CDHE event

We rely on extreme value theory to model the frequency of CDHE events using a generalized Pareto distribution. If the random variable $X$ represents the number of CDHE days, a random variable ${X{{\hbox{'}}}}={X|X}\ge 2$ is considered as a CDHE event and can be fitted to generalized Pareto distribution (GPD) with cumulative distribution function as follows:

1 $${G}_{u,\alpha,\xi }(x)={{\Pr }}\left(X\le x{{{{{\rm{|}}}}}}X \, > \, u\right)=\left\{\begin{array}{cc}1-{\left(1+\xi \frac{x-u}{\alpha }\right)}^{-\frac{1}{\xi }} & {if}\xi \, \ne \, 0\\ 1-{{\exp }}\left(-\frac{x-u}{\alpha }\right) & {if}\xi=0\end{array}\right.$$

where *u* is equal to 2, representing at least 2 consecutive hot and dry days for the definition of CDHE events, and *x* is equal to $u+1$, $u+2$. $\alpha$ and $\xi$ denote the scale and shape of the GPD distribution. Subsequently, the return period (T) of an event with a duration of D can be calculated as follows:

2 $$T(D)=\frac{1}{\varphi [1-{G}_{u,\alpha,\xi }\left(D\right)]}$$

where $1-{G}_{u,\alpha,\xi }(D)$ is the probability of an event exceeding a duration of D and $\varphi$ denotes the ratio of the total number of events to the total number of observation years.

Frequency amplification is defined as the ratio of the contemporary return period ${T}_{c}(D)$ to the future return period ${T}_{F}(D)$ of CDHE event with a duration $D$:

3 $${Frequency\; Amplification}=\frac{{T}_{c}(D)}{{T}_{F}(D)}$$

## Supplementary information

Supplementary Information

## Data Availability

Regional climate simulation output data used in this study are accessible at: https://dataverse.asu.edu/dataverse/USRegClimateChgAssess;jsessionid=0b4c6312abc7bcad996c54a71f38^78^. Simulated future precipitation and temperature data for each city are provided in the Source Data file. Observed climate data can be obtained from https://www.ncdc.noaa.gov/cdo-web/search. Source data are provided with this paper.

## Source data

Source Data

## Code availability

Analysis was performed using custom scripts coded in MATLAB version R2018b. The codes are available through the Zenodo digital repository (10.5281/zenodo.7778828).
