# Supplementary material for: The role of climate change and urban development on compound dry-hot extremes across US cities
Source: Nat Commun. 2023 Jun 14;14:3509. doi: 10.1038/s41467-023-39205-x (PMC10267174; doi:10.1038/s41467-023-39205-x)
Supplement: Supplementary file 1 — Supplementary Information [file 41467_2023_39205_MOESM1_ESM.pdf]

Supplementary Information for:

The Role of Climate Change and Urban Development on Compound Dry-Hot Extremes Across U.S. Cities

Mahshid Ghanbari[1*], Mazdak Arabi[1], Matei Georgescu[2,3], Ashley M. Broadbent[2,4]

[1]Civil and Environmental Engineering Department, Colorado State University, Fort Collins, CO, USA.

[2]School of Geographical Sciences and Urban Planning, Arizona State University, Tempe, AZ, USA.

[3]Urban Climate Research Center, Arizona State University, Tempe, AZ, USA.

[4]National Institute of Weather and Atmospheric Research, Wellington, New Zealand.

*Corresponding author. email: (mahshid.ghanbari@colostate.edu)

Supplementary Text

Figure S1 illustrates the temperature threshold used to define hot days in each city, which is the 90th percentile of the daily maximum temperature of extended summer (May-October) during the contemporary period 2000-2009.

Figure S2 illustrates the P-value of the Kolmogorov-Smirnov (KS) test between CDHE events based on observed and simulated climate data. Using the KS test, we evaluate the distribution of contemporary CDHE events based on 10 years of observed temperature and precipitation data against those based on WRF simulation data during the period of 2000-2009.

Figures S3 to S52 provide detailed analysis information for each station as follows:

(Top panel) presents the empirical distribution function (ECD) of CDHE events based on contemporary observed and WRF simulated climate data as well as future WRF simulated climate data with consideration of the urban-climate total effect.

(Middle panel) presents the boxplots of CDHE events based on contemporary WRF climate data as well as future WRF simulated climate data with consideration of the urban effect, climate effect, and urban-climate total effect.

(Bottom panel) presents the return period return level plot for CDHE events based on contemporary WRF climate data as well as future WRF simulated climate data with consideration of the urban effect, climate effect, and urban-climate total effect.

Table S1. Presents the name of major U.S. cities considered in the study along with their corresponding weather station name and location (Lat/Long).

Supplementary Figures

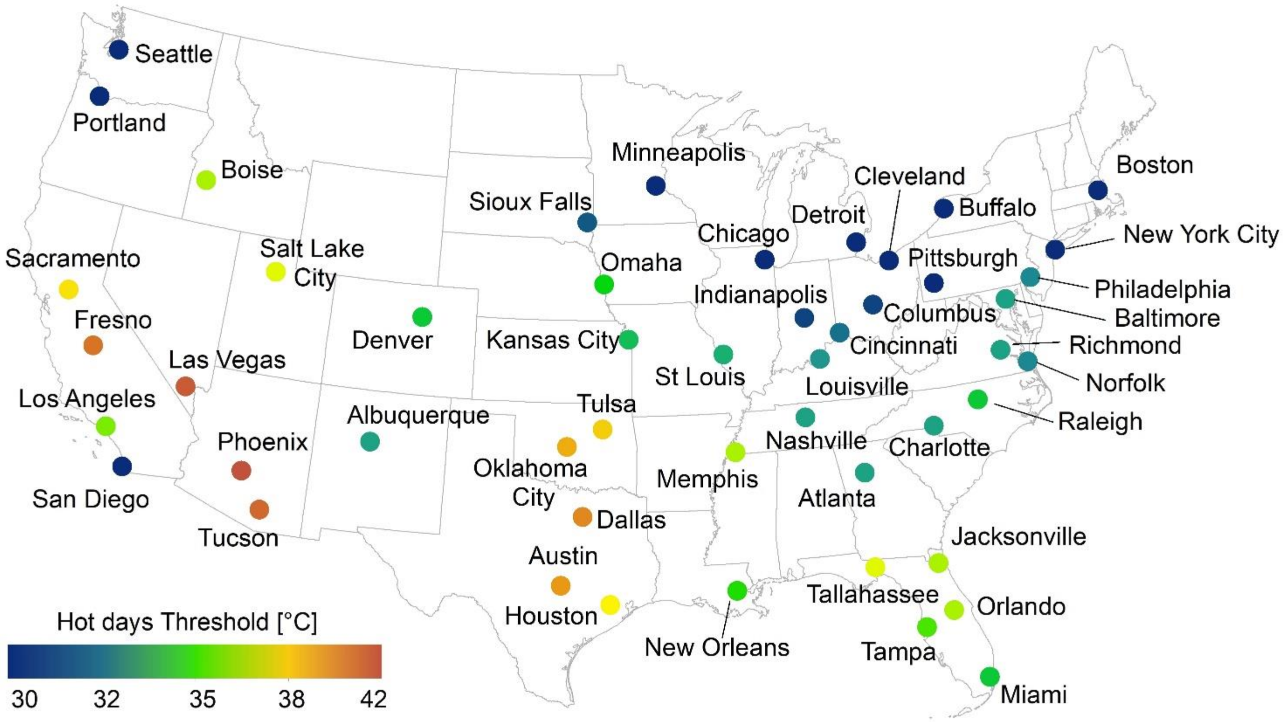

Figure S1. The 90th percentile of the daily maximum temperature of extended summer (May-October) during the contemporary period 2000-2009. If the 90th percentile is lower than 30°C, the threshold is set to 30°C.

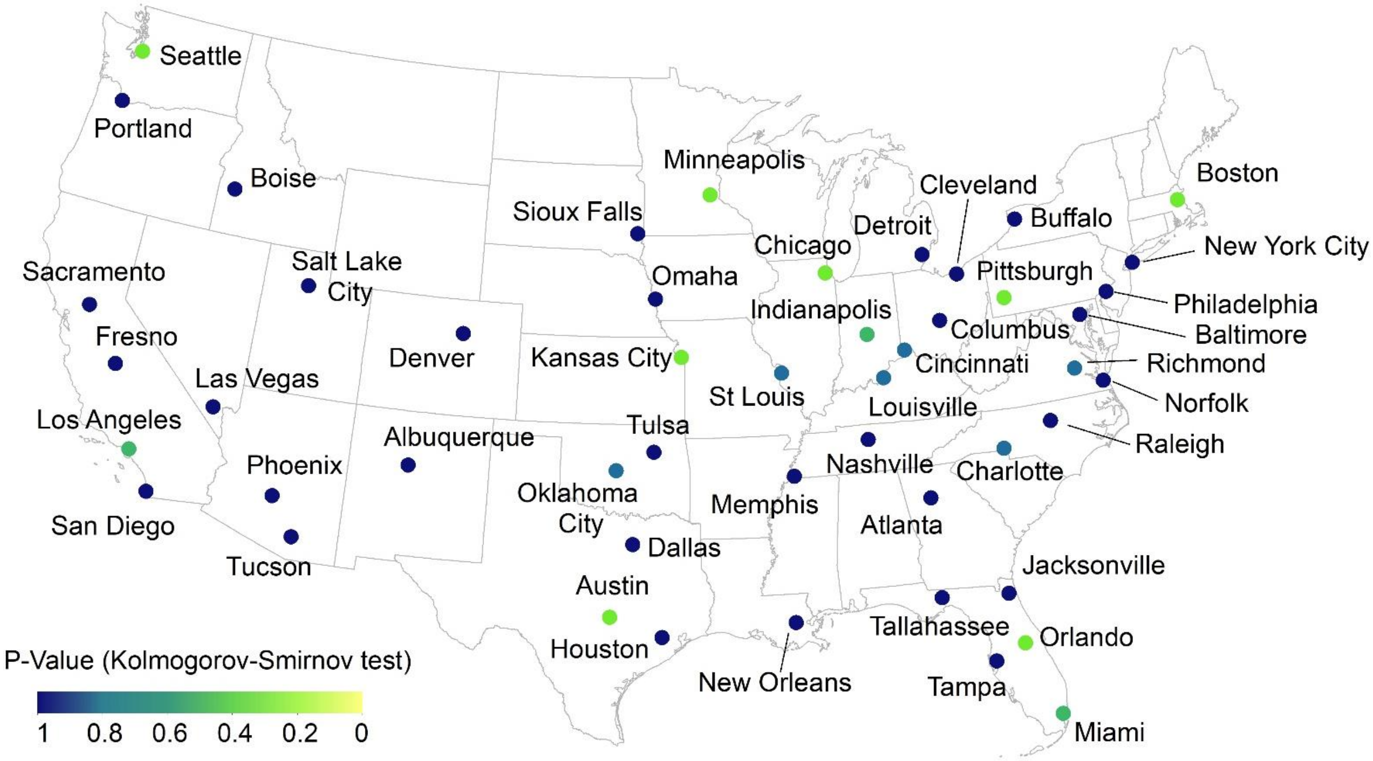

Figure S2. The P-value of the Kolmogorov-Smirnov test between CDHE events based on observed and simulated climate data (The closer the p-value is to 1, the better the fit between the observed data and model simulations)

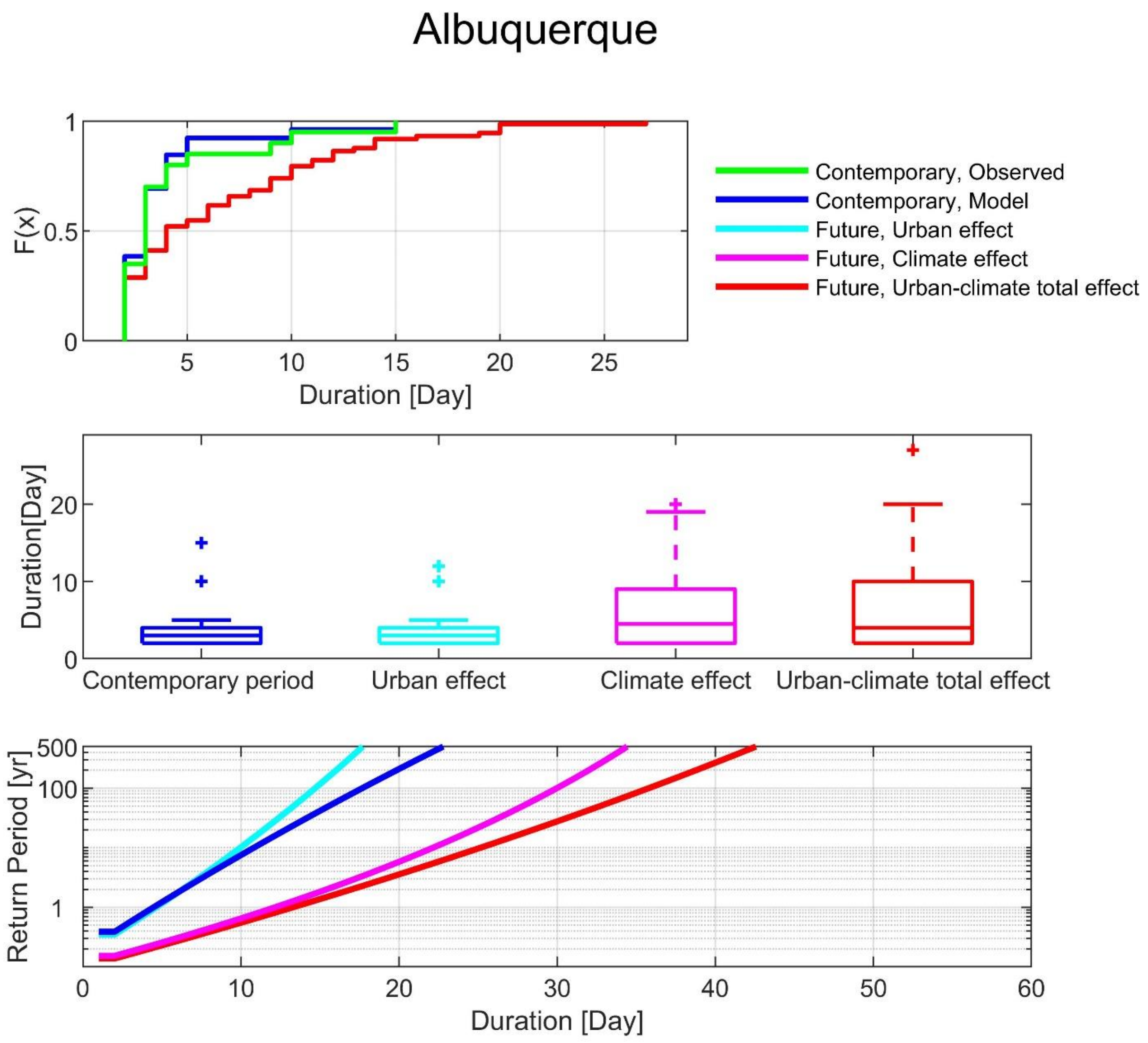

Figure S3. (Top panel) Cumulative distribution function (CDF) of CDHE events based on contemporary observed/model as well as future with consideration of the urban-climate total effect. (Middle panel) Boxplots of future CDHE events with consideration of the urban, climate, and urban-climate total effect. (Bottom panel) Return period return level plot for CDHE events for the contemporary period and future period with consideration of urban, climate, and urban-climate total effect.

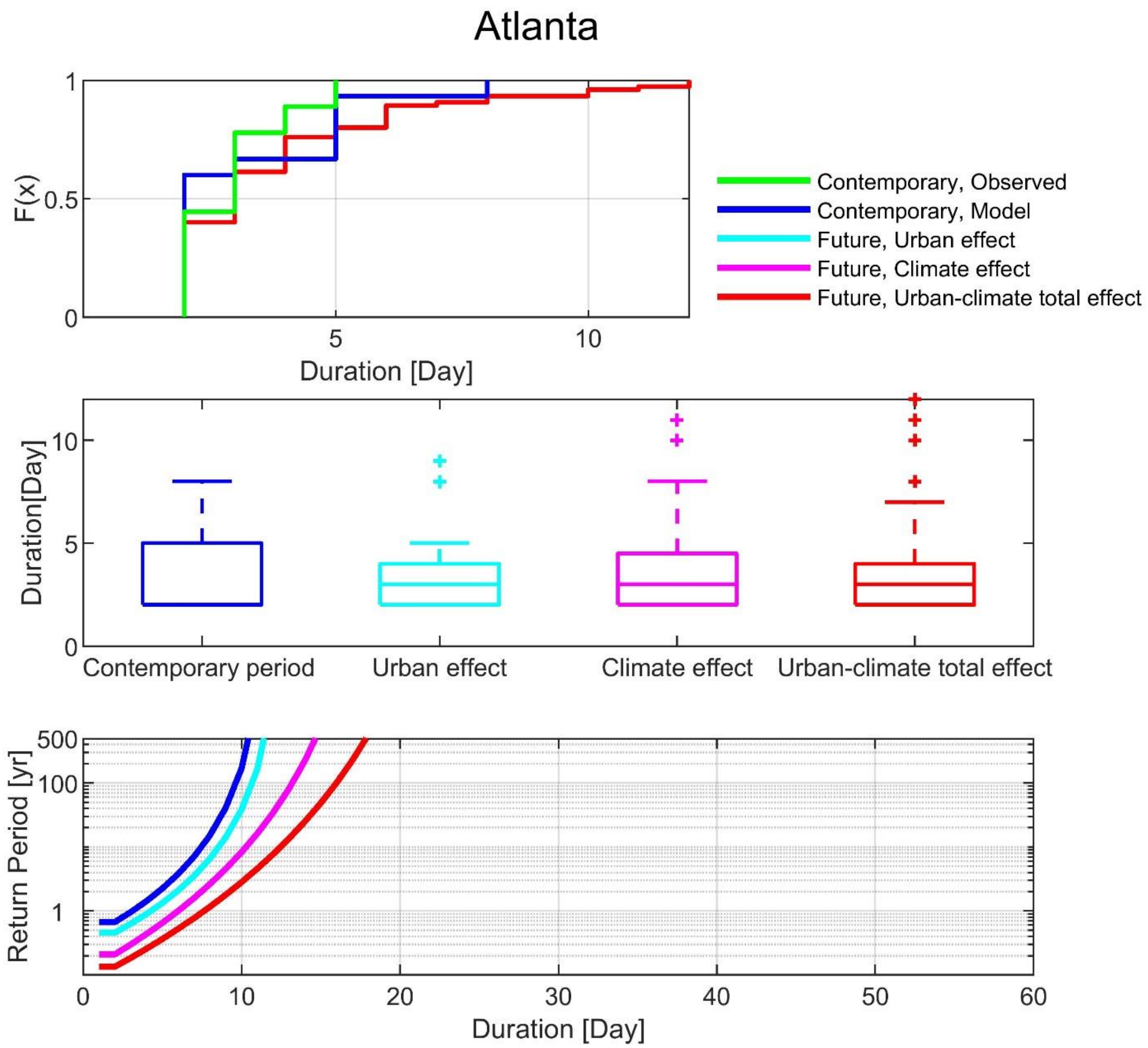

Figure S4. (Top panel) Cumulative distribution function (CDF) of compound dry-hot extreme (CDHE) events based on contemporary observed/model as well as future with consideration of the urban-climate total effect. (Middle panel) Boxplots of future CDHE events with consideration of the urban, climate, and urban-climate total effect. (Bottom panel) Return period return level plot for CDHE events for the contemporary period and future period with consideration of urban, climate, and urban-climate total effect.

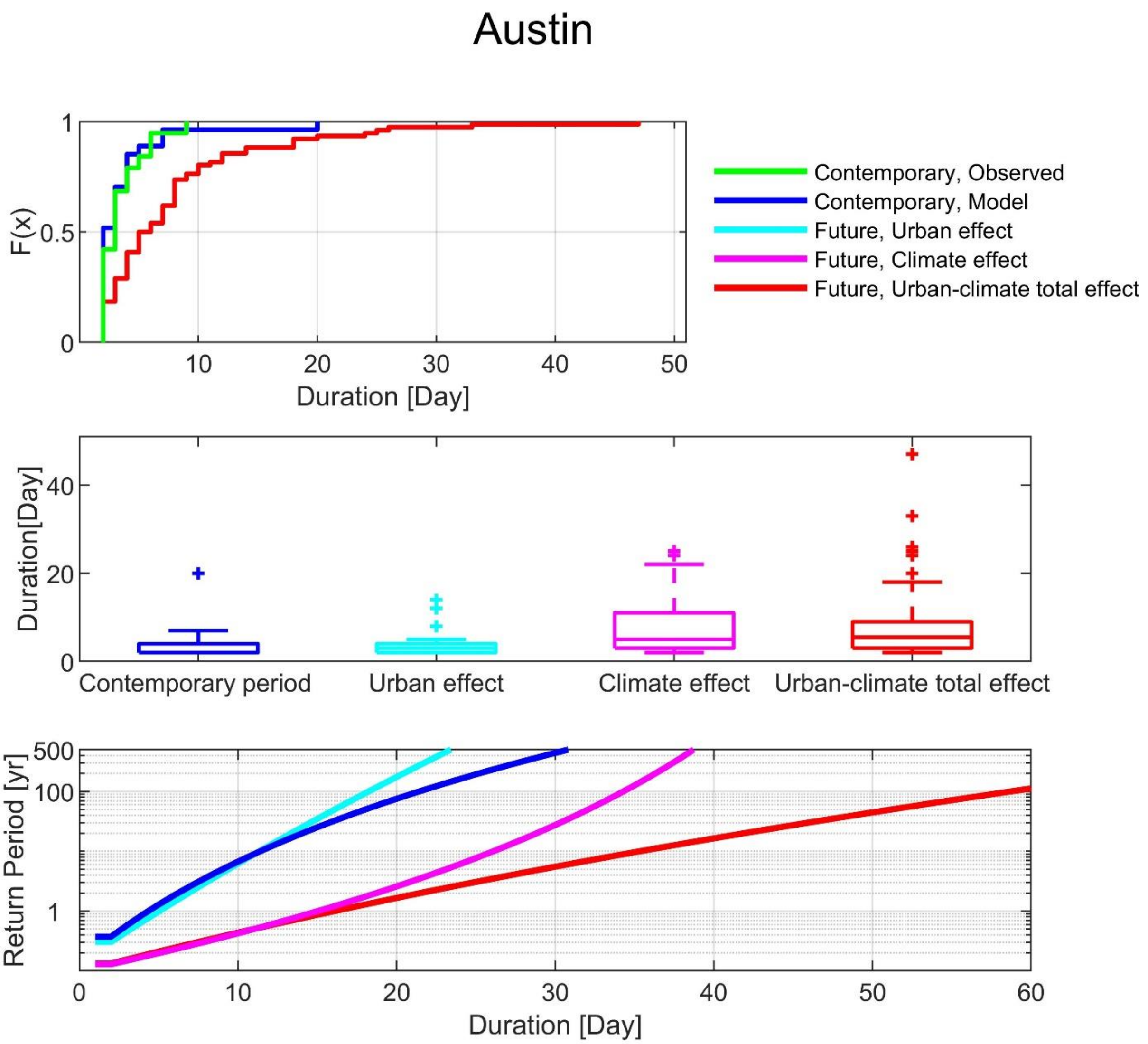

Figure S5. (Top panel) Cumulative distribution function (CDF) of compound dry-hot extreme (CDHE) events based on contemporary observed/model as well as future with consideration of the urban-climate total effect. (Middle panel) Boxplots of future CDHE events with consideration of the urban, climate, and urban-climate total effect. (Bottom panel) Return period return level plot for CDHE events for the contemporary period and future period with consideration of urban, climate, and urban-climate total effect.

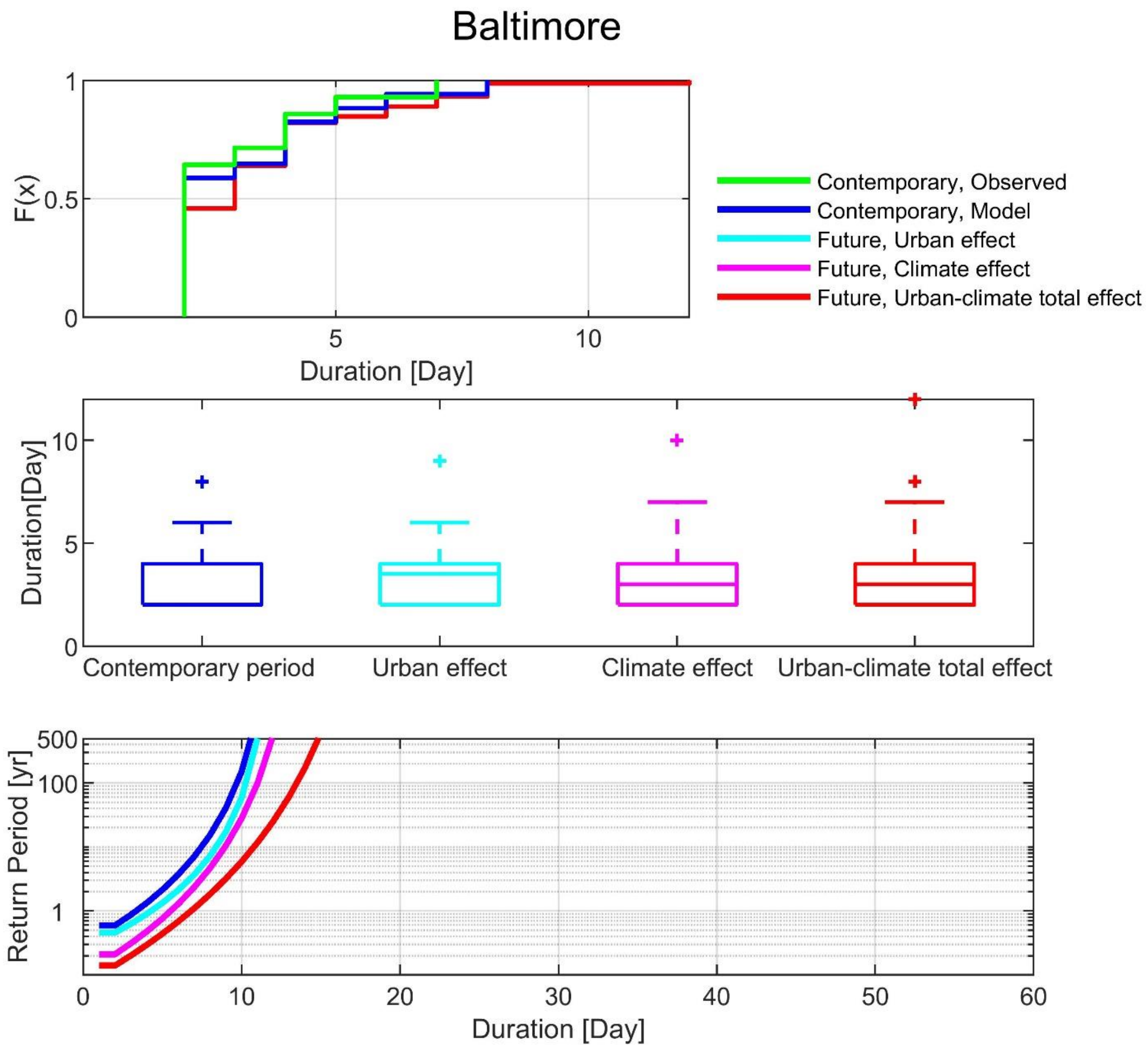

Figure S6. (Top panel) Cumulative distribution function (CDF) of compound dry-hot extreme (CDHE) events based on contemporary observed/model as well as future with consideration of the urban-climate total effect. (Middle panel) Boxplots of future CDHE events with consideration of the urban, climate, and urban-climate total effect. (Bottom panel) Return period return level plot for CDHE events for the contemporary period and future period with consideration of urban, climate, and urban-climate total effect.

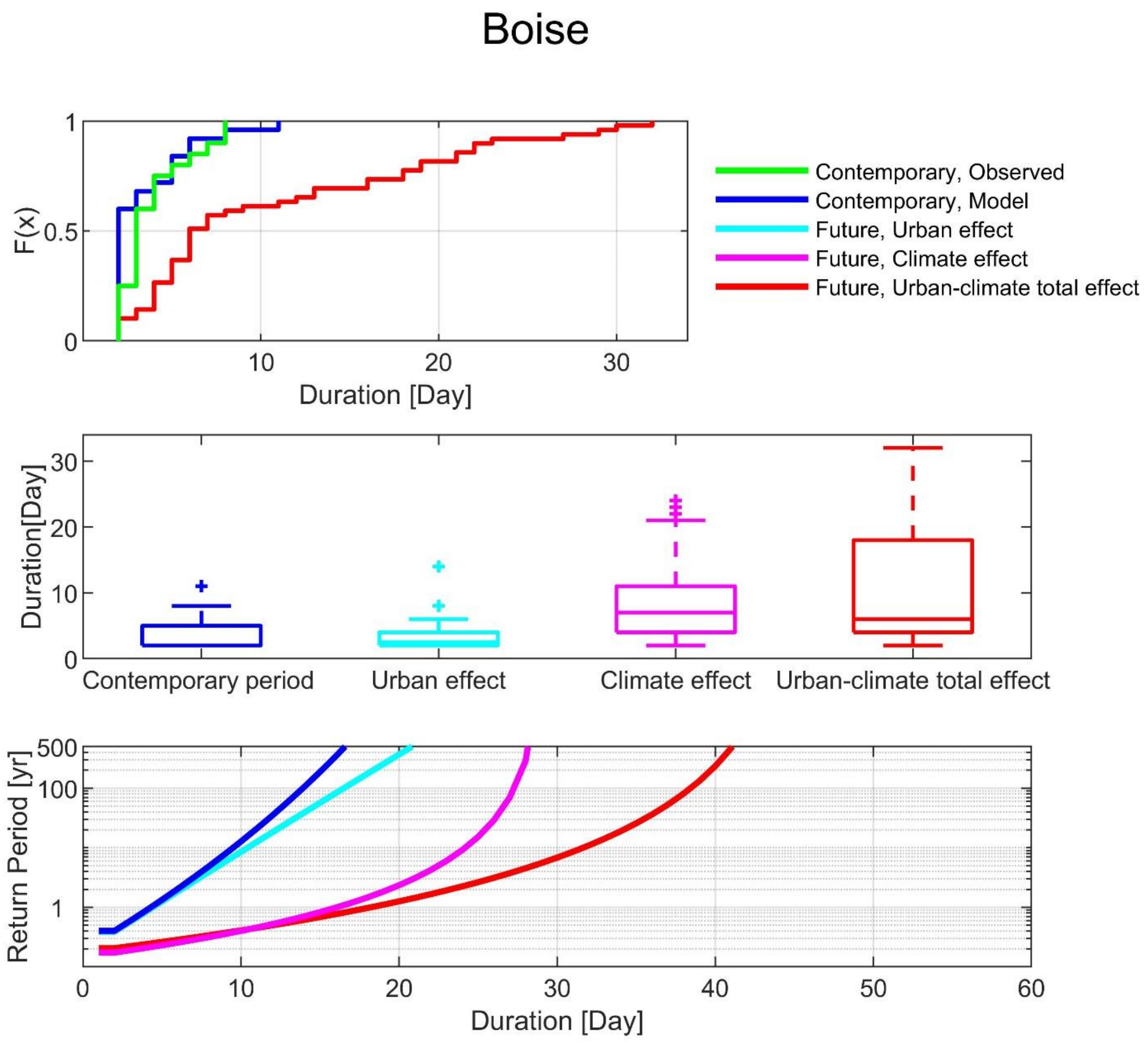

Figure S7. (Top panel) Cumulative distribution function (CDF) of compound dry-hot extreme (CDHE) events based on contemporary observed/model as well as future with consideration of the urban-climate total effect. (Middle panel) Boxplots of future CDHE events with consideration of the urban, climate, and urban-climate total effect. (Bottom panel) Return period return level plot for CDHE events for the contemporary period and future period with consideration of urban, climate, and urban-climate total effect.

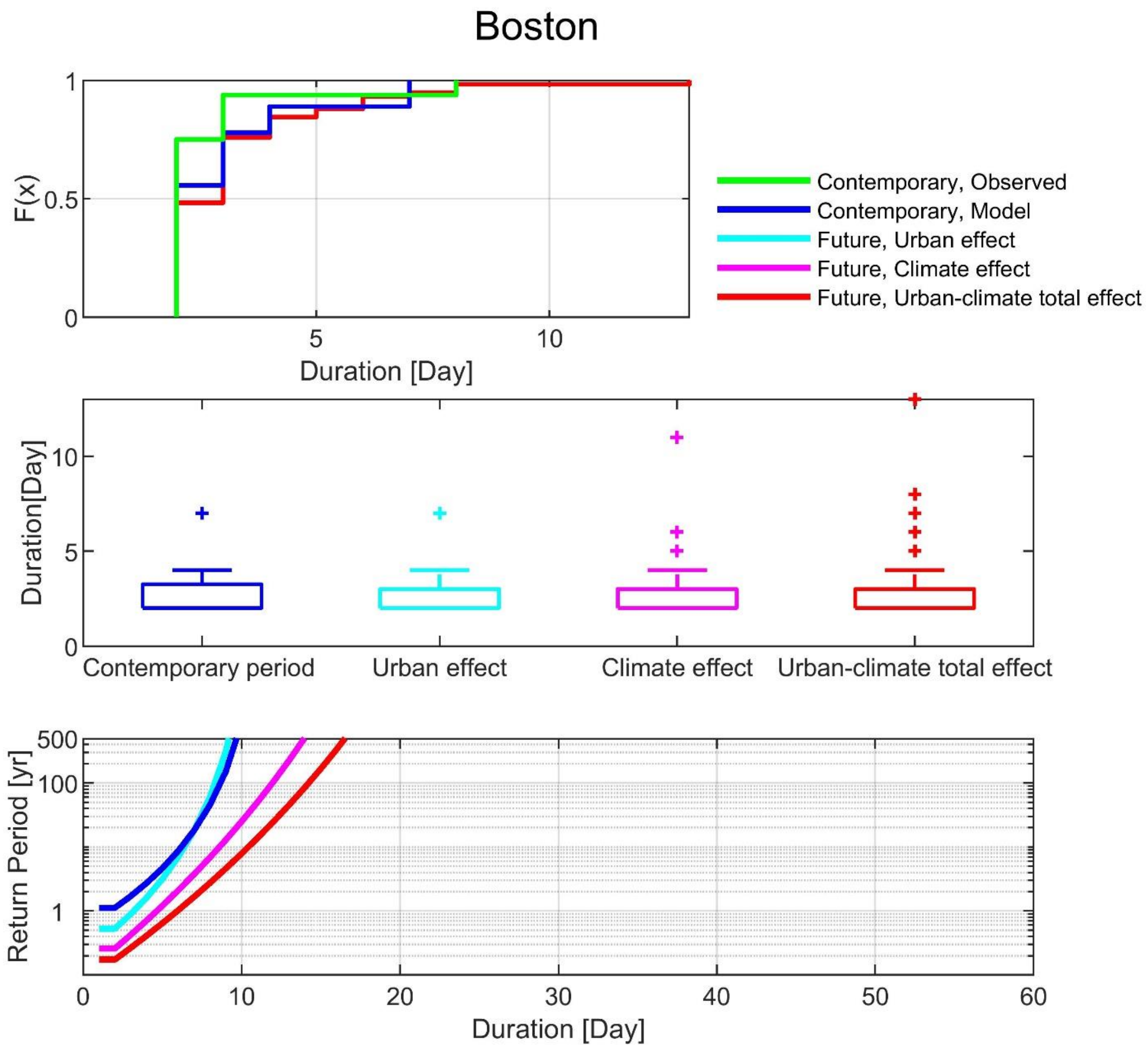

Figure S8. (Top panel) Cumulative distribution function (CDF) of compound dry-hot extreme (CDHE) events based on contemporary observed/model as well as future with consideration of the urban-climate total effect. (Middle panel) Boxplots of future CDHE events with consideration of the urban, climate, and urban-climate total effect. (Bottom panel) Return period return level plot for CDHE events for the contemporary period and future period with consideration of urban, climate, and urban-climate total effect.

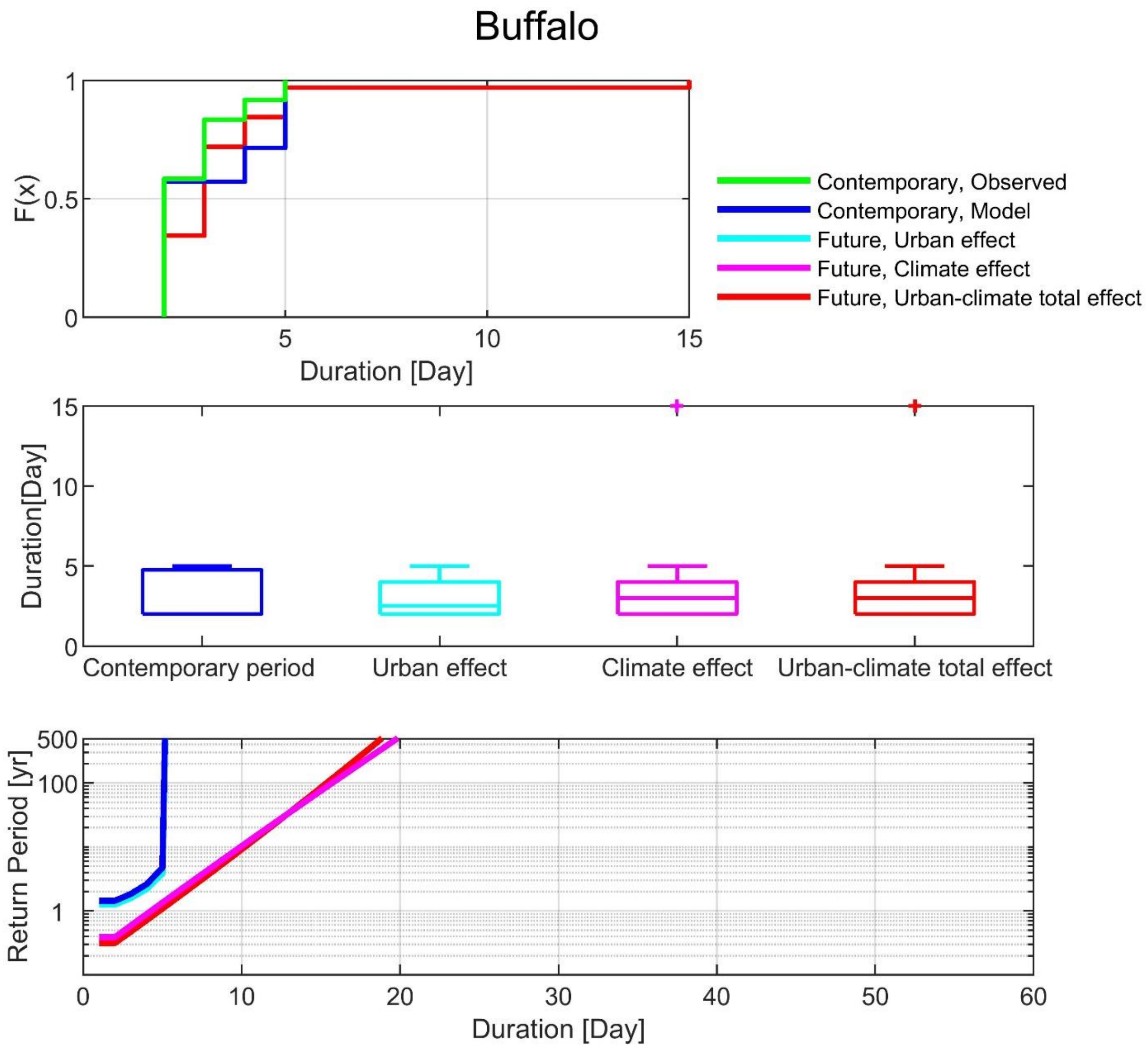

Figure S9. (Top panel) Cumulative distribution function (CDF) of compound dry-hot extreme (CDHE) events based on contemporary observed/model as well as future with consideration of the urban-climate total effect. (Middle panel) Boxplots of future CDHE events with consideration of the urban, climate, and urban-climate total effect. (Bottom panel) Return period return level plot for CDHE events for the contemporary period and future period with consideration of urban, climate, and urban-climate total effect.

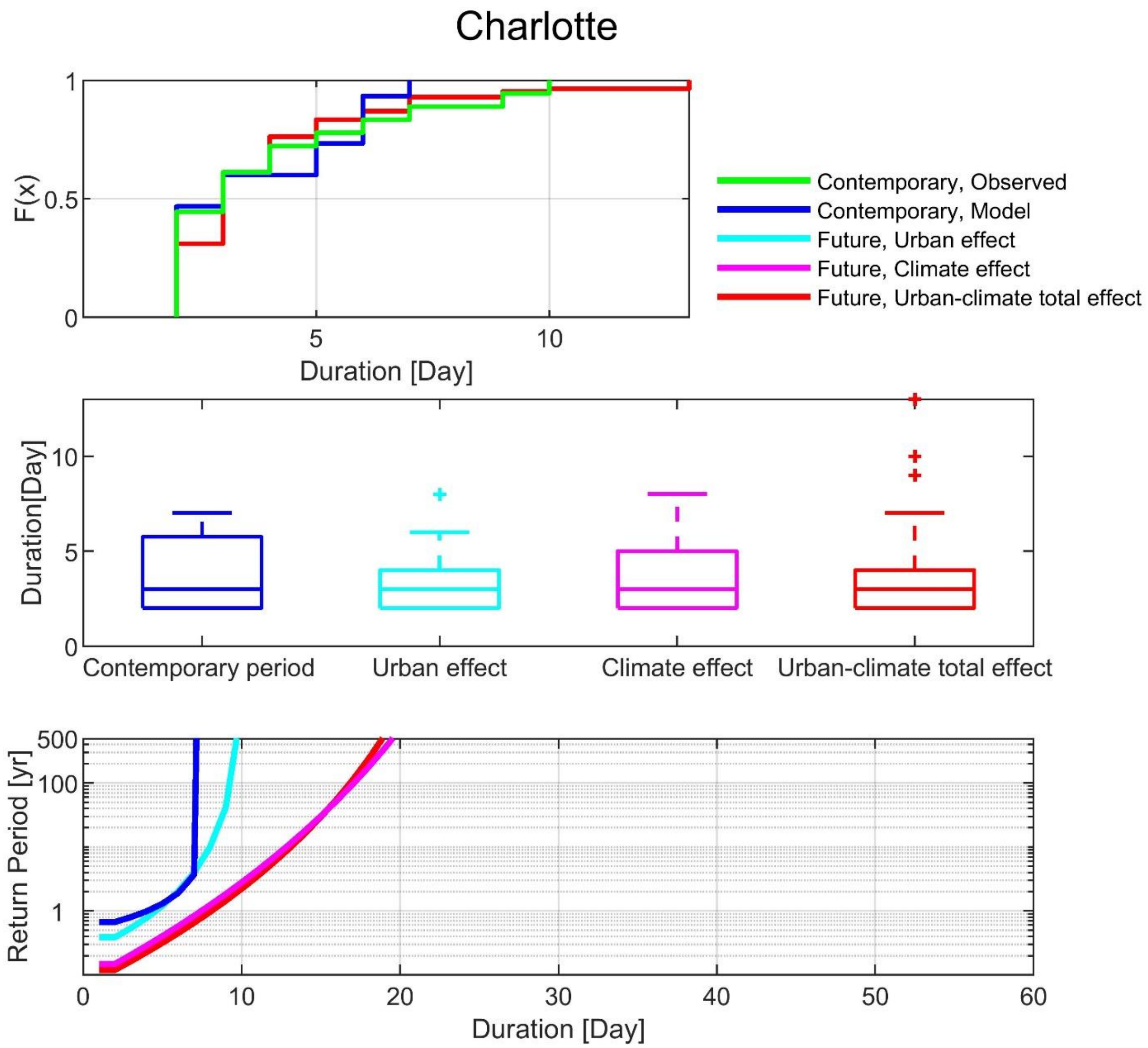

Figure S10. (Top panel) Cumulative distribution function (CDF) of compound dry-hot extreme (CDHE) events based on contemporary observed/model as well as future with consideration of the urban-climate total effect. (Middle panel) Boxplots of future CDHE events with consideration of the urban, climate, and urban-climate total effect. (Bottom panel) Return period return level plot for CDHE events for the contemporary period and future period with consideration of urban, climate, and urban-climate total effect.

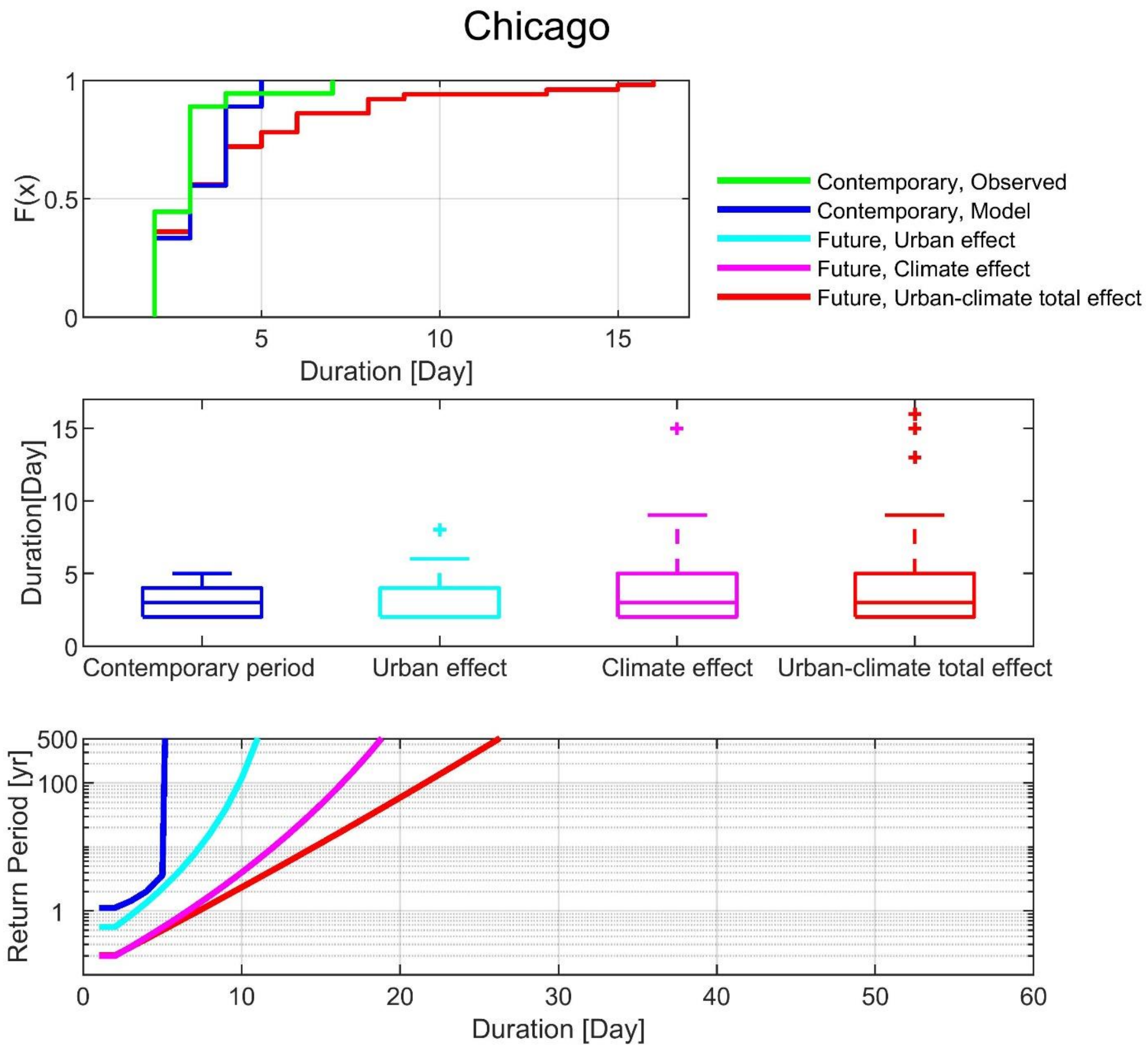

Figure S11. (Top panel) Cumulative distribution function (CDF) of compound dry-hot extreme (CDHE) events based on contemporary observed/model as well as future with consideration of the urban-climate total effect. (Middle panel) Boxplots of future CDHE events with consideration of the urban, climate, and urban-climate total effect. (Bottom panel) Return period return level plot for CDHE events for the contemporary period and future period with consideration of urban, climate, and urban-climate total effect.

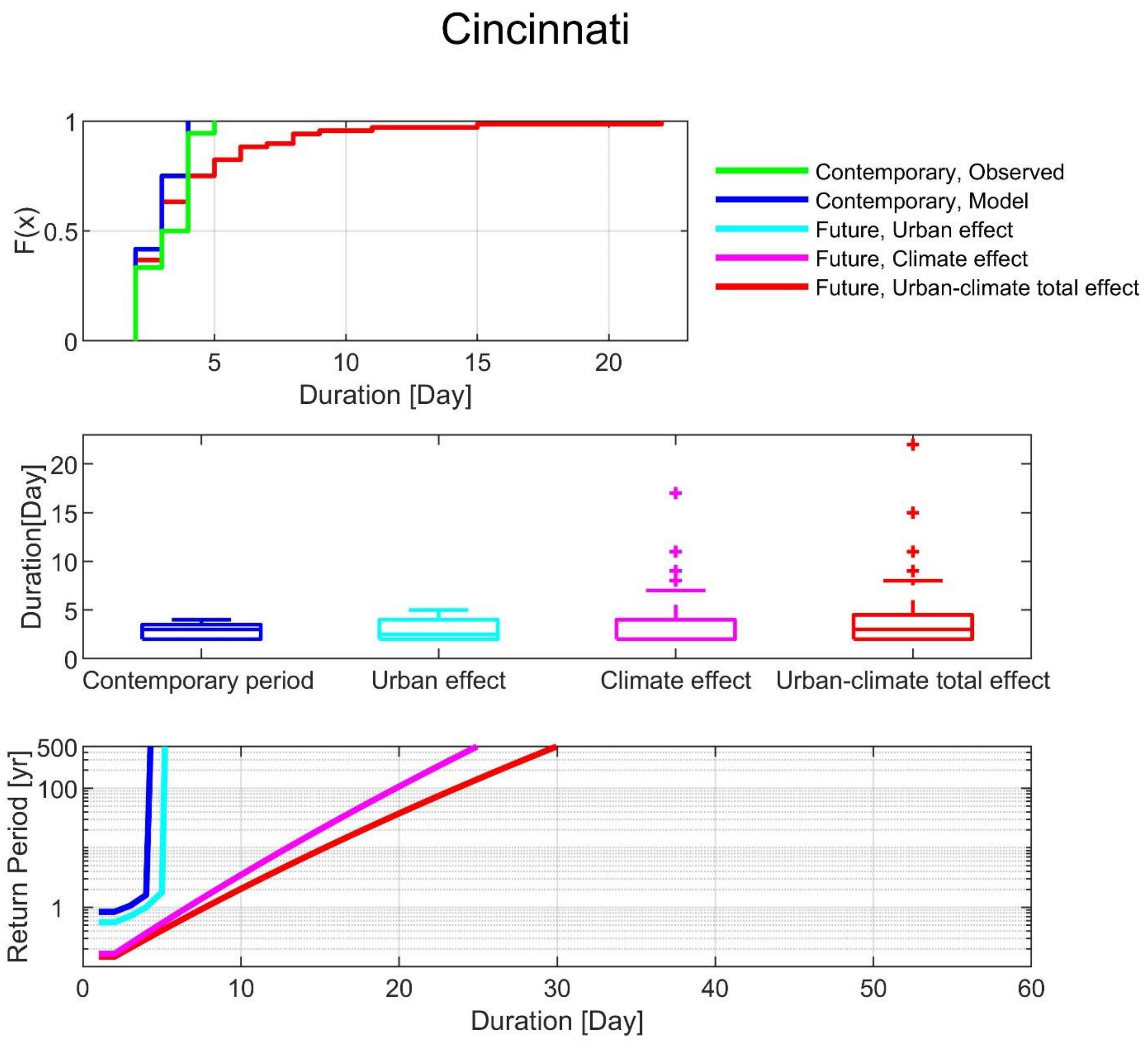

Figure S12. (Top panel) Cumulative distribution function (CDF) of compound dry-hot extreme (CDHE) events based on contemporary observed/model as well as future with consideration of the urban-climate total effect. (Middle panel) Boxplots of future CDHE events with consideration of the urban, climate, and urban-climate total effect. (Bottom panel) Return period return level plot for CDHE events for the contemporary period and future period with consideration of urban, climate, and urban-climate total effect.

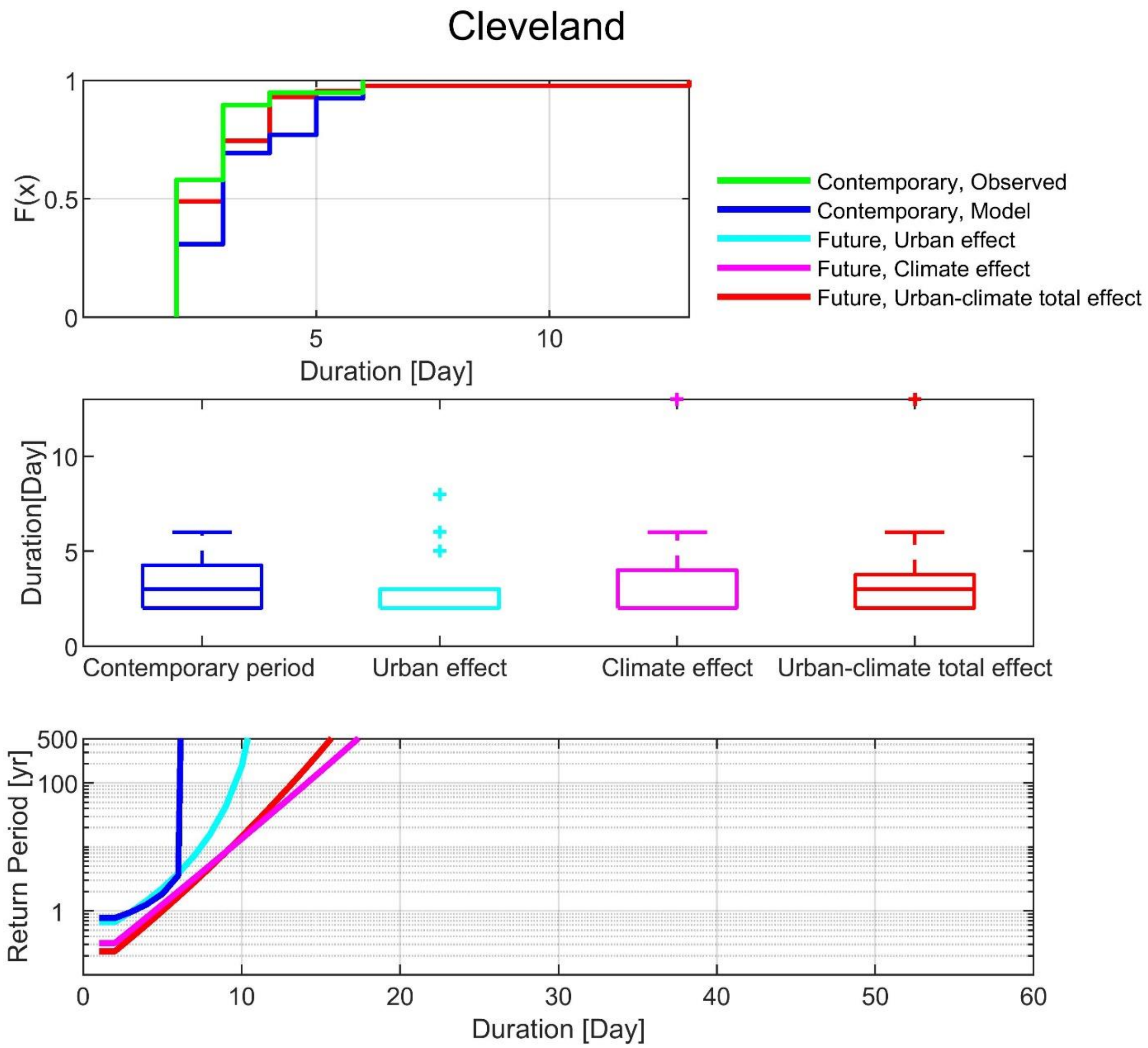

Figure S13. (Top panel) Cumulative distribution function (CDF) of compound dry-hot extreme (CDHE) events based on contemporary observed/model as well as future with consideration of the urban-climate total effect. (Middle panel) Boxplots of future CDHE events with consideration of the urban, climate, and urban-climate total effect. (Bottom panel) Return period return level plot for CDHE events for the contemporary period and future period with consideration of urban, climate, and urban-climate total effect.

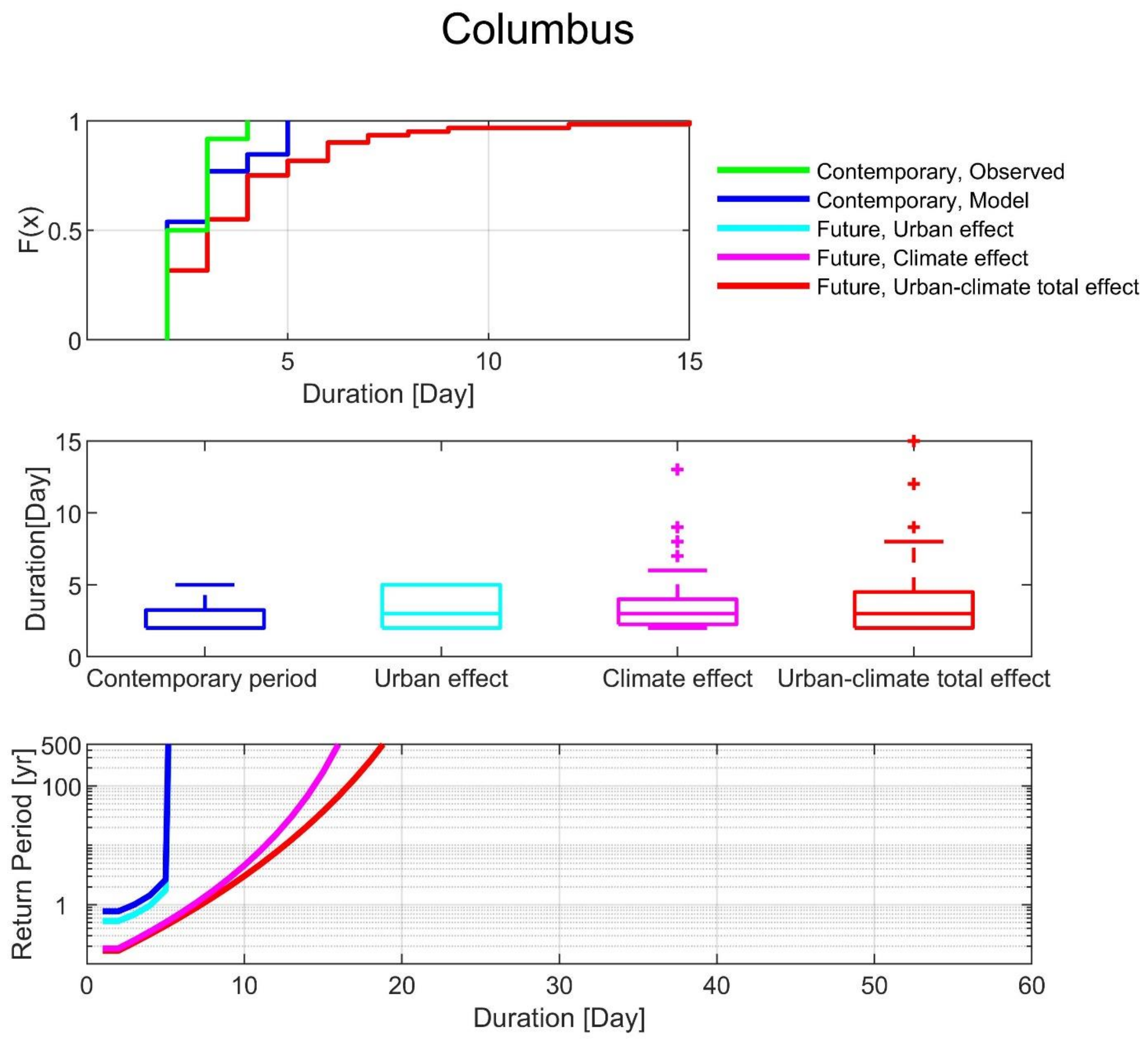

Figure S14. (Top panel) Cumulative distribution function (CDF) of compound dry-hot extreme (CDHE) events based on contemporary observed/model as well as future with consideration of the urban-climate total effect. (Middle panel) Boxplots of future CDHE events with consideration of the urban, climate, and urban-climate total effect. (Bottom panel) Return period return level plot for CDHE events for the contemporary period and future period with consideration of urban, climate, and urban-climate total effect.

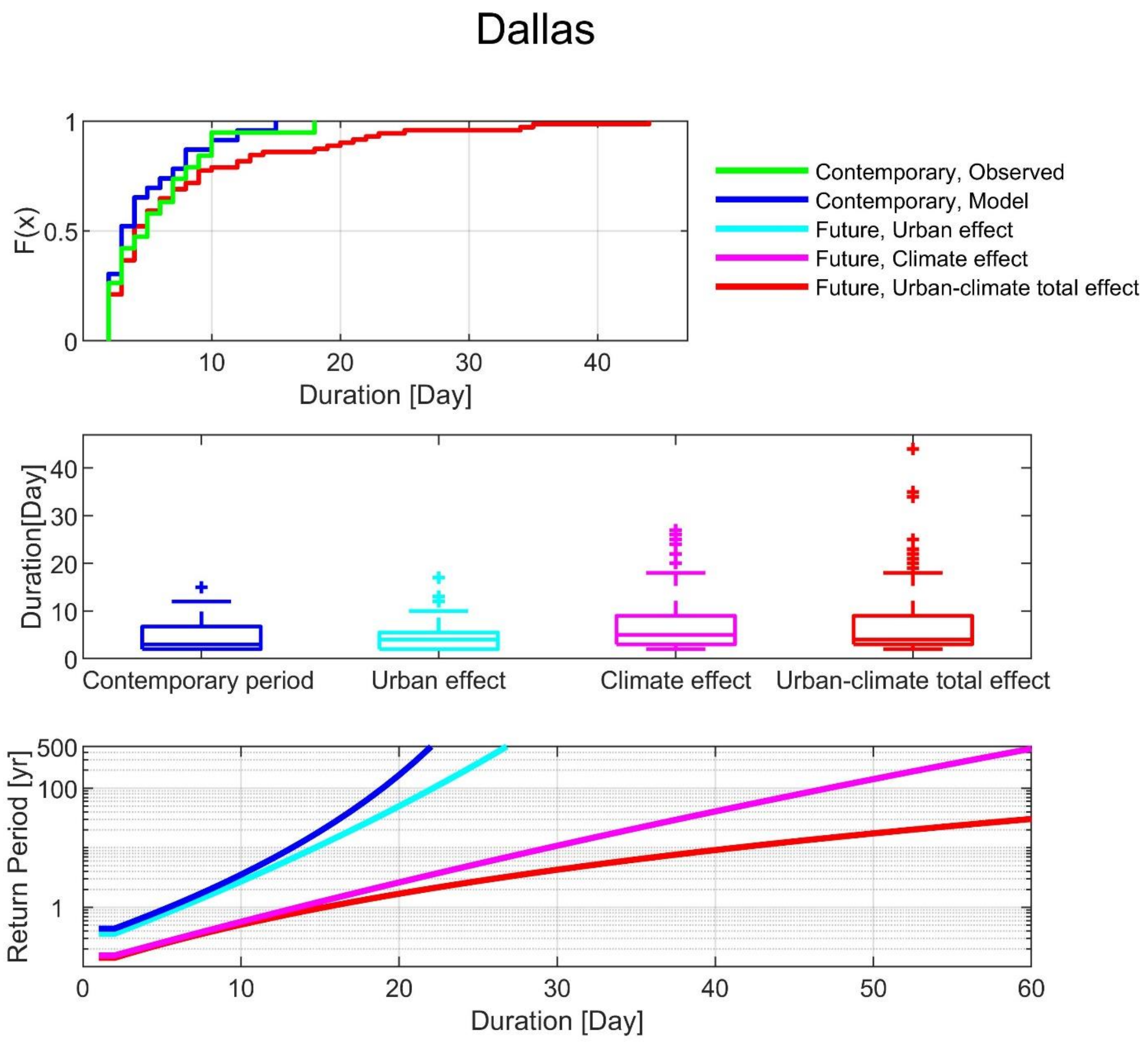

Figure S15. (Top panel) Cumulative distribution function (CDF) of compound dry-hot extreme (CDHE) events based on contemporary observed/model as well as future with consideration of the urban-climate total effect. (Middle panel) Boxplots of future CDHE events with consideration of the urban, climate, and urban-climate total effect. (Bottom panel) Return period return level plot for CDHE events for the contemporary period and future period with consideration of urban, climate, and urban-climate total effect.

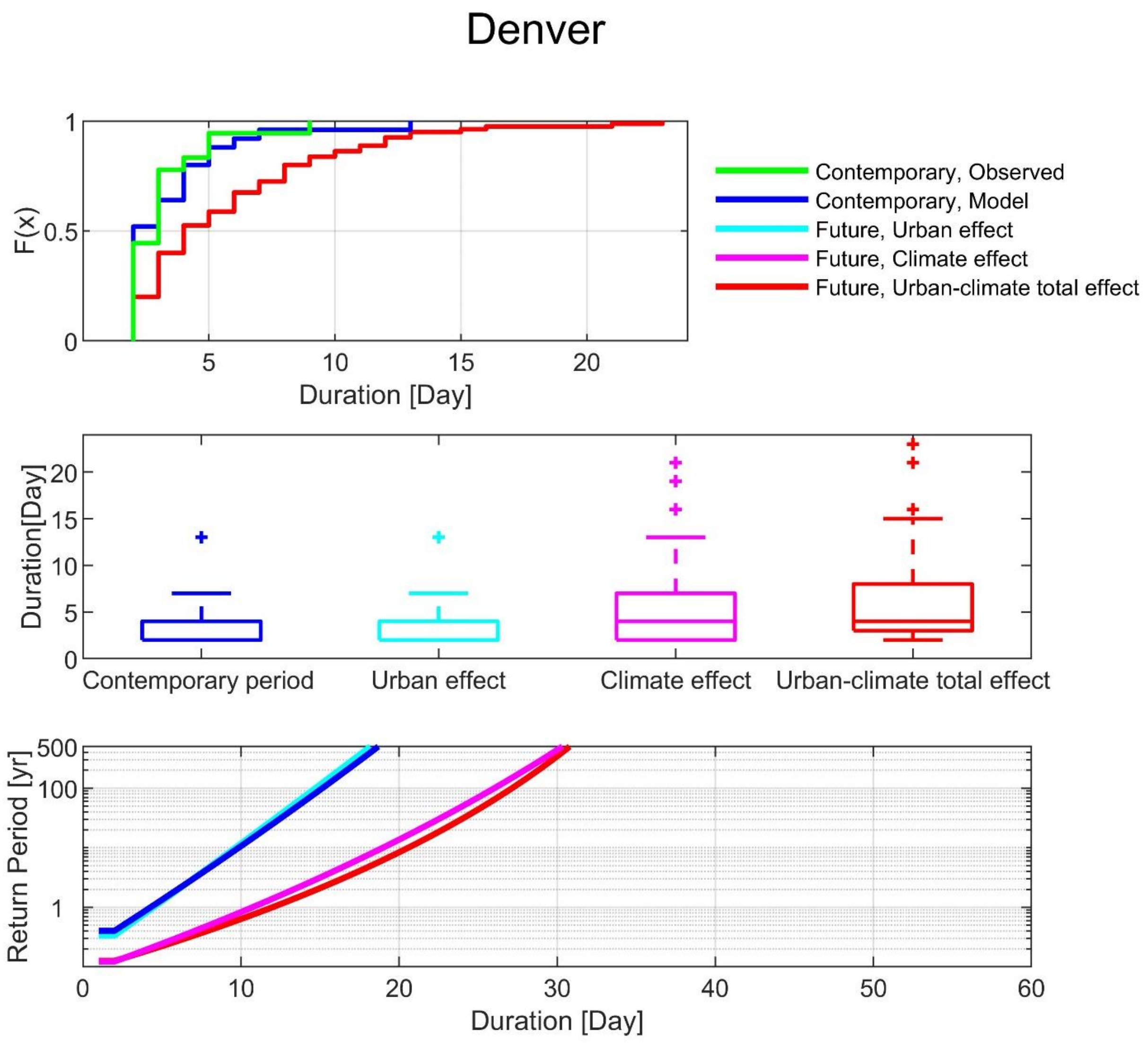

Figure S16. (Top panel) Cumulative distribution function (CDF) of compound dry-hot extreme (CDHE) events based on contemporary observed/model as well as future with consideration of the urban-climate total effect. (Middle panel) Boxplots of future CDHE events with consideration of the urban, climate, and urban-climate total effect. (Bottom panel) Return period return level plot for CDHE events for the contemporary period and future period with consideration of urban, climate, and urban-climate total effect.

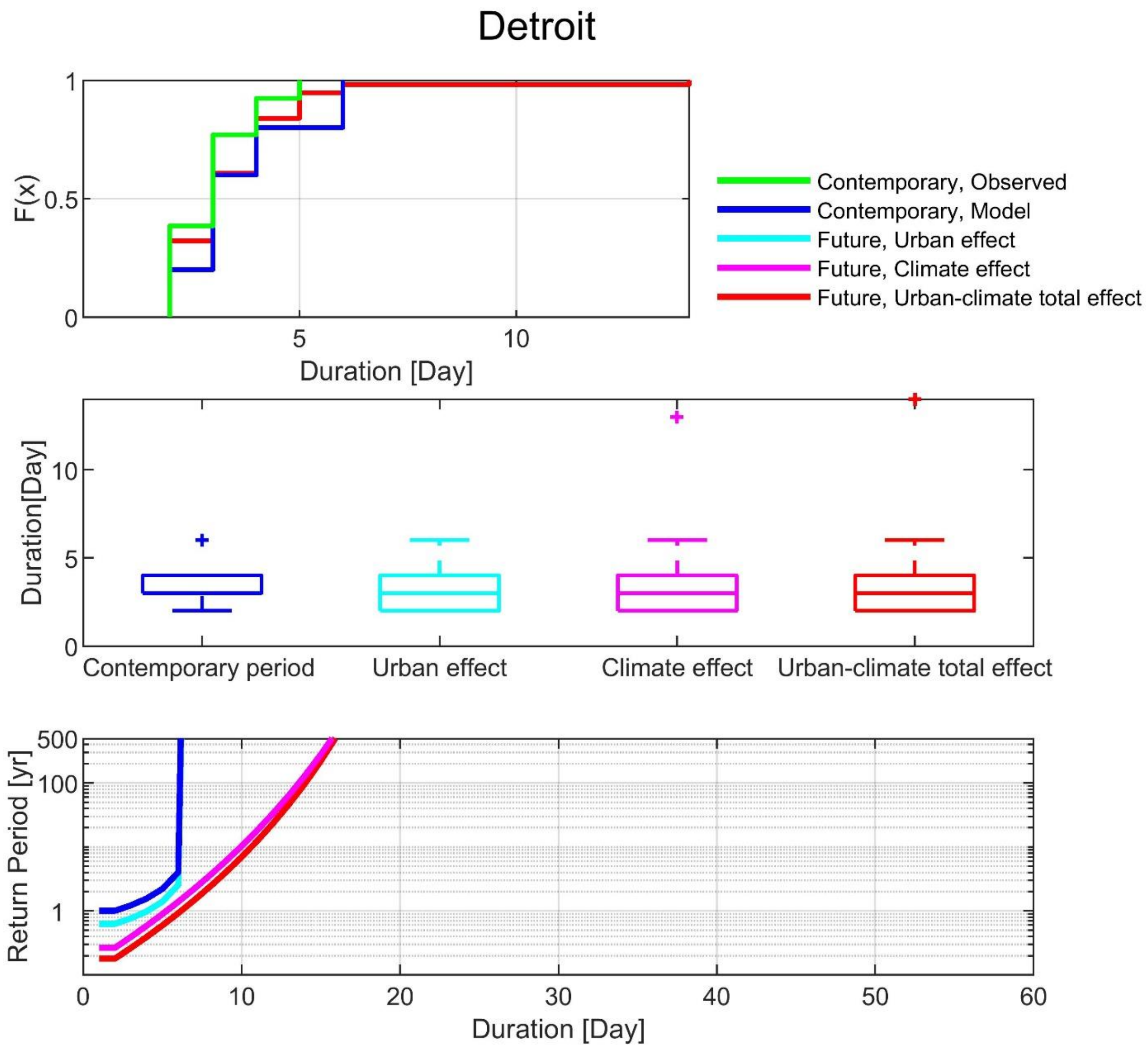

Figure S17. (Top panel) Cumulative distribution function (CDF) of compound dry-hot extreme (CDHE) events based on contemporary observed/model as well as future with consideration of the urban-climate total effect. (Middle panel) Boxplots of future CDHE events with consideration of the urban, climate, and urban-climate total effect. (Bottom panel) Return period return level plot for CDHE events for the contemporary period and future period with consideration of urban, climate, and urban-climate total effect.

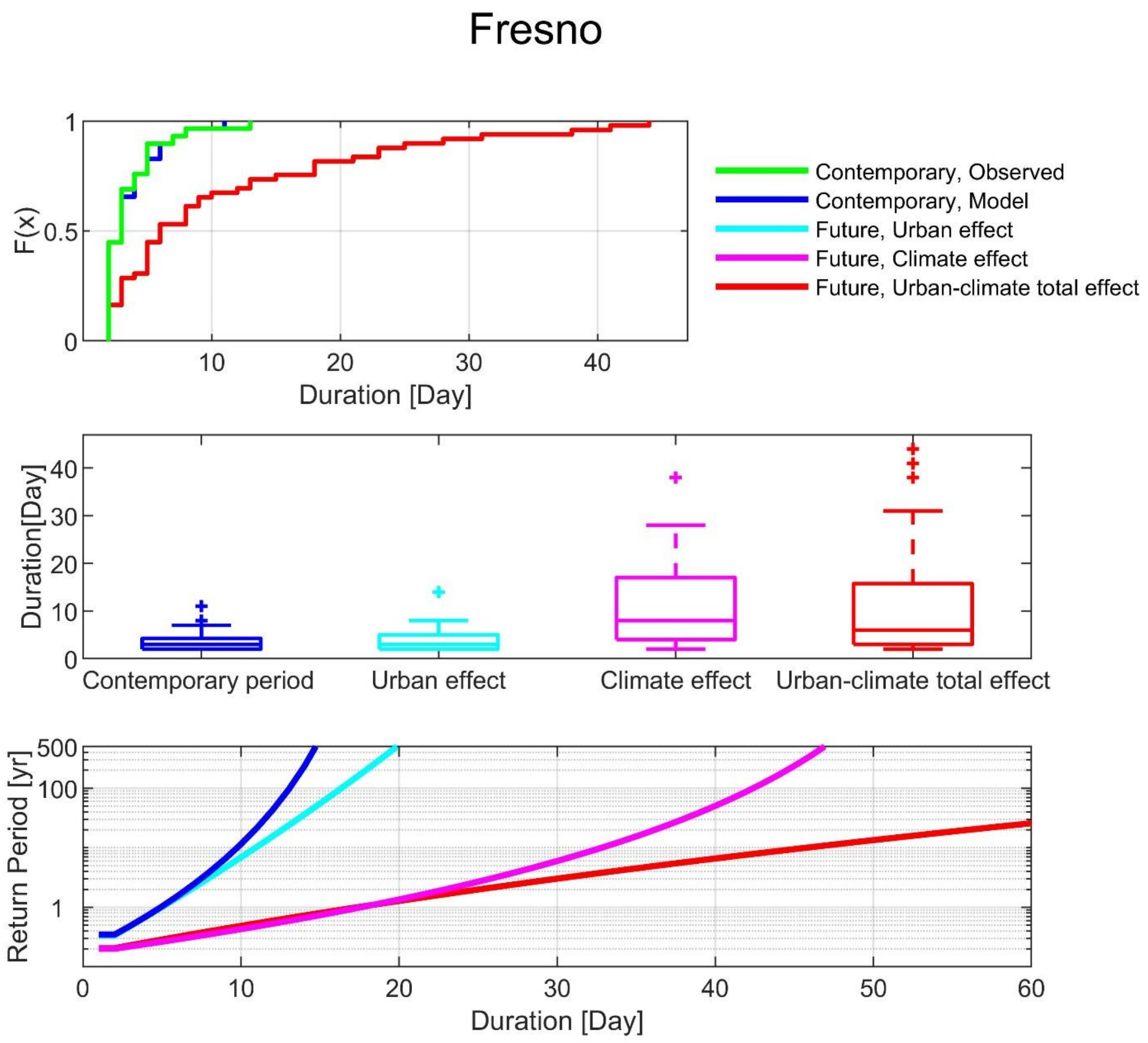

Figure S18. (Top panel) Cumulative distribution function (CDF) of compound dry-hot extreme (CDHE) events based on contemporary observed/model as well as future with consideration of the urban-climate total effect. (Middle panel) Boxplots of future CDHE events with consideration of the urban, climate, and urban-climate total effect. (Bottom panel) Return period return level plot for CDHE events for the contemporary period and future period with consideration of urban, climate, and urban-climate total effect.

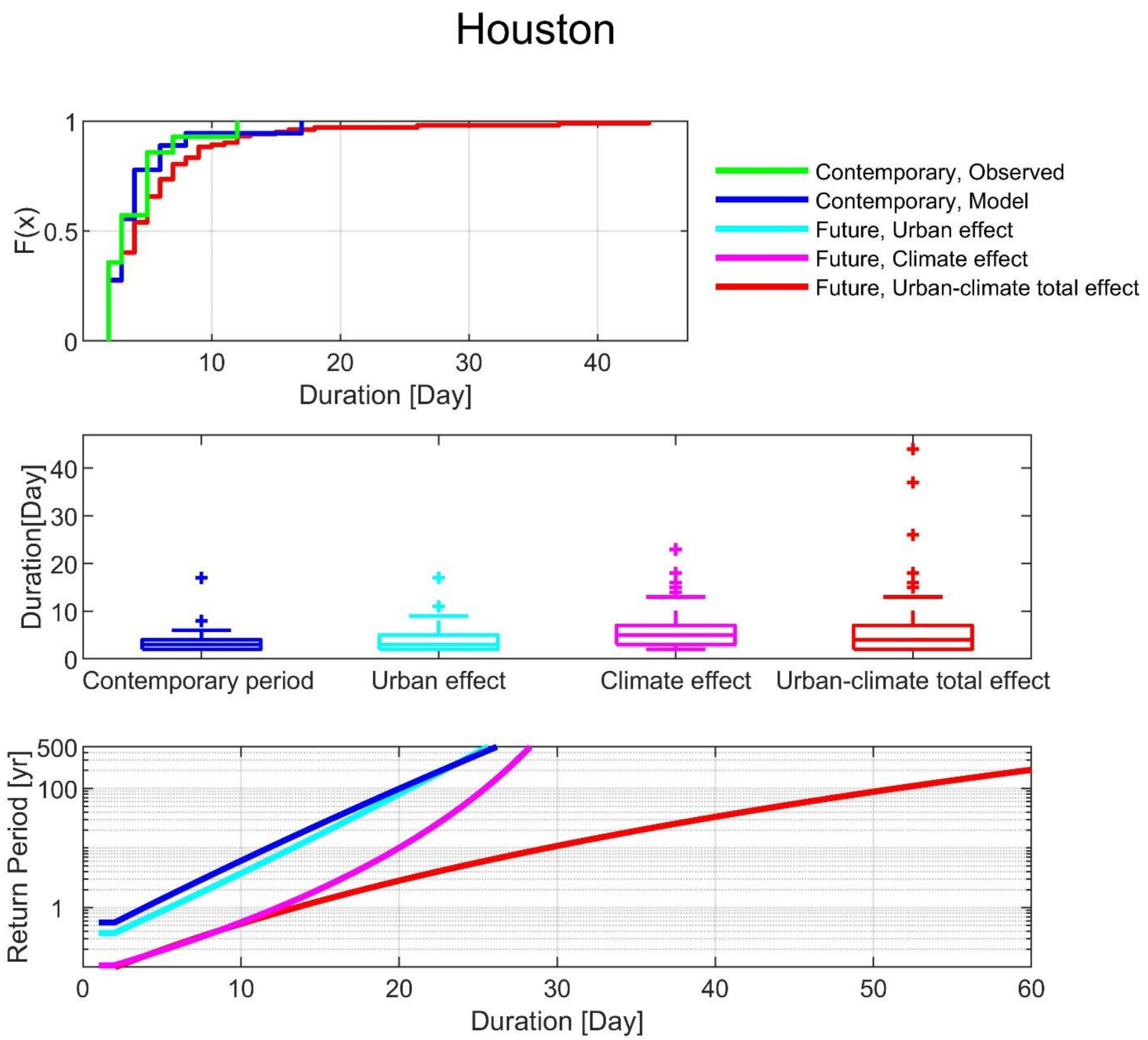

Figure S19. (Top panel) Cumulative distribution function (CDF) of compound dry-hot extreme (CDHE) events based on contemporary observed/model as well as future with consideration of the urban-climate total effect. (Middle panel) Boxplots of future CDHE events with consideration of the urban, climate, and urban-climate total effect. (Bottom panel) Return period return level plot for CDHE events for the contemporary period and future period with consideration of urban, climate, and urban-climate total effect.

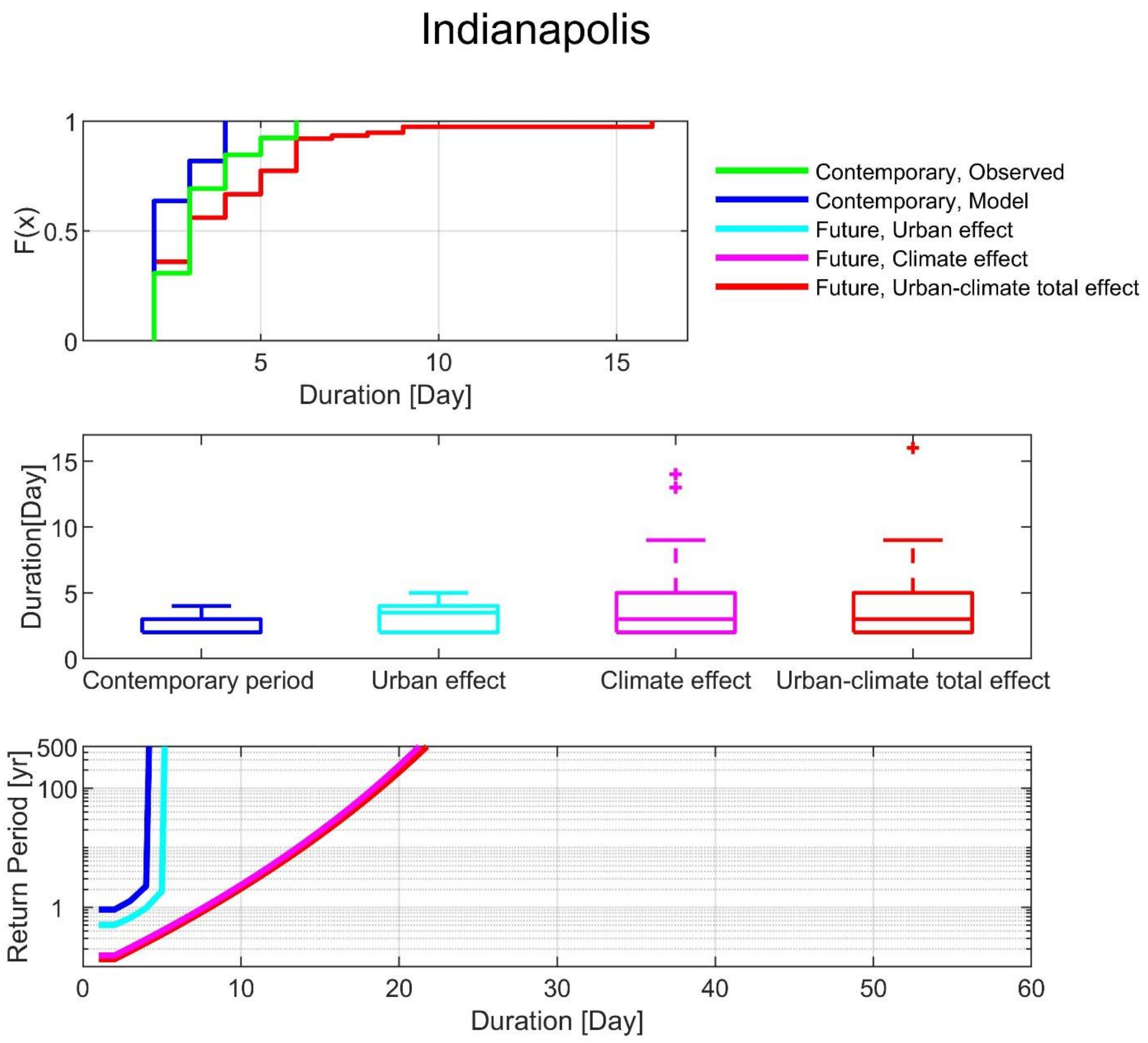

Figure S20. (Top panel) Cumulative distribution function (CDF) of compound dry-hot extreme (CDHE) events based on contemporary observed/model as well as future with consideration of the urban-climate total effect. (Middle panel) Boxplots of future CDHE events with consideration of the urban, climate, and urban-climate total effect. (Bottom panel) Return period return level plot for CDHE events for the contemporary period and future period with consideration of urban, climate, and urban-climate total effect.

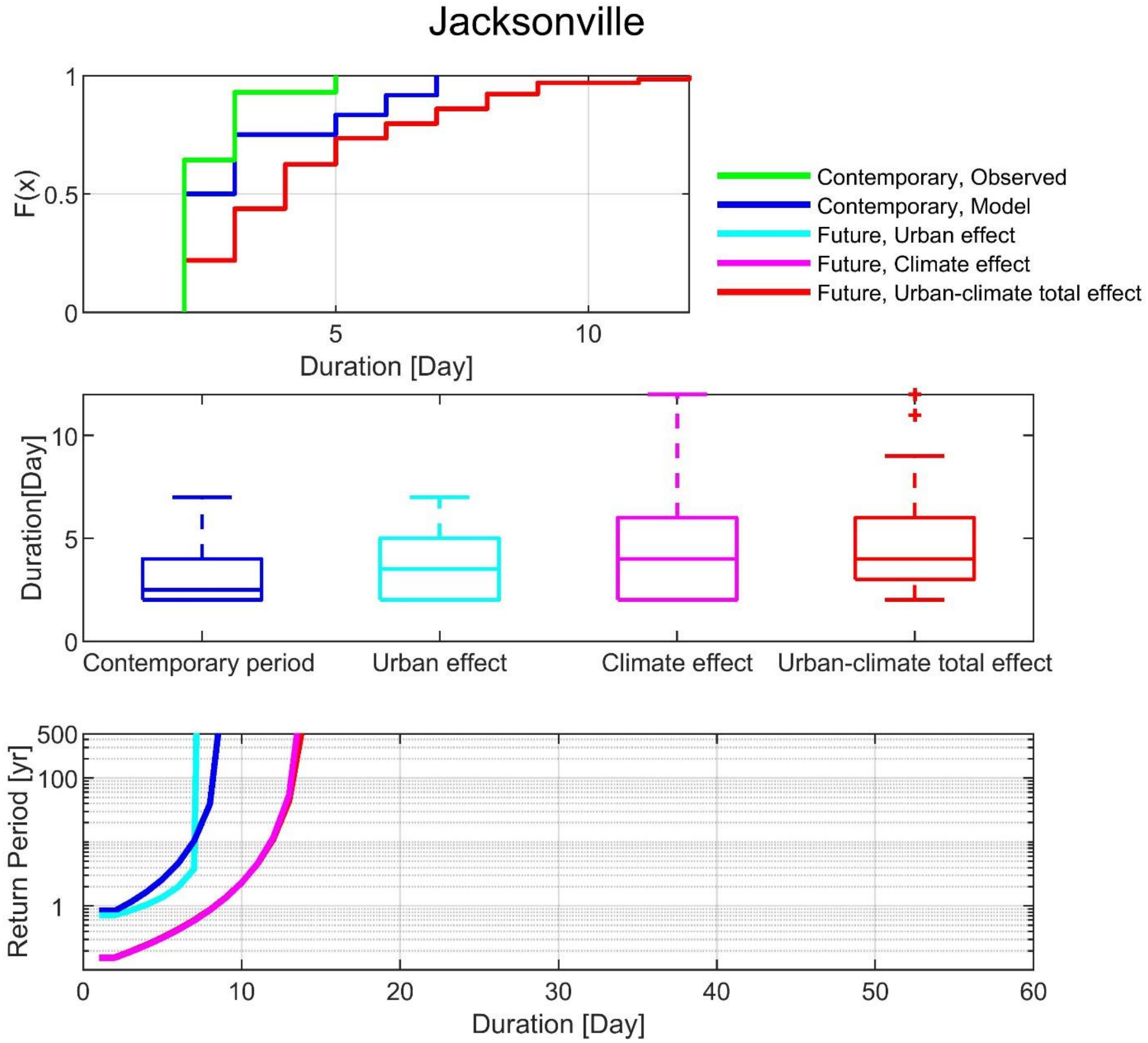

Figure S21. (Top panel) Cumulative distribution function (CDF) of compound dry-hot extreme (CDHE) events based on contemporary observed/model as well as future with consideration of the urban-climate total effect. (Middle panel) Boxplots of future CDHE events with consideration of the urban, climate, and urban-climate total effect. (Bottom panel) Return period return level plot for CDHE events for the contemporary period and future period with consideration of urban, climate, and urban-climate total effect.

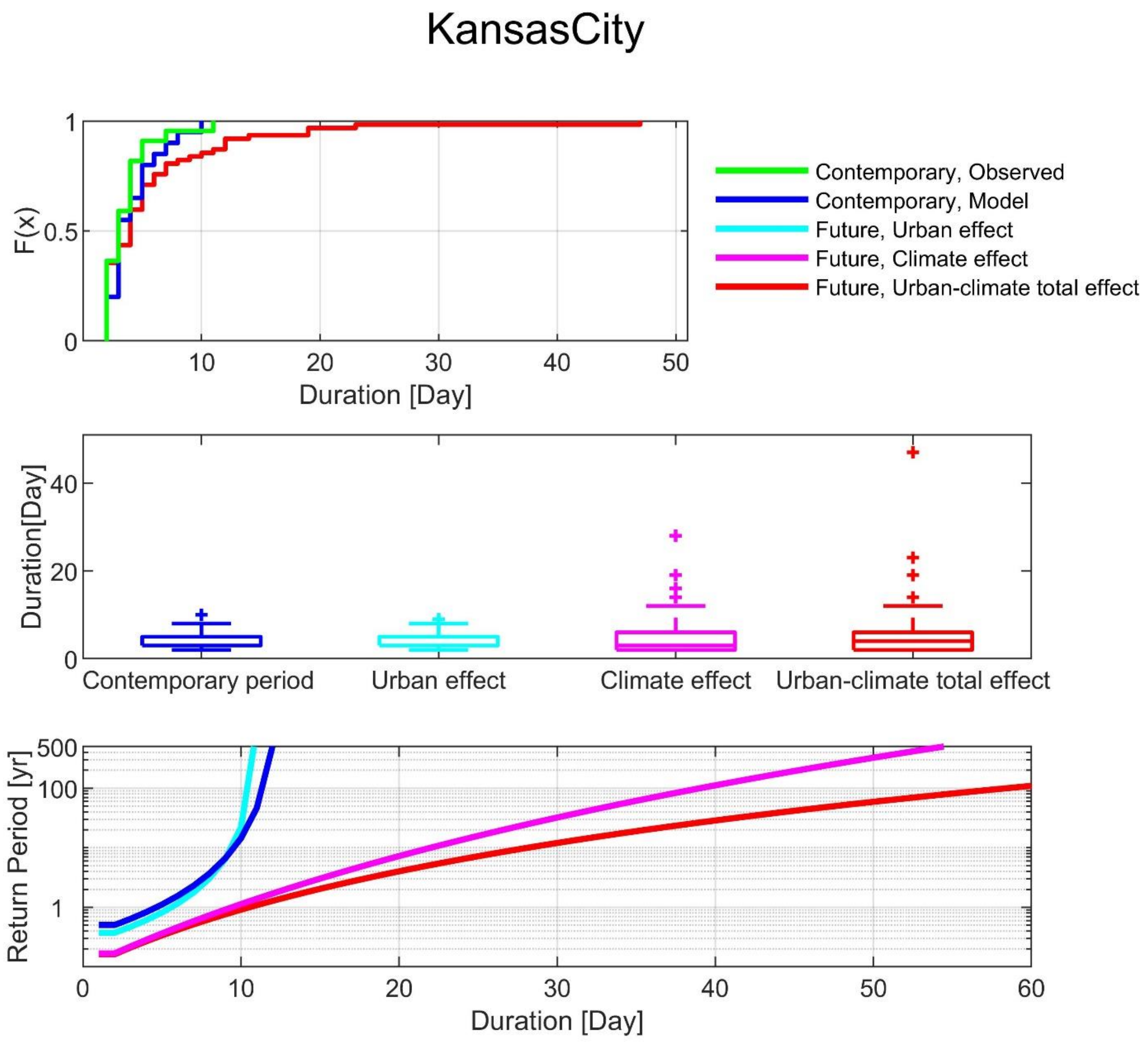

Figure S22. (Top panel) Cumulative distribution function (CDF) of compound dry-hot extreme (CDHE) events based on contemporary observed/model as well as future with consideration of the urban-climate total effect. (Middle panel) Boxplots of future CDHE events with consideration of the urban, climate, and urban-climate total effect. (Bottom panel) Return period return level plot for CDHE events for the contemporary period and future period with consideration of urban, climate, and urban-climate total effect.

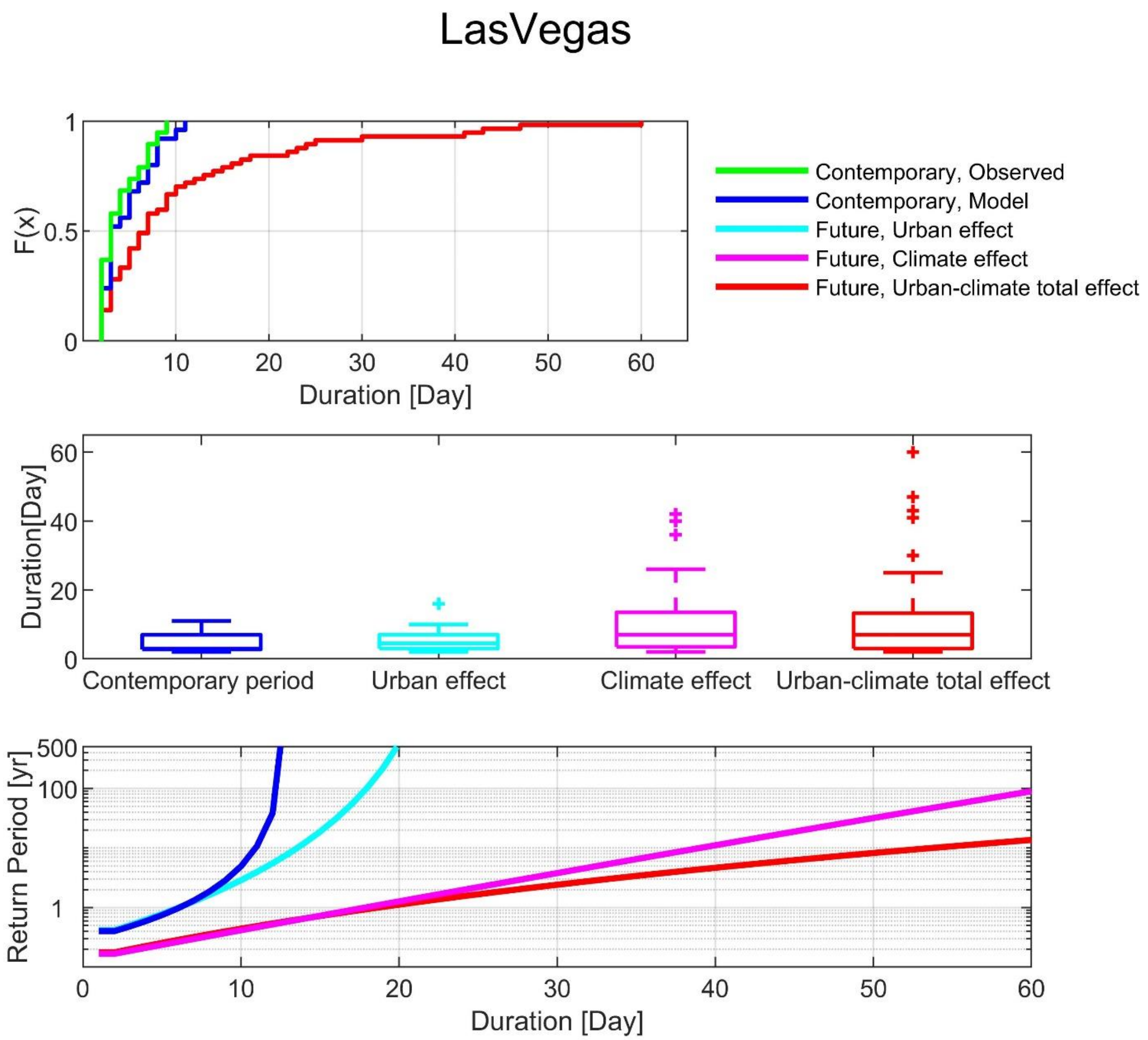

Figure S23. (Top panel) Cumulative distribution function (CDF) of compound dry-hot extreme (CDHE) events based on contemporary observed/model as well as future with consideration of the urban-climate total effect. (Middle panel) Boxplots of future CDHE events with consideration of the urban, climate, and urban-climate total effect. (Bottom panel) Return period return level plot for CDHE events for the contemporary period and future period with consideration of urban, climate, and urban-climate total effect.

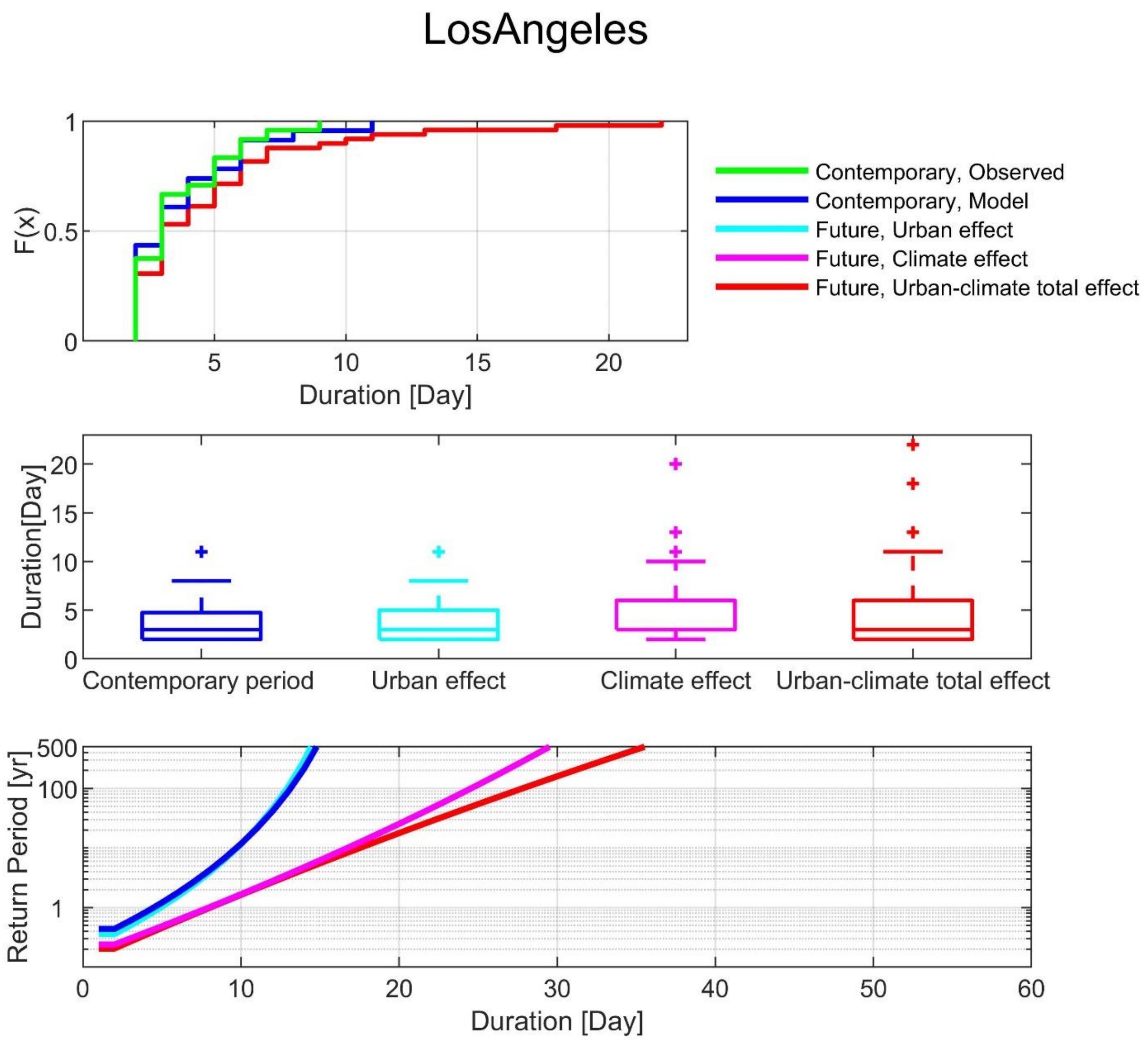

Figure S24. (Top panel) Cumulative distribution function (CDF) of compound dry-hot extreme (CDHE) events based on contemporary observed/model as well as future with consideration of the urban-climate total effect. (Middle panel) Boxplots of future CDHE events with consideration of the urban, climate, and urban-climate total effect. (Bottom panel) Return period return level plot for CDHE events for the contemporary period and future period with consideration of urban, climate, and urban-climate total effect.

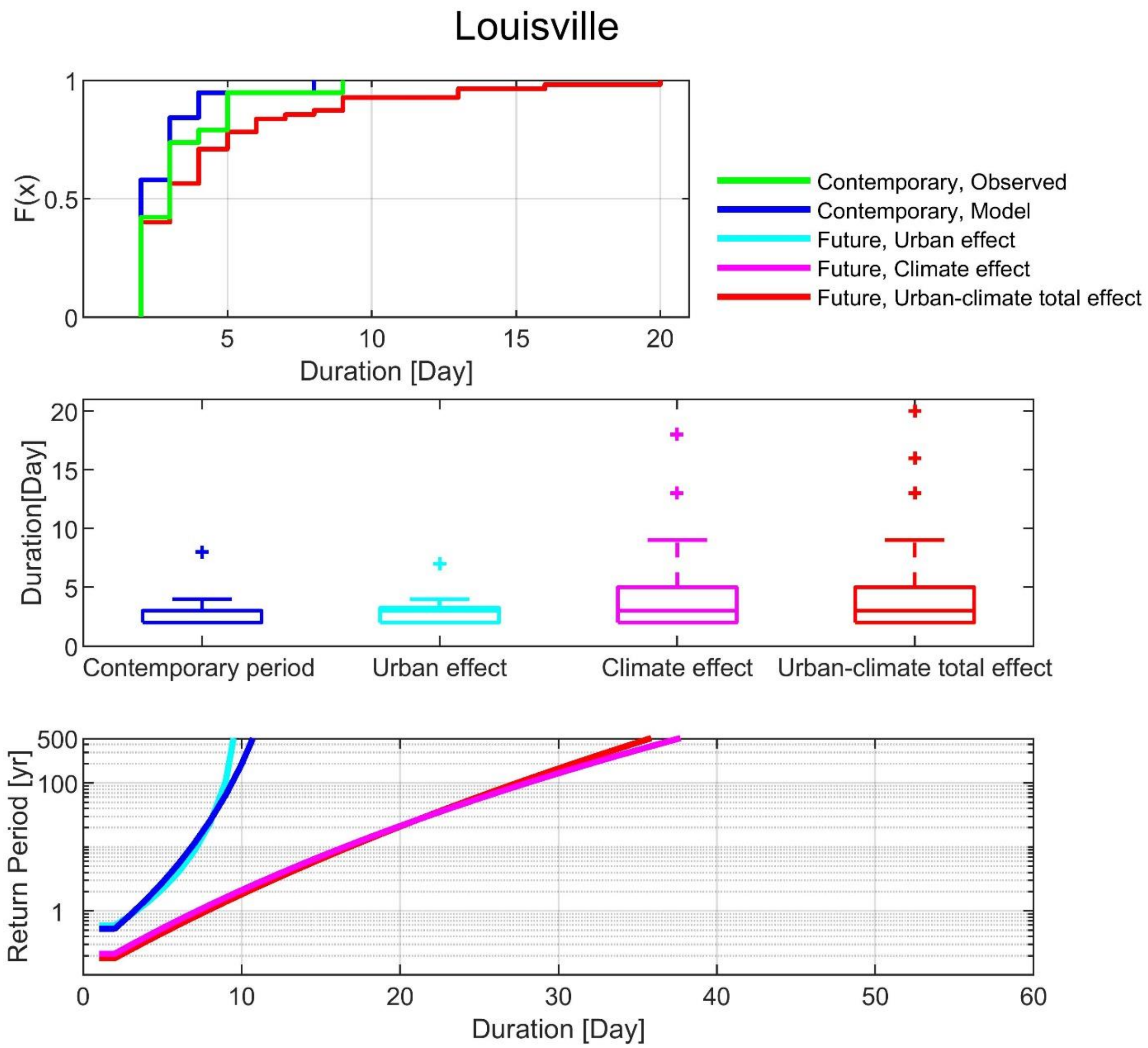

Figure S25. (Top panel) Cumulative distribution function (CDF) of compound dry-hot extreme (CDHE) events based on contemporary observed/model as well as future with consideration of the urban-climate total effect. (Middle panel) Boxplots of future CDHE events with consideration of the urban, climate, and urban-climate total effect. (Bottom panel) Return period return level plot for CDHE events for the contemporary period and future period with consideration of urban, climate, and urban-climate total effect.

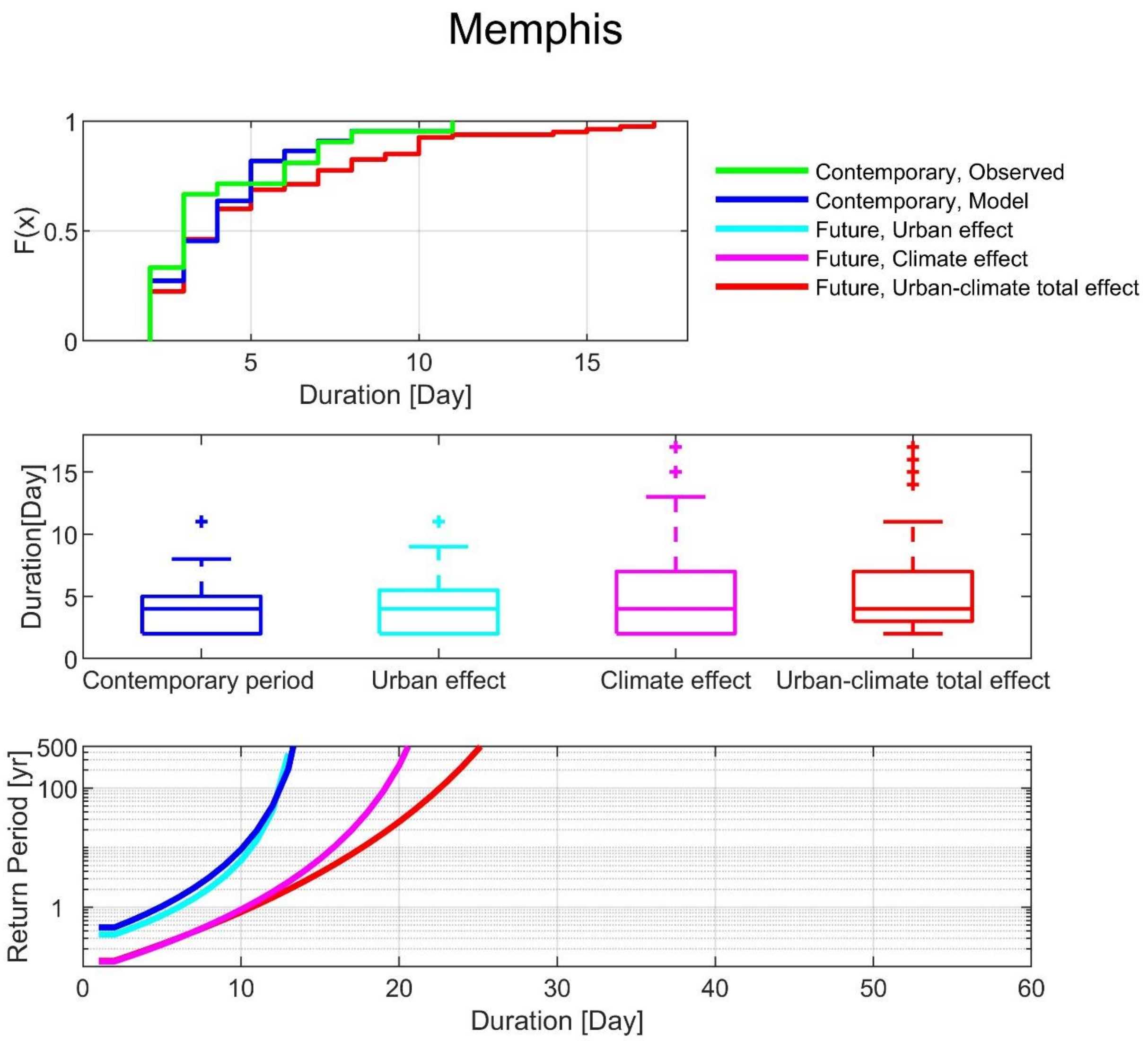

Figure S26. (Top panel) Cumulative distribution function (CDF) of compound dry-hot extreme (CDHE) events based on contemporary observed/model as well as future with consideration of the urban-climate total effect. (Middle panel) Boxplots of future CDHE events with consideration of the urban, climate, and urban-climate total effect. (Bottom panel) Return period return level plot for CDHE events for the contemporary period and future period with consideration of urban, climate, and urban-climate total effect.

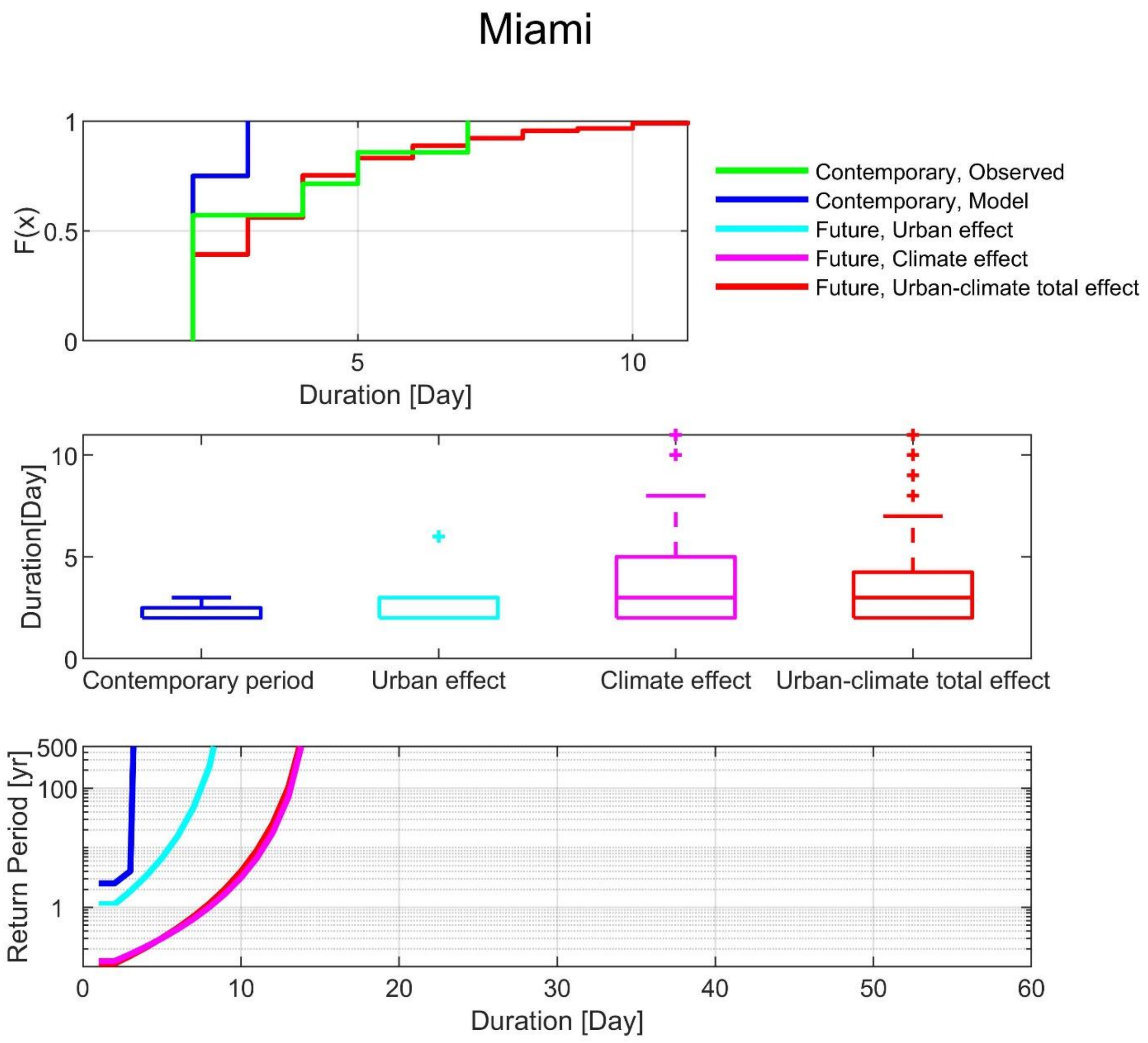

Figure S27. (Top panel) Cumulative distribution function (CDF) of compound dry-hot extreme (CDHE) events based on contemporary observed/model as well as future with consideration of the urban-climate total effect. (Middle panel) Boxplots of future CDHE events with consideration of the urban, climate, and urban-climate total effect. (Bottom panel) Return period return level plot for CDHE events for the contemporary period and future period with consideration of urban, climate, and urban-climate total effect.

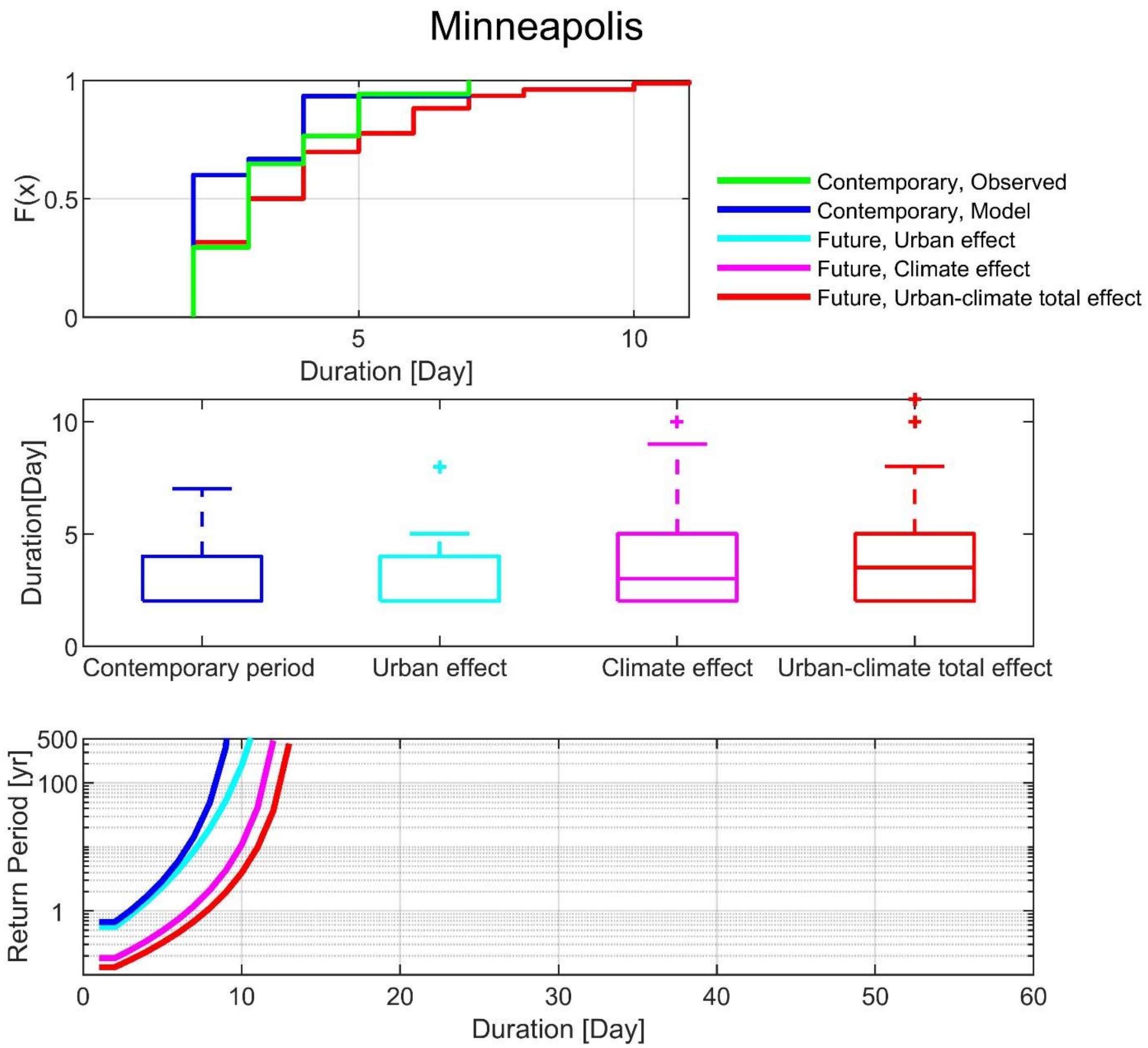

Figure S28. (Top panel) Cumulative distribution function (CDF) of compound dry-hot extreme (CDHE) events based on contemporary observed/model as well as future with consideration of the urban-climate total effect. (Middle panel) Boxplots of future CDHE events with consideration of the urban, climate, and urban-climate total effect. (Bottom panel) Return period return level plot for CDHE events for the contemporary period and future period with consideration of urban, climate, and urban-climate total effect.

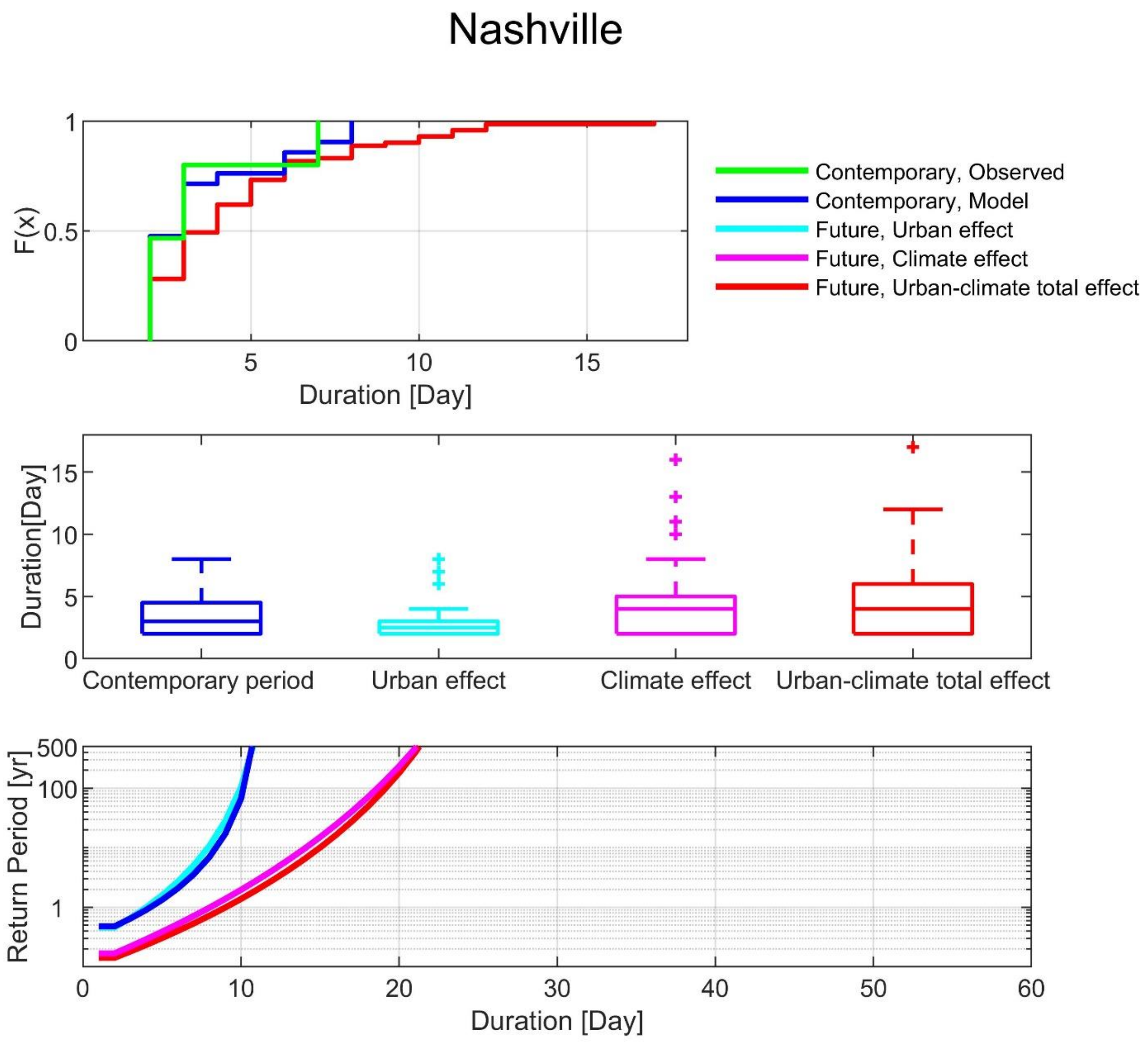

Figure S29. (Top panel) Cumulative distribution function (CDF) of compound dry-hot extreme (CDHE) events based on contemporary observed/model as well as future with consideration of the urban-climate total effect. (Middle panel) Boxplots of future CDHE events with consideration of the urban, climate, and urban-climate total effect. (Bottom panel) Return period return level plot for CDHE events for the contemporary period and future period with consideration of urban, climate, and urban-climate total effect.

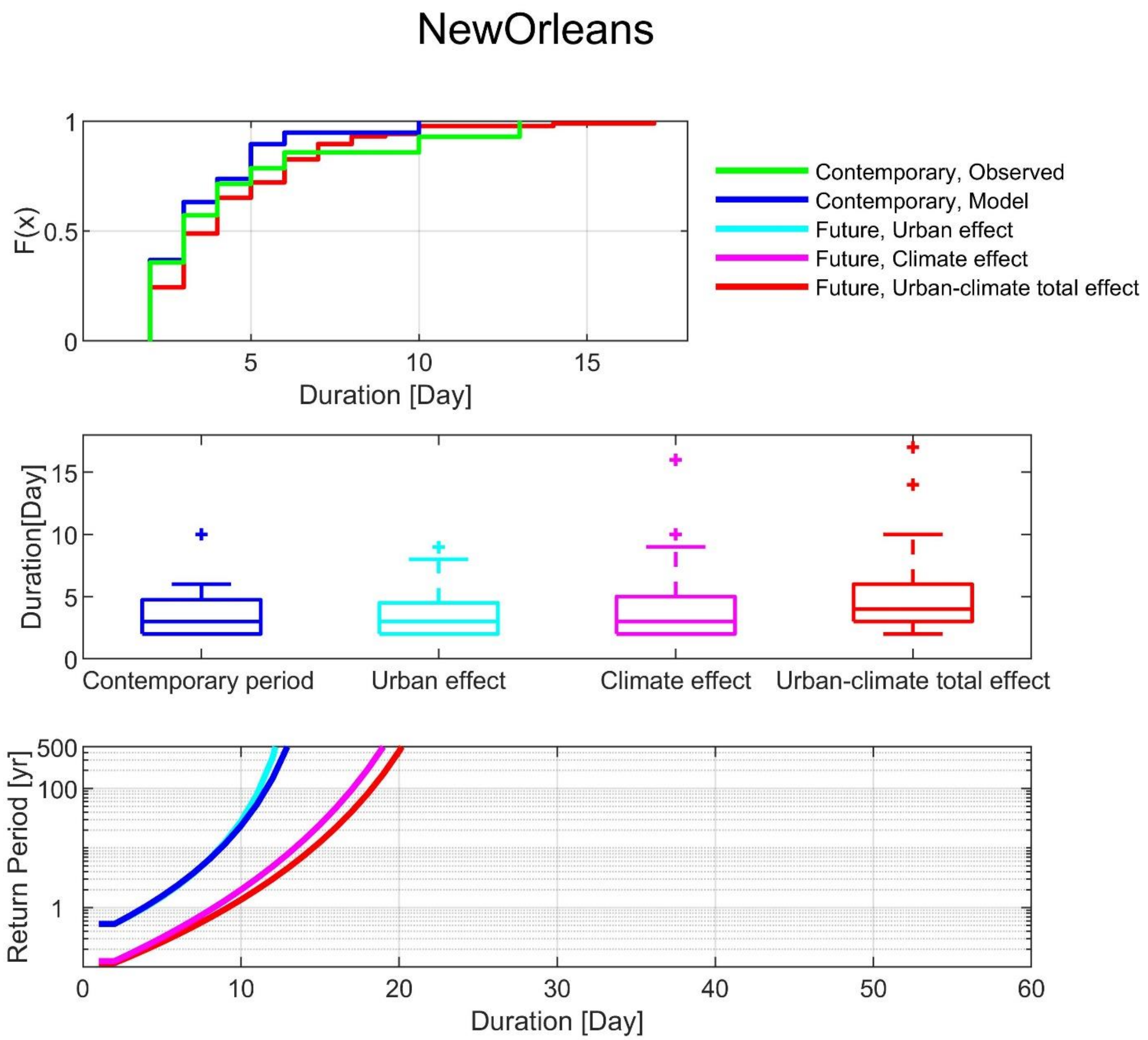

Figure S30. (Top panel) Cumulative distribution function (CDF) of compound dry-hot extreme (CDHE) events based on contemporary observed/model as well as future with consideration of the urban-climate total effect. (Middle panel) Boxplots of future CDHE events with consideration of the urban, climate, and urban-climate total effect. (Bottom panel) Return period return level plot for CDHE events for the contemporary period and future period with consideration of urban, climate, and urban-climate total effect.

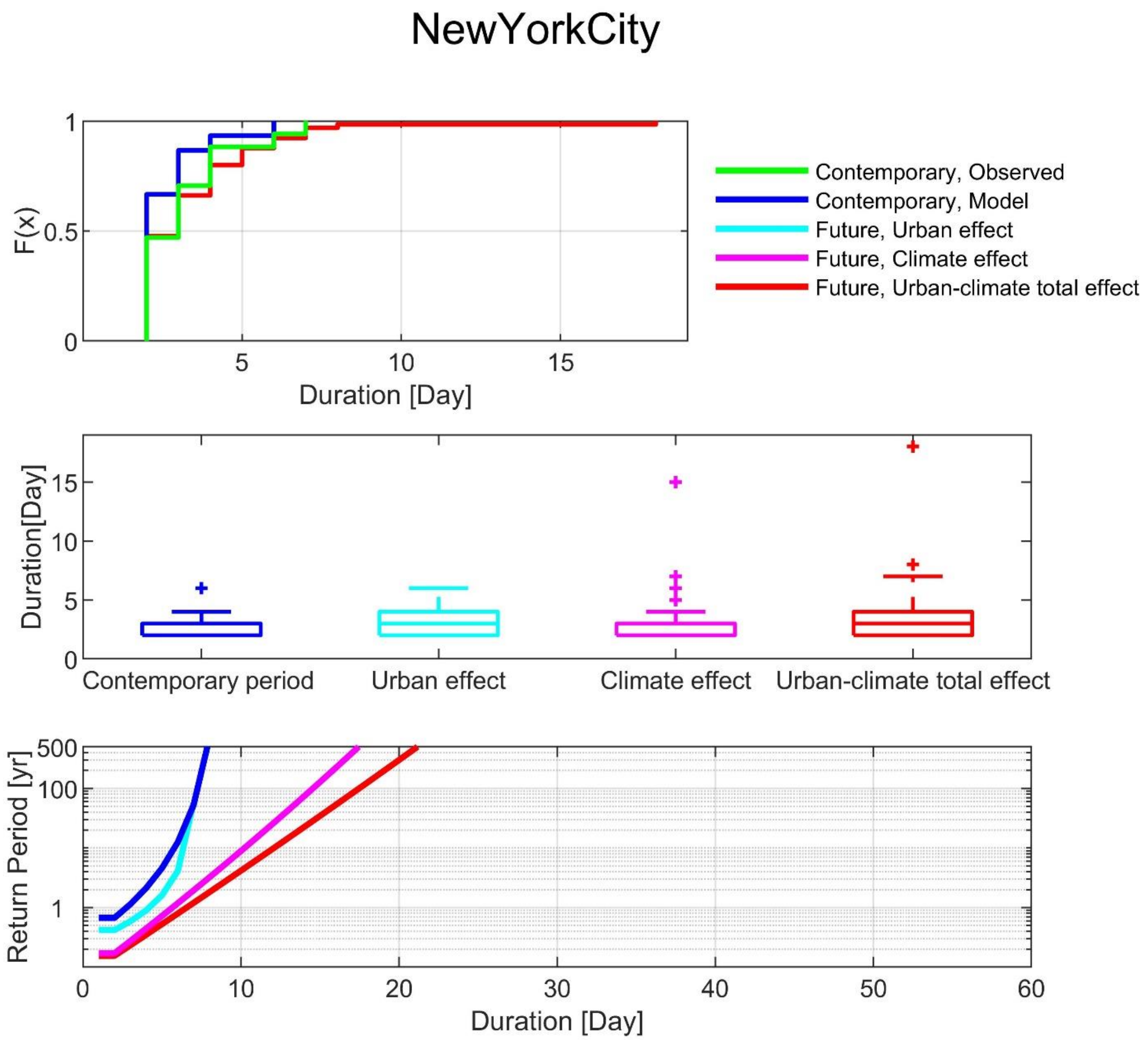

Figure S31. (Top panel) Cumulative distribution function (CDF) of compound dry-hot extreme (CDHE) events based on contemporary observed/model as well as future with consideration of the urban-climate total effect. (Middle panel) Boxplots of future CDHE events with consideration of the urban, climate, and urban-climate total effect. (Bottom panel) Return period return level plot for CDHE events for the contemporary period and future period with consideration of urban, climate, and urban-climate total effect.

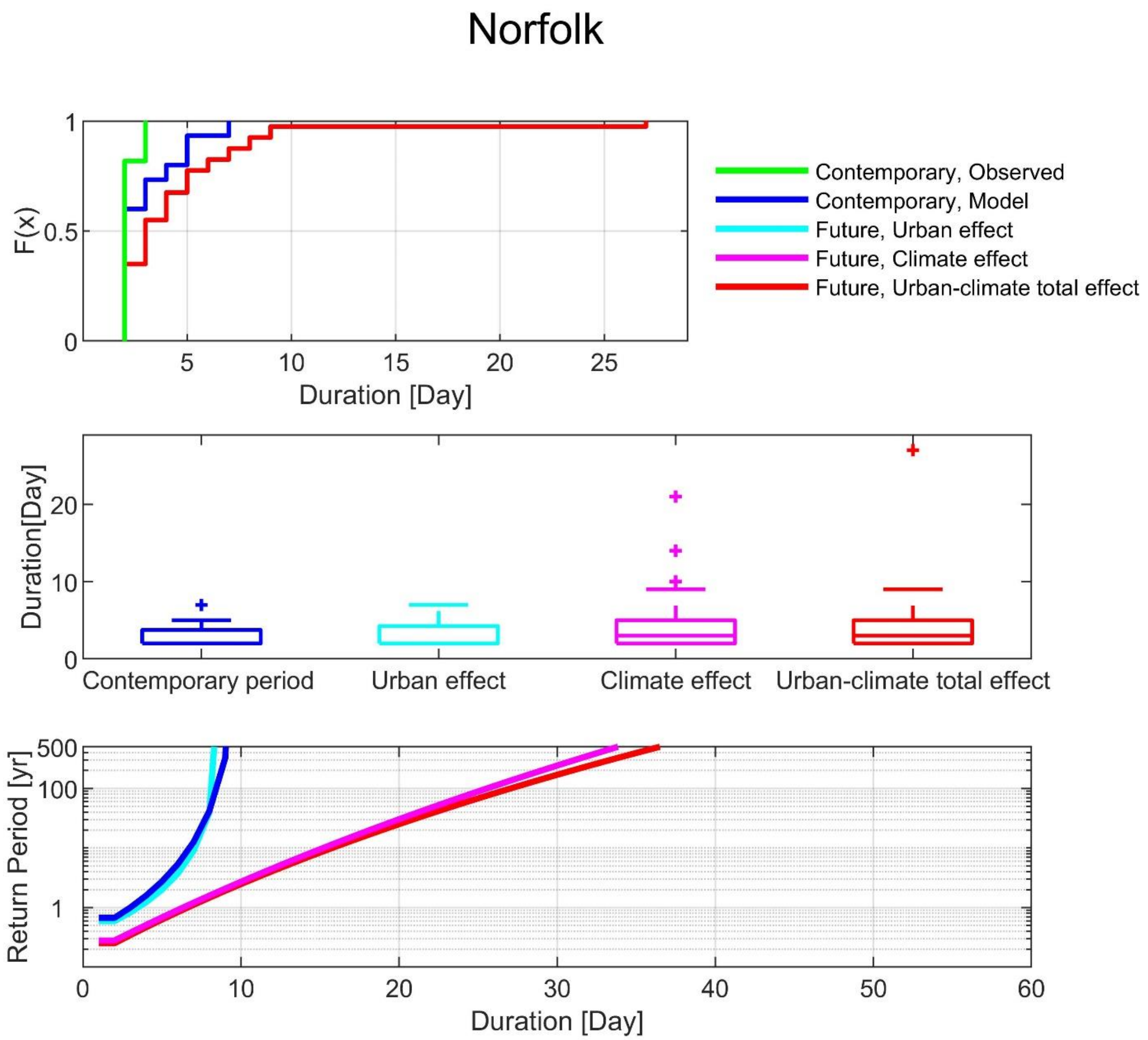

Figure S32. (Top panel) Cumulative distribution function (CDF) of compound dry-hot extreme (CDHE) events based on contemporary observed/model as well as future with consideration of the urban-climate total effect. (Middle panel) Boxplots of future CDHE events with consideration of the urban, climate, and urban-climate total effect. (Bottom panel) Return period return level plot for CDHE events for the contemporary period and future period with consideration of urban, climate, and urban-climate total effect.

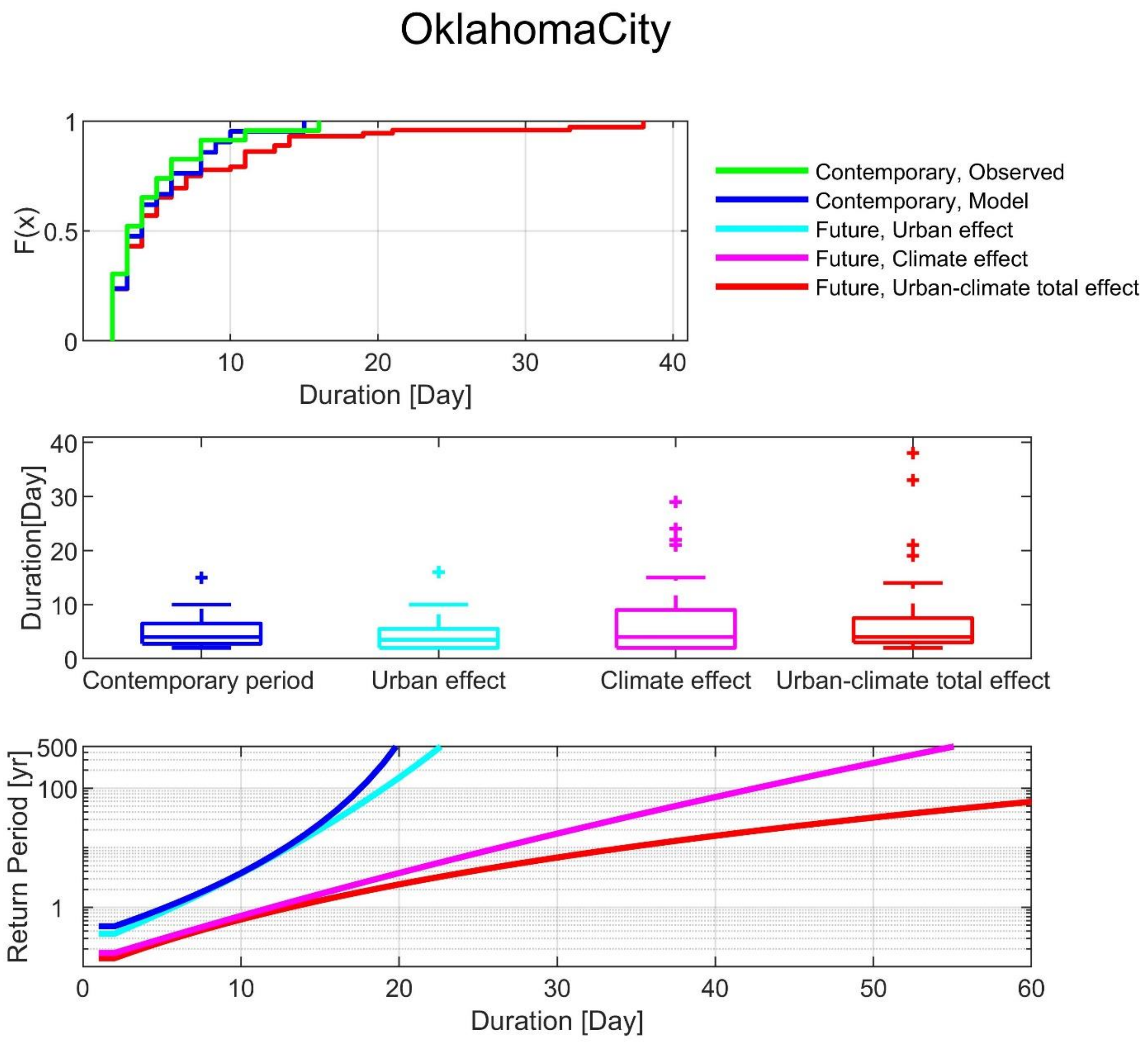

Figure S33. (Top panel) Cumulative distribution function (CDF) of compound dry-hot extreme (CDHE) events based on contemporary observed/model as well as future with consideration of the urban-climate total effect. (Middle panel) Boxplots of future CDHE events with consideration of the urban, climate, and urban-climate total effect. (Bottom panel) Return period return level plot for CDHE events for the contemporary period and future period with consideration of urban, climate, and urban-climate total effect.

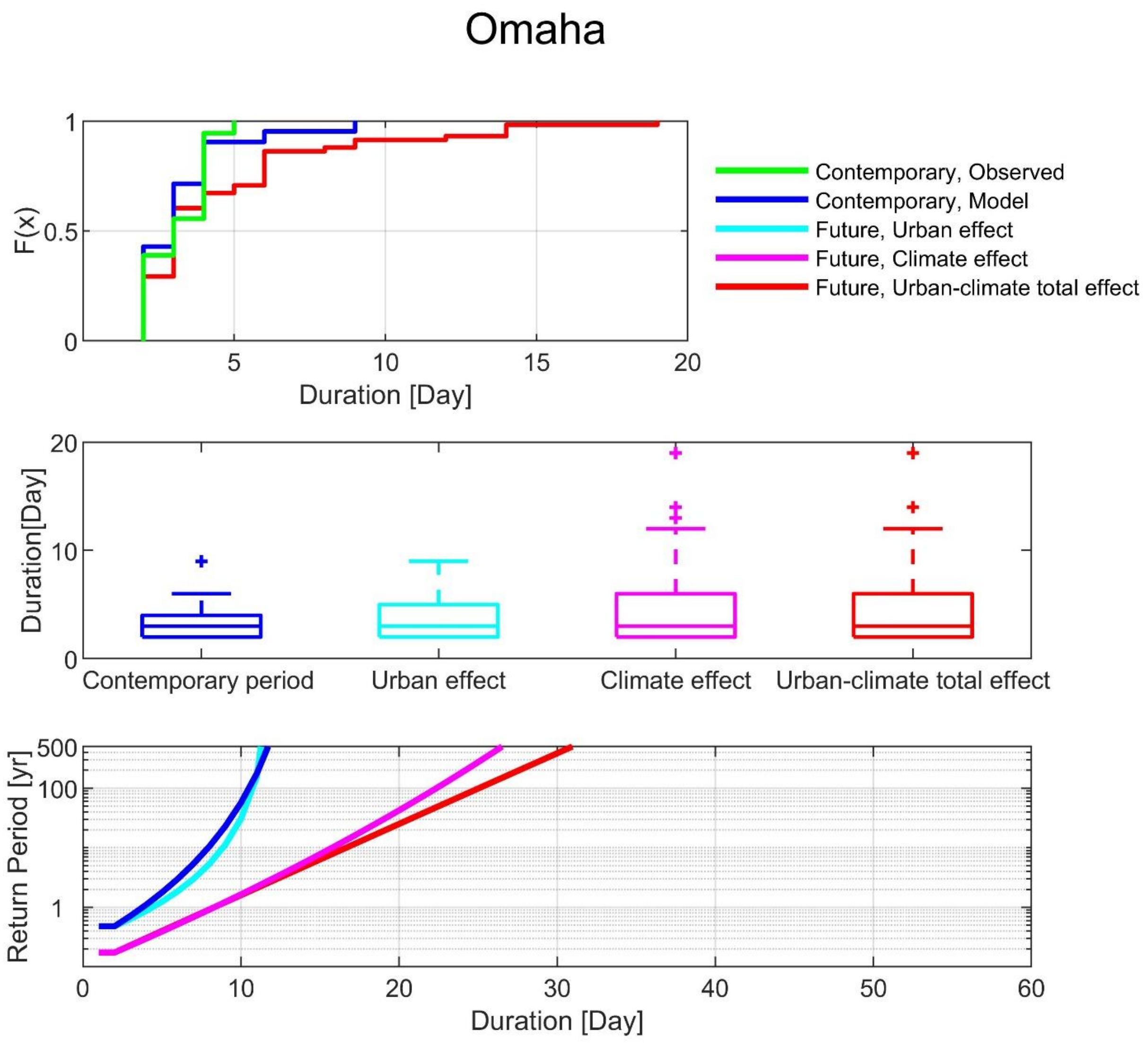

Figure S34. (Top panel) Cumulative distribution function (CDF) of compound dry-hot extreme (CDHE) events based on contemporary observed/model as well as future with consideration of the urban-climate total effect. (Middle panel) Boxplots of future CDHE events with consideration of the urban, climate, and urban-climate total effect. (Bottom panel) Return period return level plot for CDHE events for the contemporary period and future period with consideration of urban, climate, and urban-climate total effect.

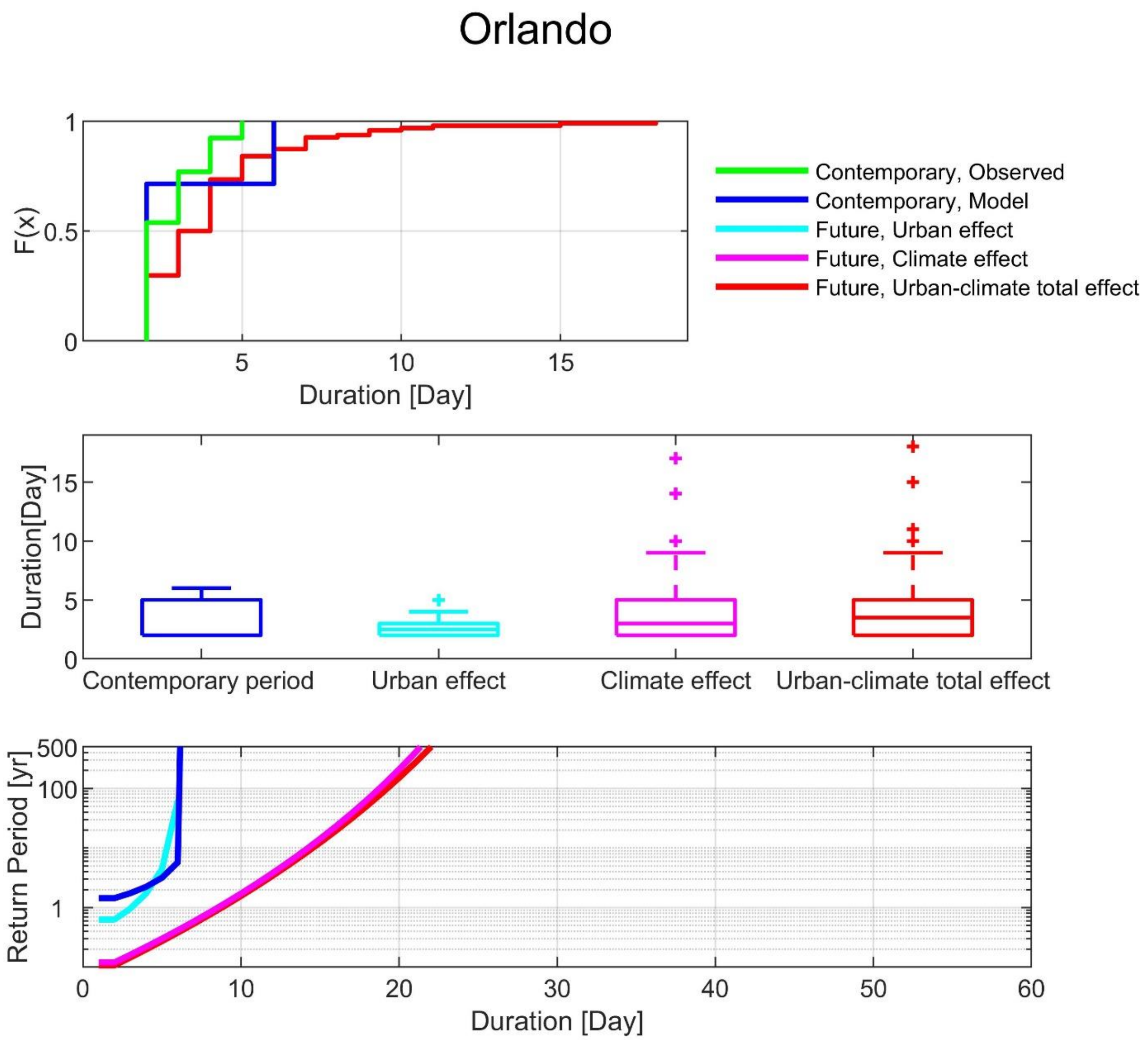

Figure S35. (Top panel) Cumulative distribution function (CDF) of compound dry-hot extreme (CDHE) events based on contemporary observed/model as well as future with consideration of the urban-climate total effect. (Middle panel) Boxplots of future CDHE events with consideration of the urban, climate, and urban-climate total effect. (Bottom panel) Return period return level plot for CDHE events for the contemporary period and future period with consideration of urban, climate, and urban-climate total effect.

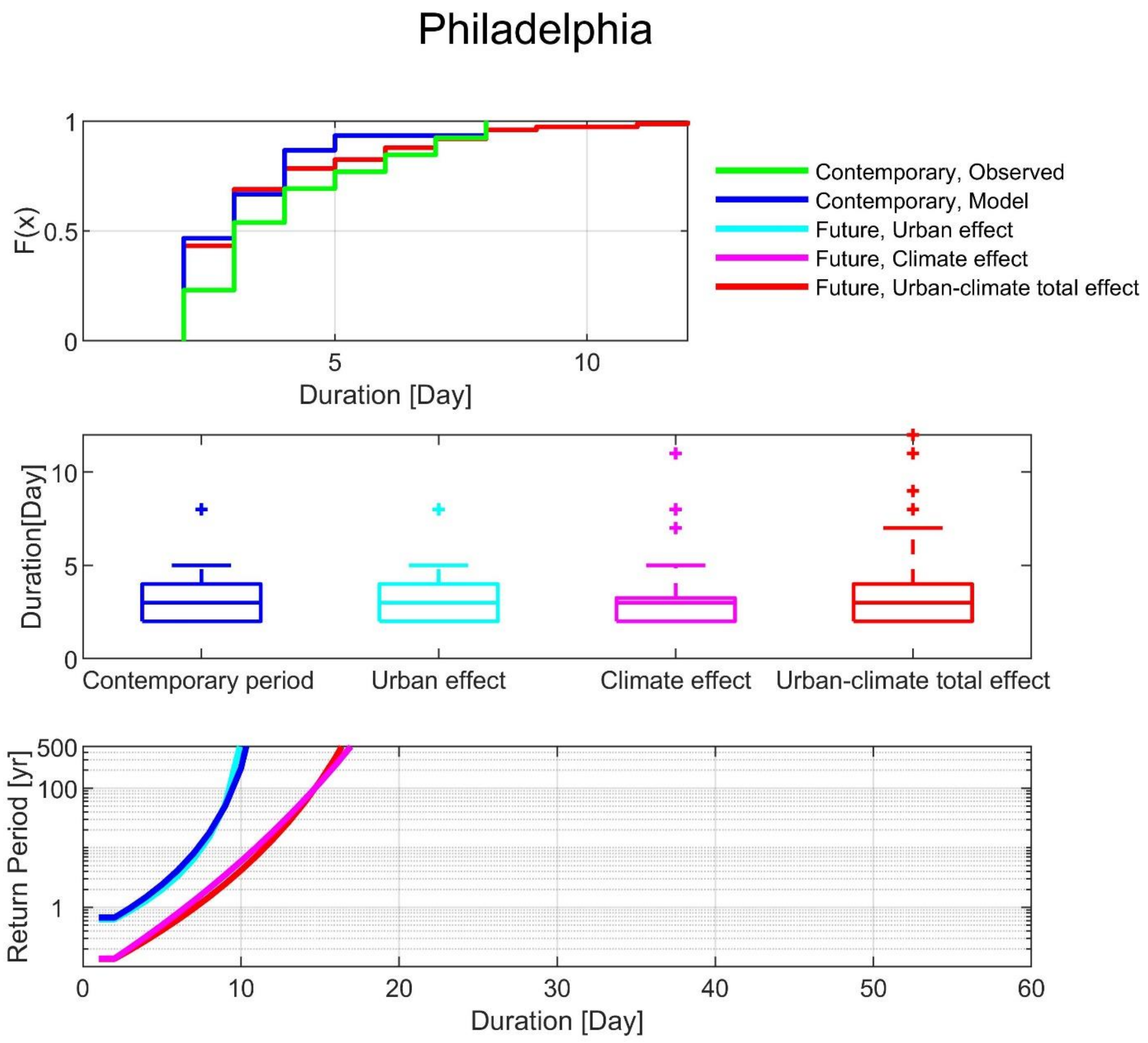

Figure S36. (Top panel) Cumulative distribution function (CDF) of compound dry-hot extreme (CDHE) events based on contemporary observed/model as well as future with consideration of the urban-climate total effect. (Middle panel) Boxplots of future CDHE events with consideration of the urban, climate, and urban-climate total effect. (Bottom panel) Return period return level plot for CDHE events for the contemporary period and future period with consideration of urban, climate, and urban-climate total effect.

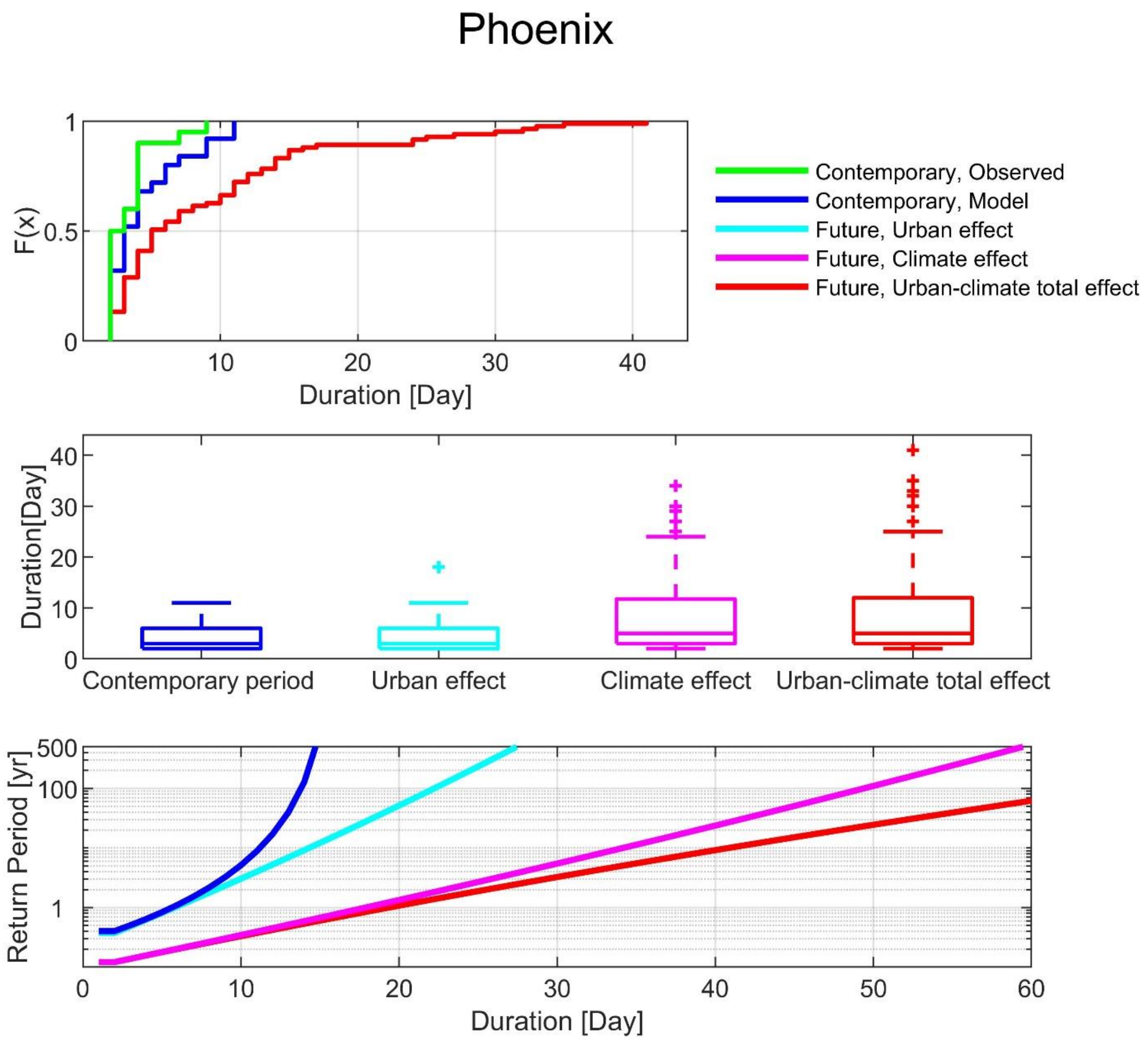

Figure S37. (Top panel) Cumulative distribution function (CDF) of compound dry-hot extreme (CDHE) events based on contemporary observed/model as well as future with consideration of the urban-climate total effect. (Middle panel) Boxplots of future CDHE events with consideration of the urban, climate, and urban-climate total effect. (Bottom panel) Return period return level plot for CDHE events for the contemporary period and future period with consideration of urban, climate, and urban-climate total effect.

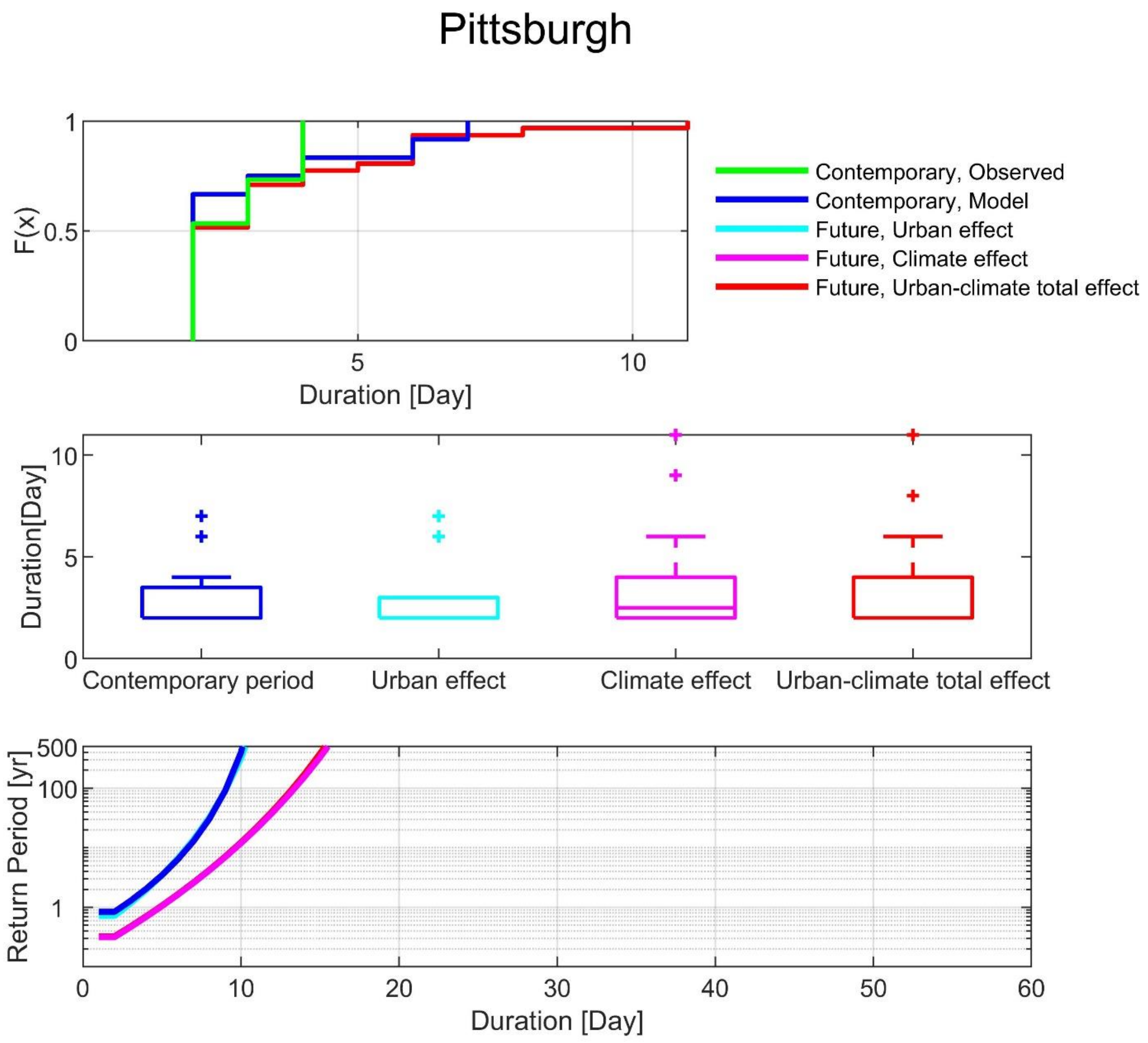

Figure S38. (Top panel) Cumulative distribution function (CDF) of compound dry-hot extreme (CDHE) events based on contemporary observed/model as well as future with consideration of the urban-climate total effect. (Middle panel) Boxplots of future CDHE events with consideration of the urban, climate, and urban-climate total effect. (Bottom panel) Return period return level plot for CDHE events for the contemporary period and future period with consideration of urban, climate, and urban-climate total effect.

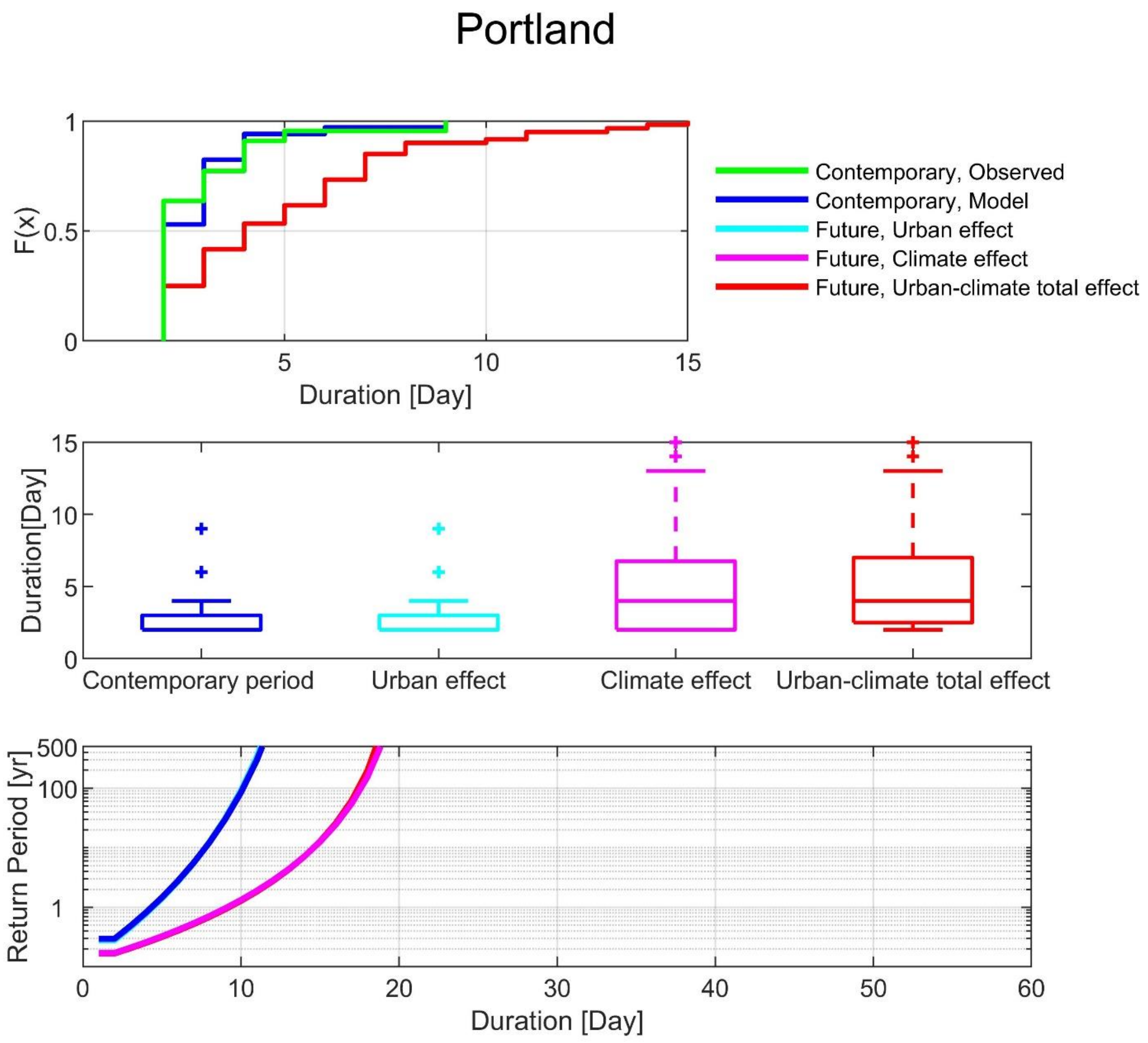

Figure S39. (Top panel) Cumulative distribution function (CDF) of compound dry-hot extreme (CDHE) events based on contemporary observed/model as well as future with consideration of the urban-climate total effect. (Middle panel) Boxplots of future CDHE events with consideration of the urban, climate, and urban-climate total effect. (Bottom panel) Return period return level plot for CDHE events for the contemporary period and future period with consideration of urban, climate, and urban-climate total effect.

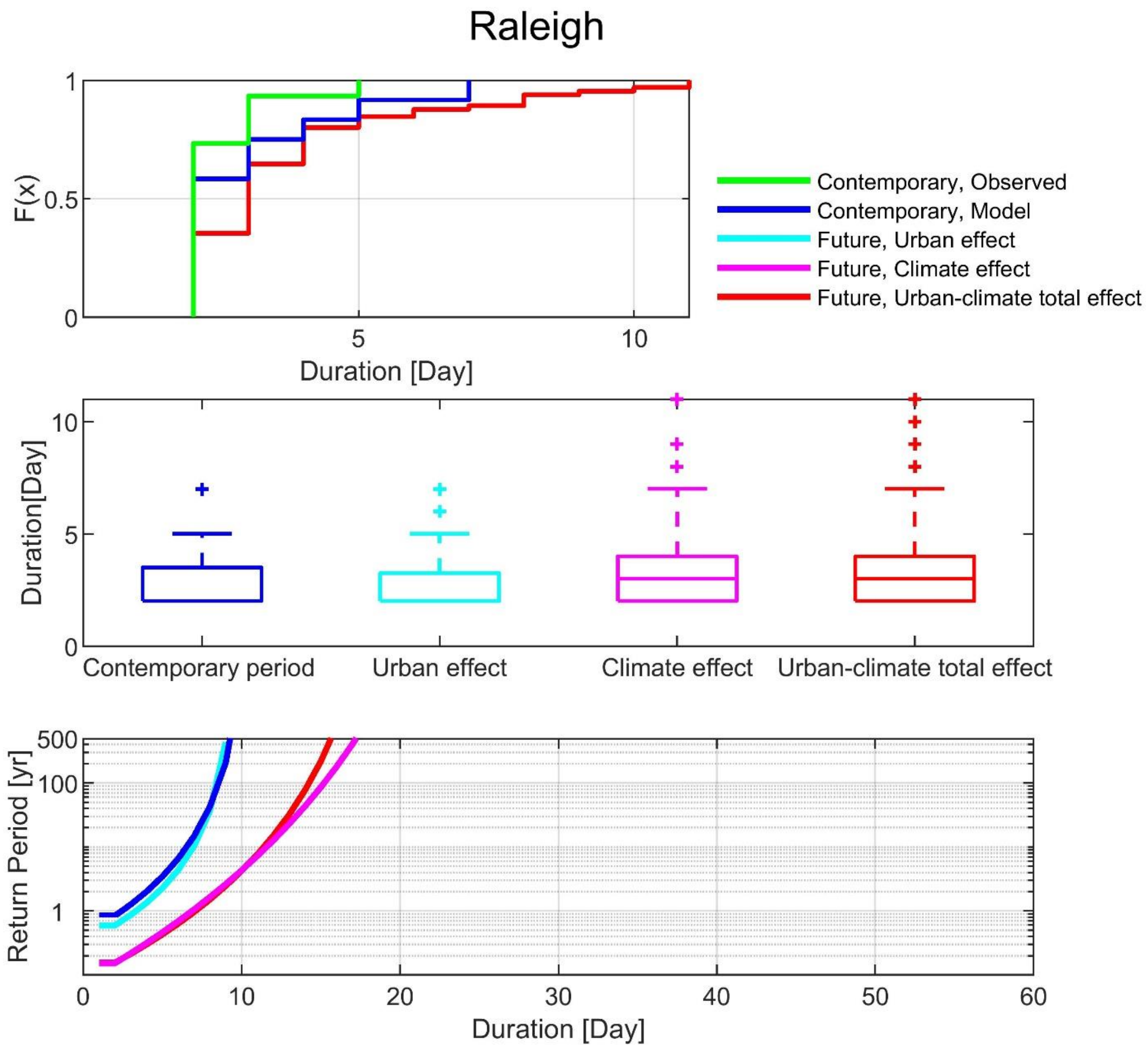

Figure S40. (Top panel) Cumulative distribution function (CDF) of compound dry-hot extreme (CDHE) events based on contemporary observed/model as well as future with consideration of the urban-climate total effect. (Middle panel) Boxplots of future CDHE events with consideration of the urban, climate, and urban-climate total effect. (Bottom panel) Return period return level plot for CDHE events for the contemporary period and future period with consideration of urban, climate, and urban-climate total effect.

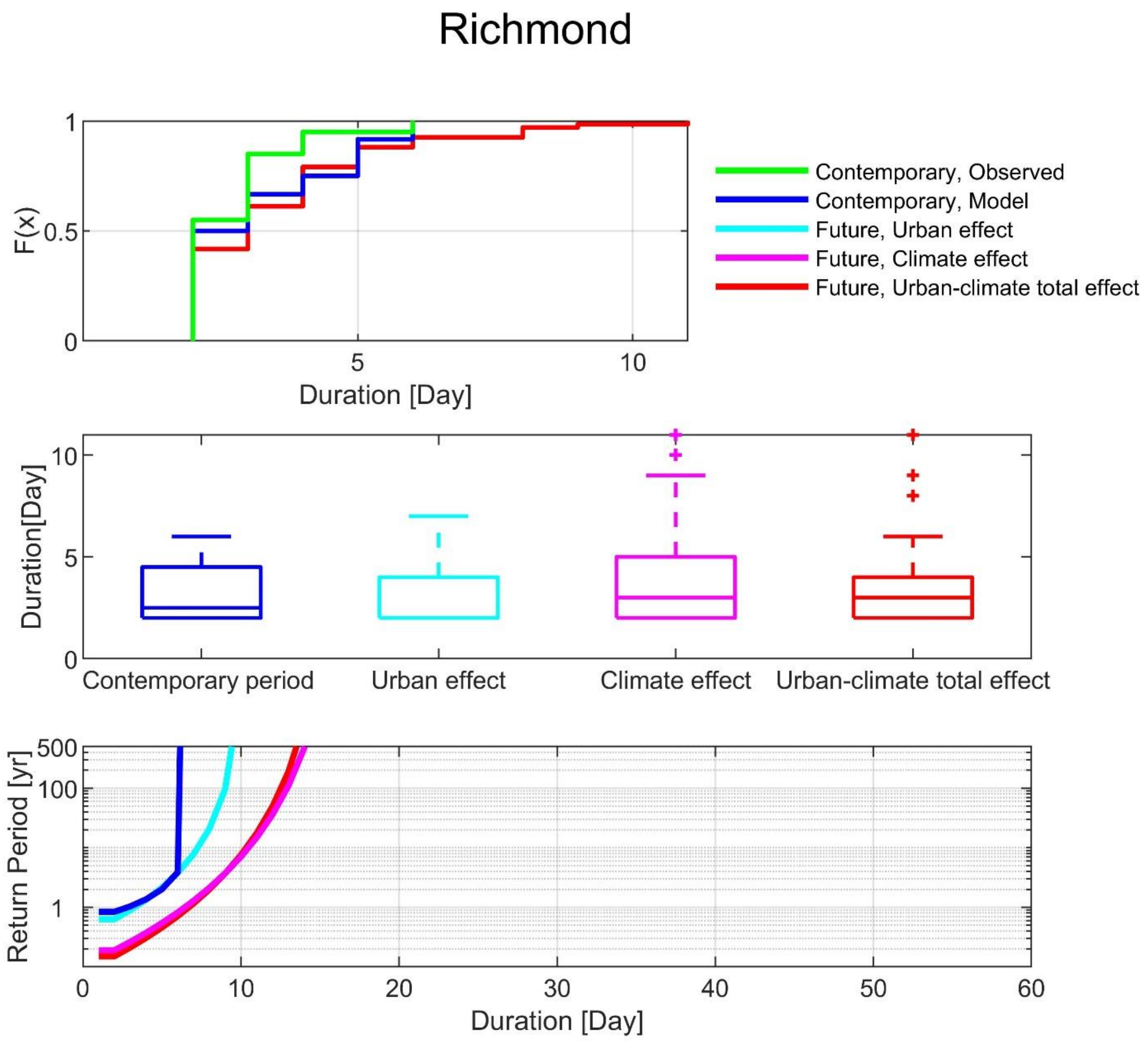

Figure S41. (Top panel) Cumulative distribution function (CDF) of compound dry-hot extreme (CDHE) events based on contemporary observed/model as well as future with consideration of the urban-climate total effect. (Middle panel) Boxplots of future CDHE events with consideration of the urban, climate, and urban-climate total effect. (Bottom panel) Return period return level plot for CDHE events for the contemporary period and future period with consideration of urban, climate, and urban-climate total effect.

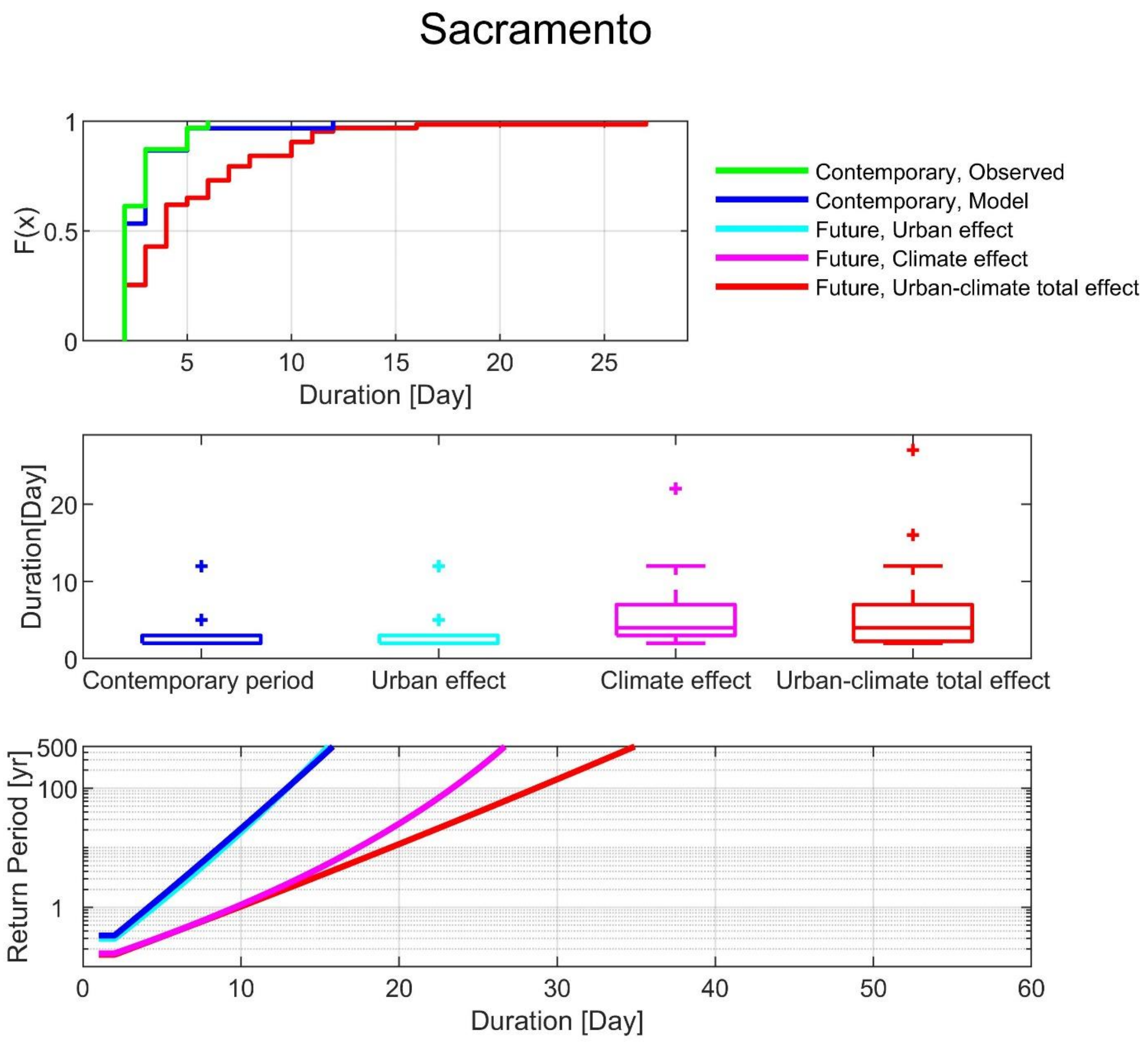

Figure S42. (Top panel) Cumulative distribution function (CDF) of compound dry-hot extreme (CDHE) events based on contemporary observed/model as well as future with consideration of the urban-climate total effect. (Middle panel) Boxplots of future CDHE events with consideration of the urban, climate, and urban-climate total effect. (Bottom panel) Return period return level plot for CDHE events for the contemporary period and future period with consideration of urban, climate, and urban-climate total effect.

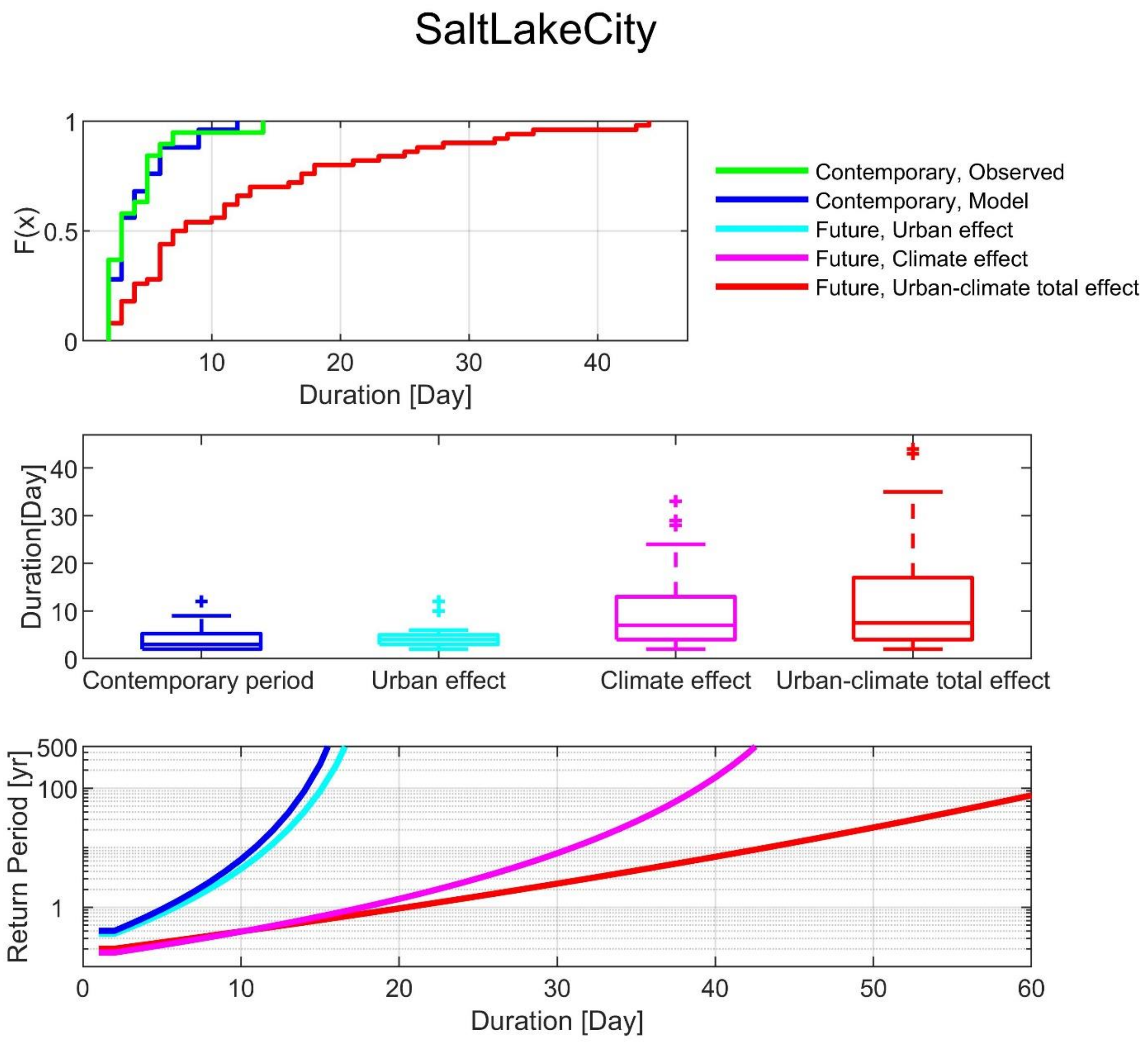

Figure S43. (Top panel) Cumulative distribution function (CDF) of compound dry-hot extreme (CDHE) events based on contemporary observed/model as well as future with consideration of the urban-climate total effect. (Middle panel) Boxplots of future CDHE events with consideration of the urban, climate, and urban-climate total effect. (Bottom panel) Return period return level plot for CDHE events for the contemporary period and future period with consideration of urban, climate, and urban-climate total effect.

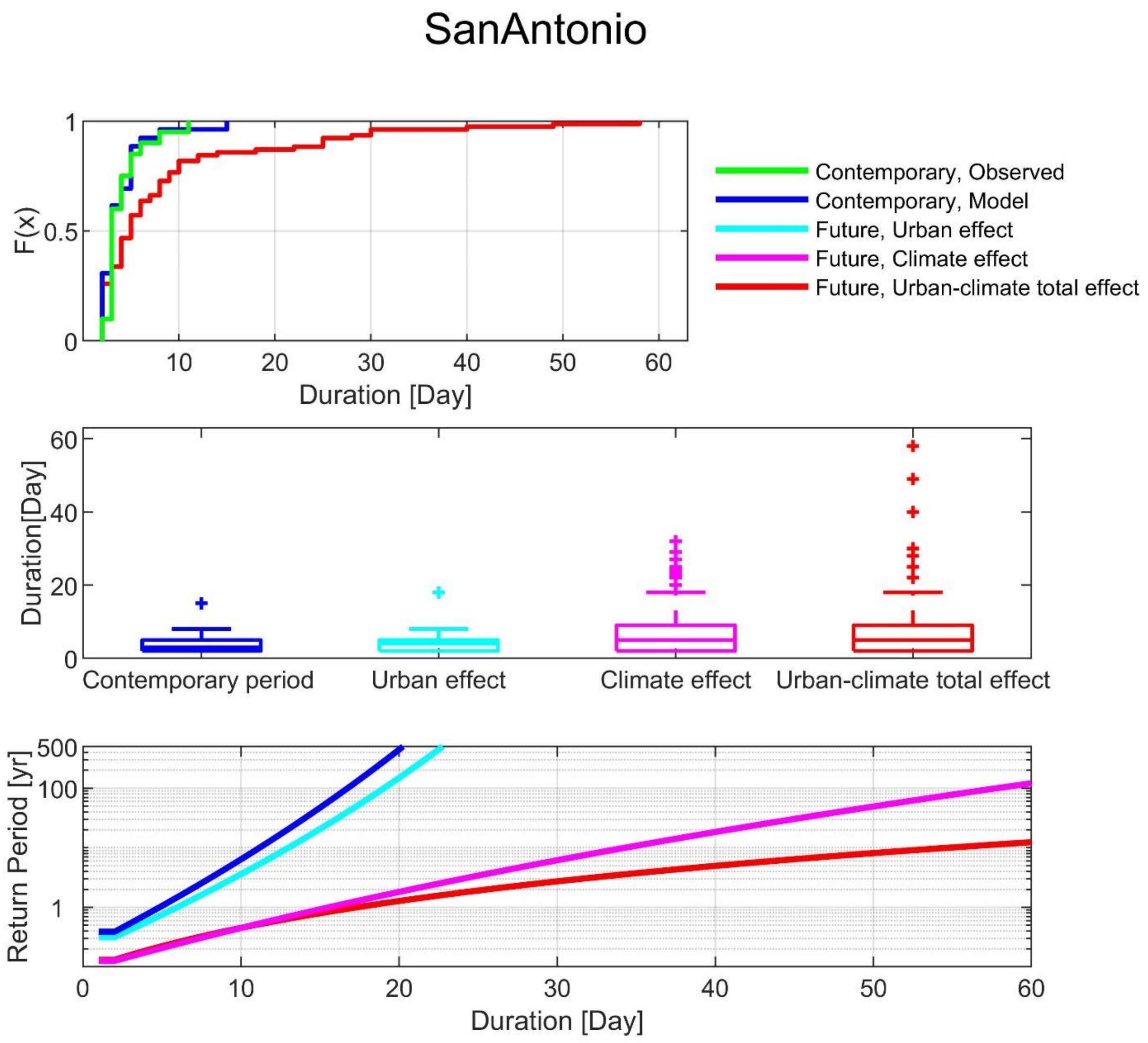

Figure S44. (Top panel) Cumulative distribution function (CDF) of compound dry-hot extreme (CDHE) events based on contemporary observed/model as well as future with consideration of the urban-climate total effect. (Middle panel) Boxplots of future CDHE events with consideration of the urban, climate, and urban-climate total effect. (Bottom panel) Return period return level plot for CDHE events for the contemporary period and future period with consideration of urban, climate, and urban-climate total effect.

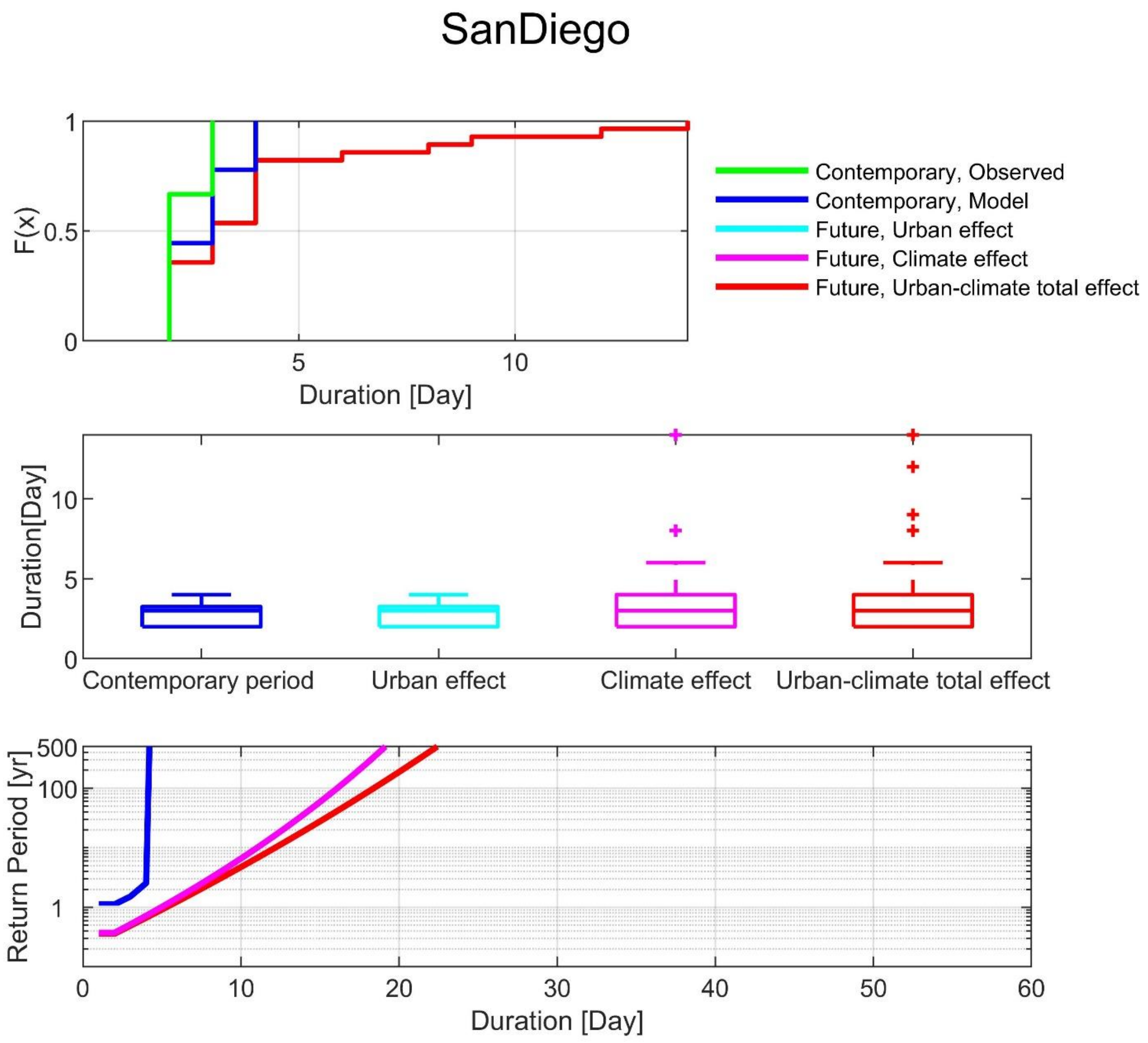

Figure S45. (Top panel) Cumulative distribution function (CDF) of compound dry-hot extreme (CDHE) events based on contemporary observed/model as well as future with consideration of the urban-climate total effect. (Middle panel) Boxplots of future CDHE events with consideration of the urban, climate, and urban-climate total effect. (Bottom panel) Return period return level plot for CDHE events for the contemporary period and future period with consideration of urban, climate, and urban-climate total effect.

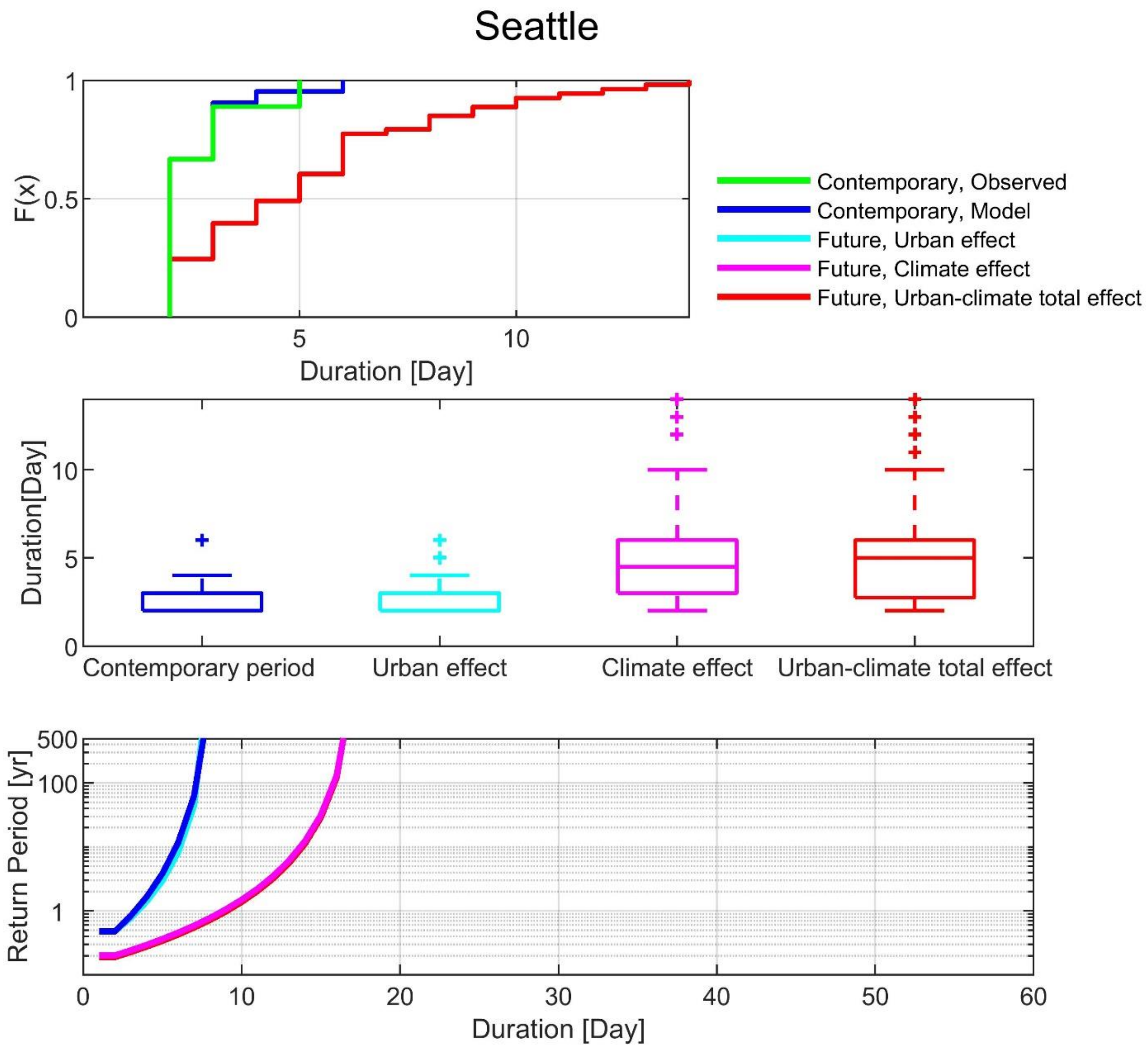

Figure S46. (Top panel) Cumulative distribution function (CDF) of compound dry-hot extreme (CDHE) events based on contemporary observed/model as well as future with consideration of the urban-climate total effect. (Middle panel) Boxplots of future CDHE events with consideration of the urban, climate, and urban-climate total effect. (Bottom panel) Return period return level plot for CDHE events for the contemporary period and future period with consideration of urban, climate, and urban-climate total effect.

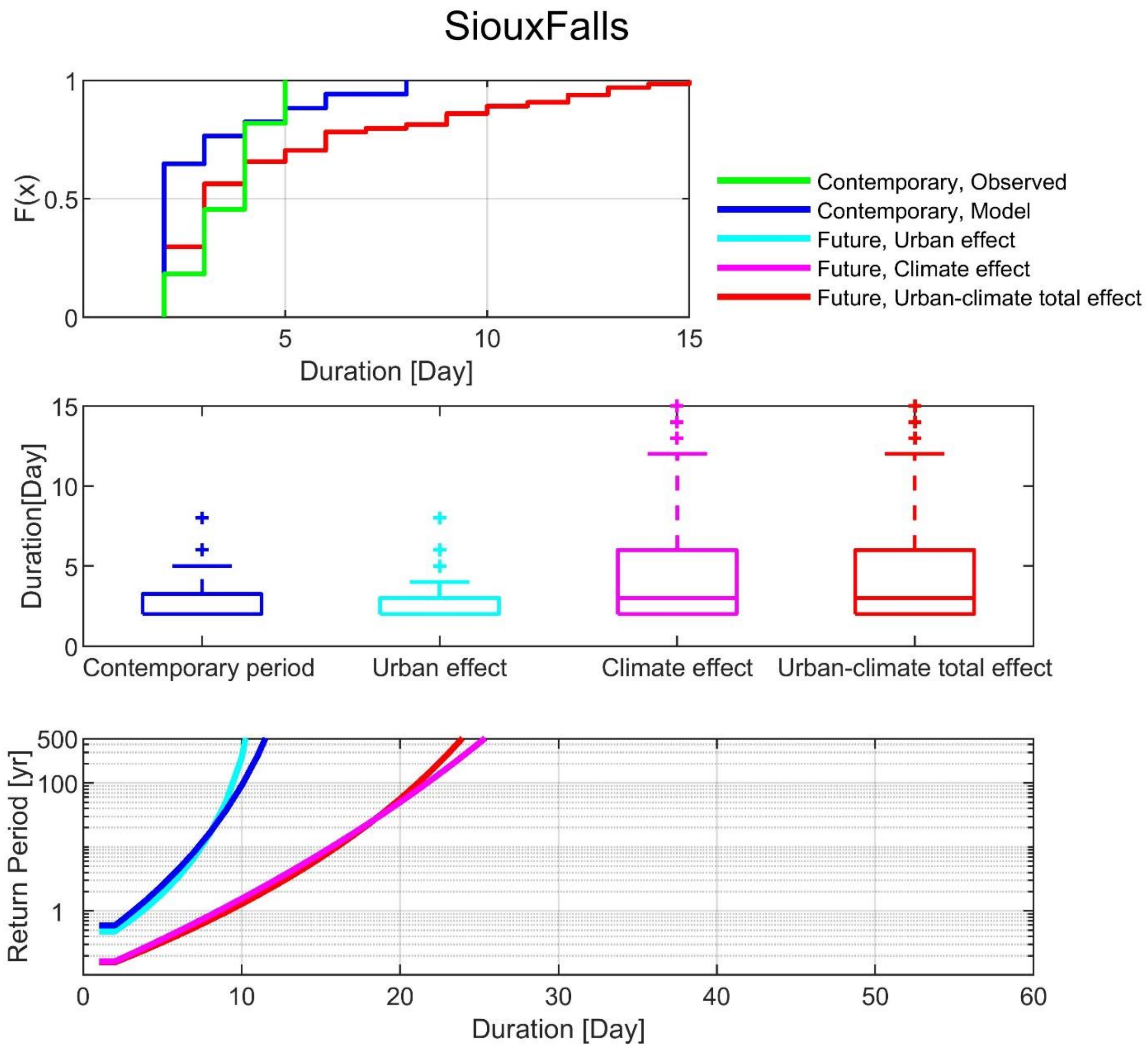

Figure S47. (Top panel) Cumulative distribution function (CDF) of compound dry-hot extreme (CDHE) events based on contemporary observed/model as well as future with consideration of the urban-climate total effect. (Middle panel) Boxplots of future CDHE events with consideration of the urban, climate, and urban-climate total effect. (Bottom panel) Return period return level plot for CDHE events for the contemporary period and future period with consideration of urban, climate, and urban-climate total effect.

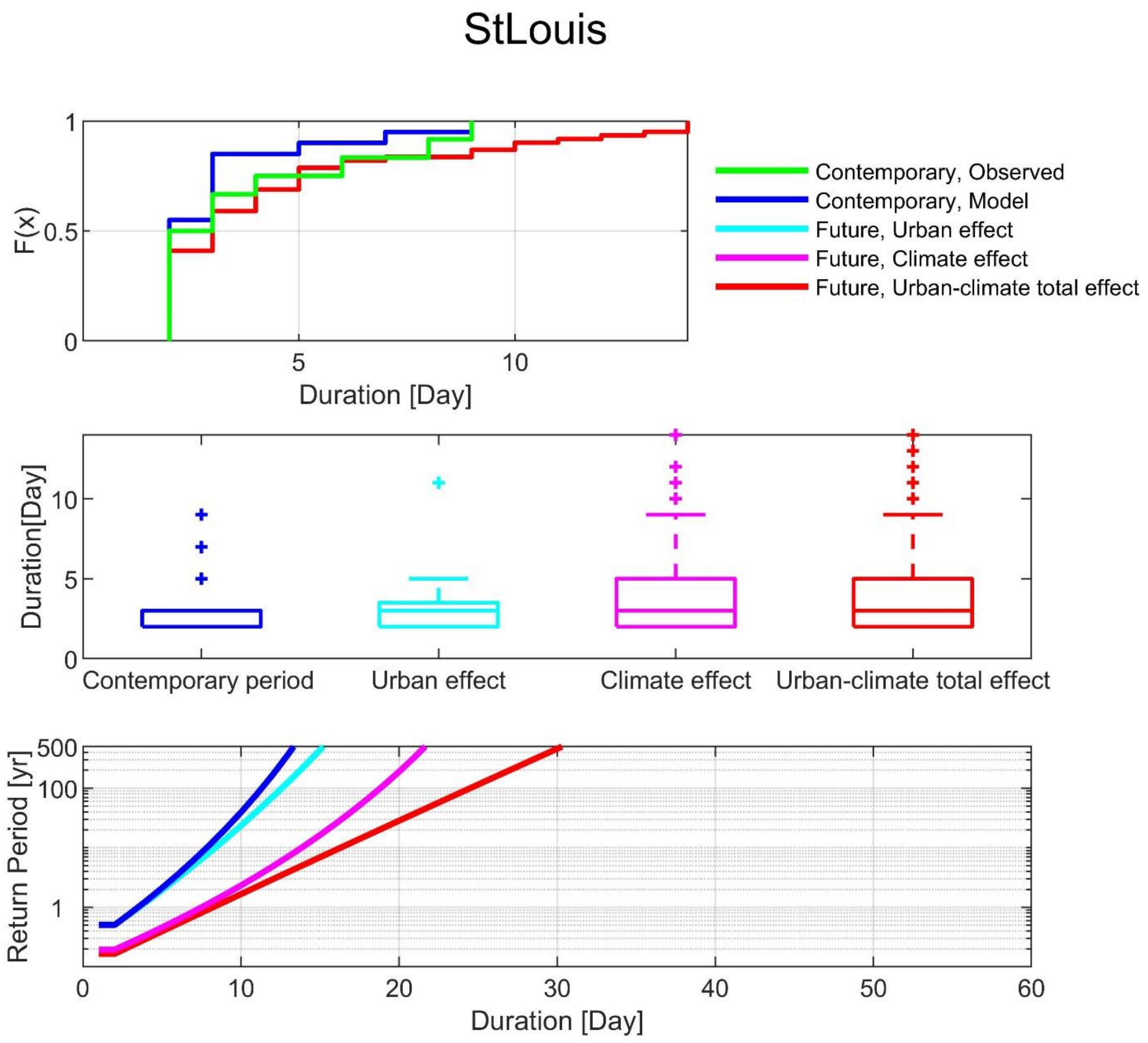

Figure S48. (Top panel) Cumulative distribution function (CDF) of compound dry-hot extreme (CDHE) events based on contemporary observed/model as well as future with consideration of the urban-climate total effect. (Middle panel) Boxplots of future CDHE events with consideration of the urban, climate, and urban-climate total effect. (Bottom panel) Return period return level plot for CDHE events for the contemporary period and future period with consideration of urban, climate, and urban-climate total effect.

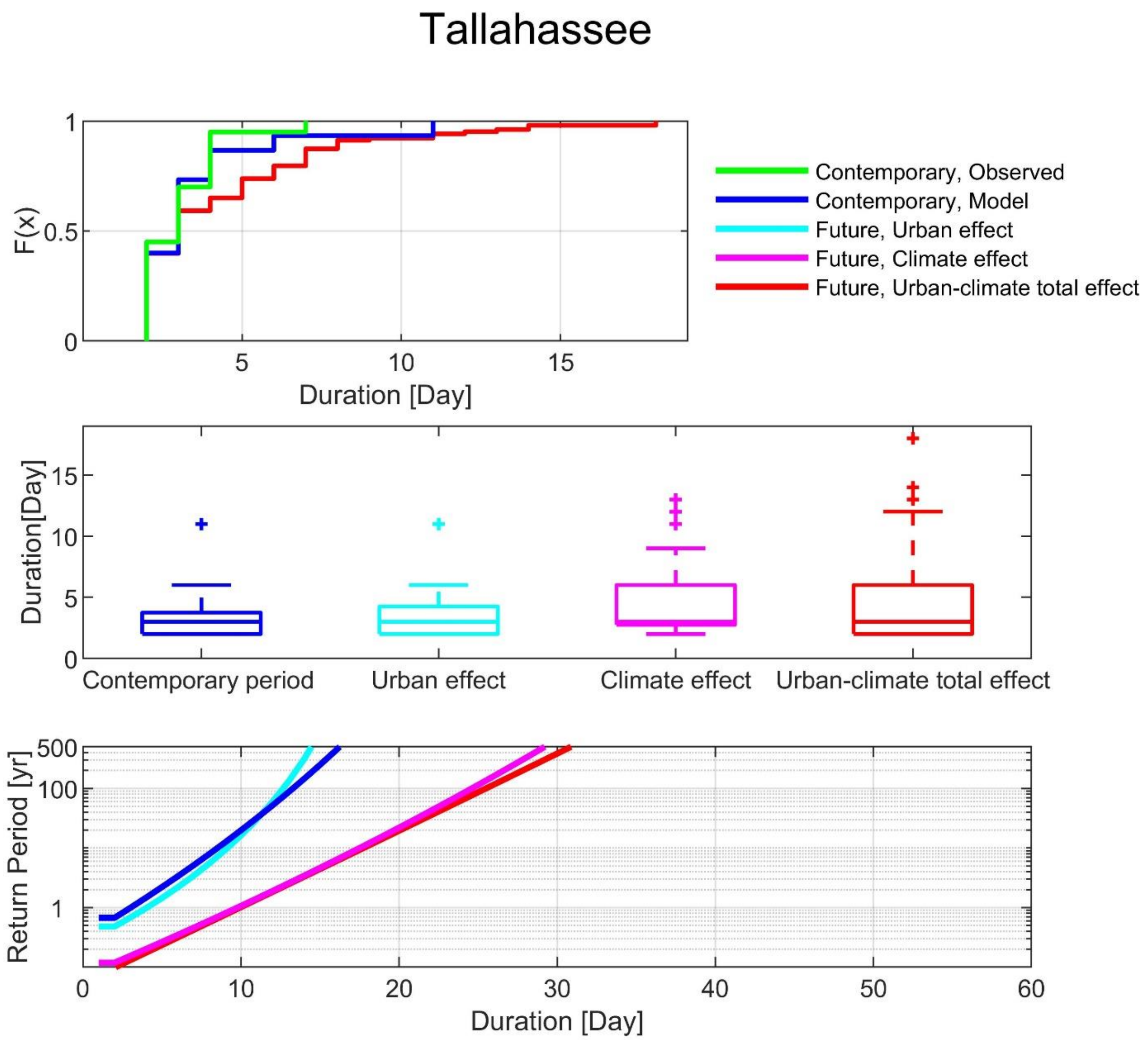

Figure S49. (Top panel) Cumulative distribution function (CDF) of compound dry-hot extreme (CDHE) events based on contemporary observed/model as well as future with consideration of the urban-climate total effect. (Middle panel) Boxplots of future CDHE events with consideration of the urban, climate, and urban-climate total effect. (Bottom panel) Return period return level plot for CDHE events for the contemporary period and future period with consideration of urban, climate, and urban-climate total effect.

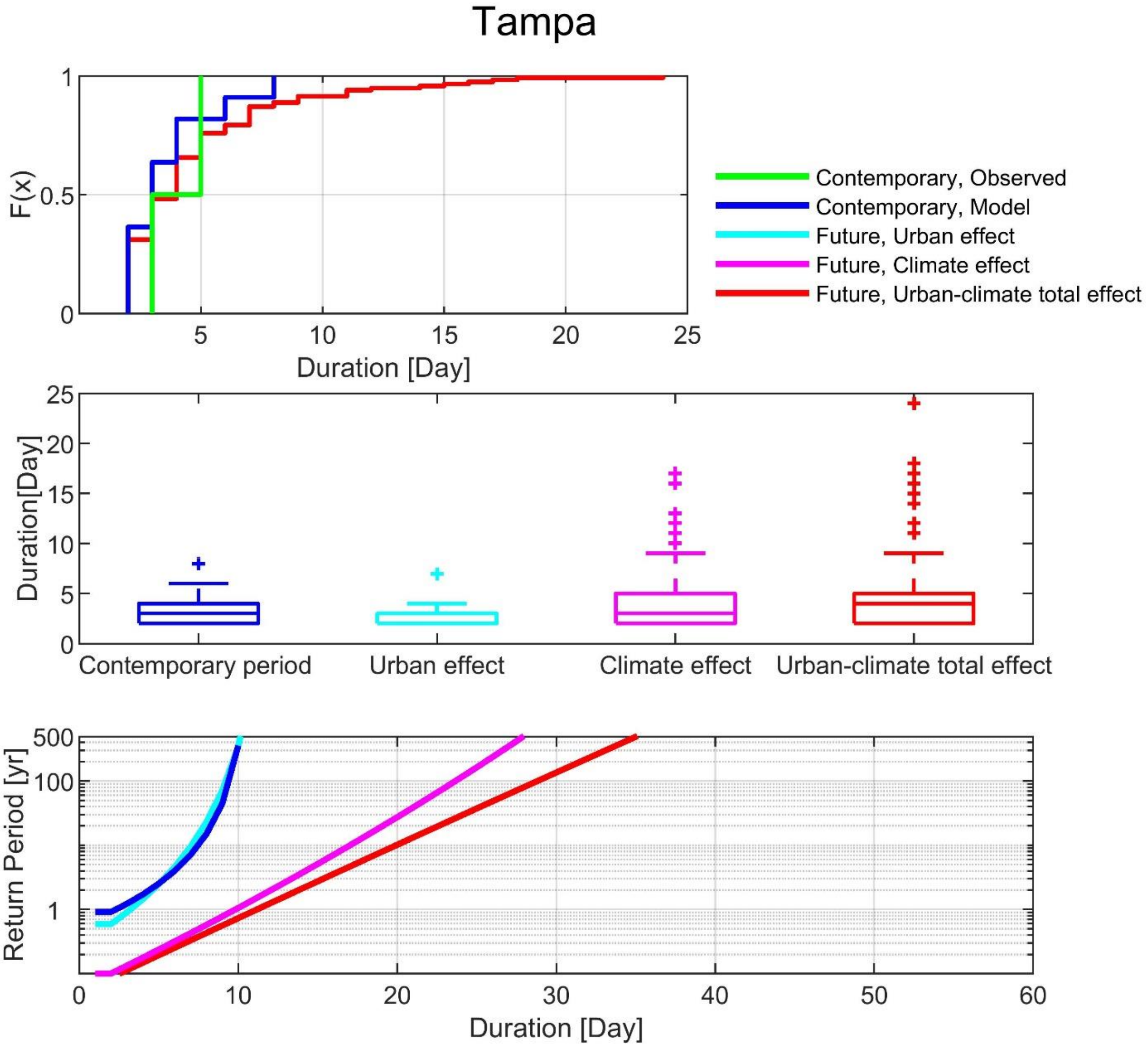

Figure S50. (Top panel) Cumulative distribution function (CDF) of compound dry-hot extreme (CDHE) events based on contemporary observed/model as well as future with consideration of the urban-climate total effect. (Middle panel) Boxplots of future CDHE events with consideration of the urban, climate, and urban-climate total effect. (Bottom panel) Return period return level plot for CDHE events for the contemporary period and future period with consideration of urban, climate, and urban-climate total effect.

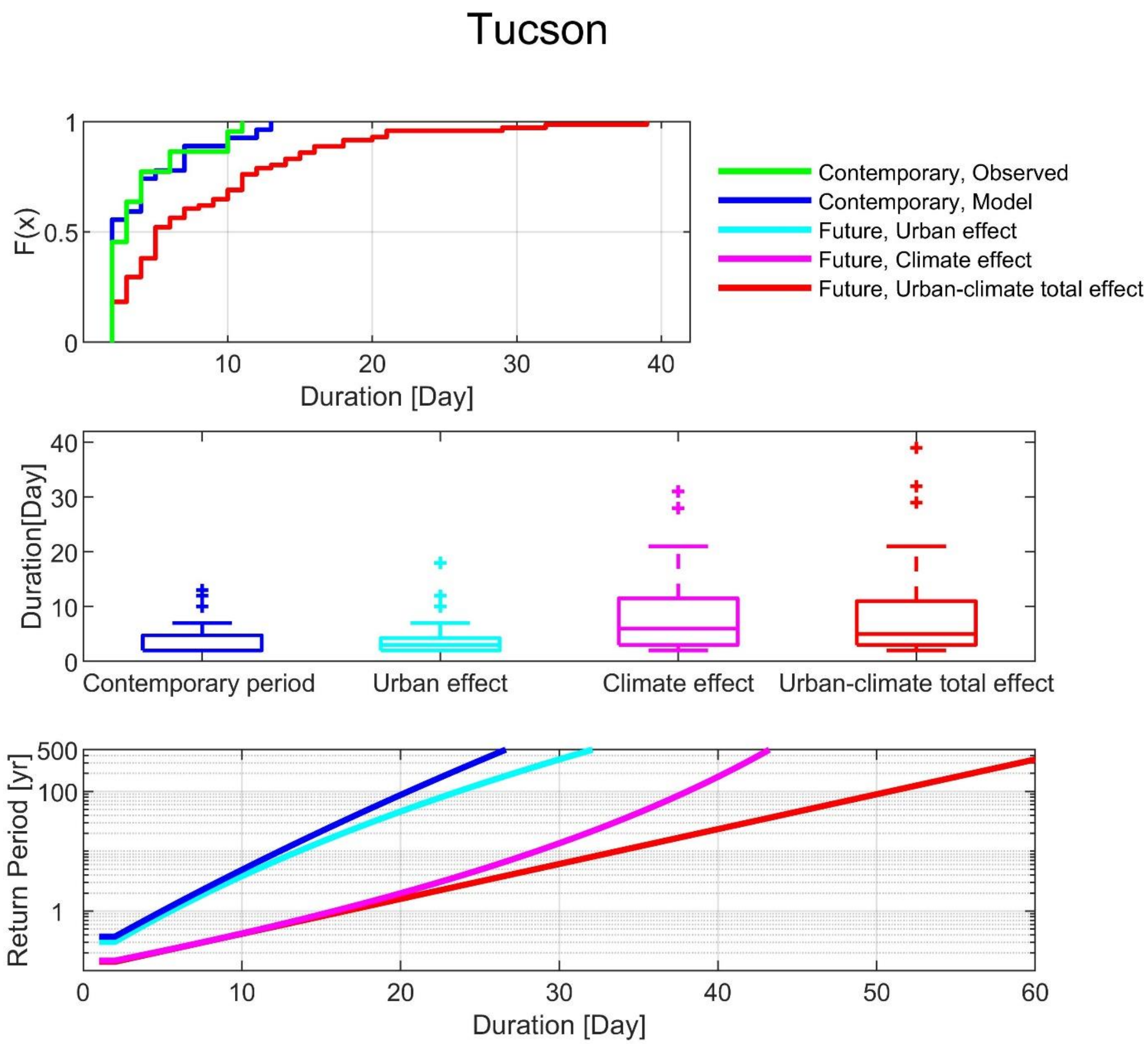

Figure S51. (Top panel) Cumulative distribution function (CDF) of compound dry-hot extreme (CDHE) events based on contemporary observed/model as well as future with consideration of the urban-climate total effect. (Middle panel) Boxplots of future CDHE events with consideration of the urban, climate, and urban-climate total effect. (Bottom panel) Return period return level plot for CDHE events for the contemporary period and future period with consideration of urban, climate, and urban-climate total effect.

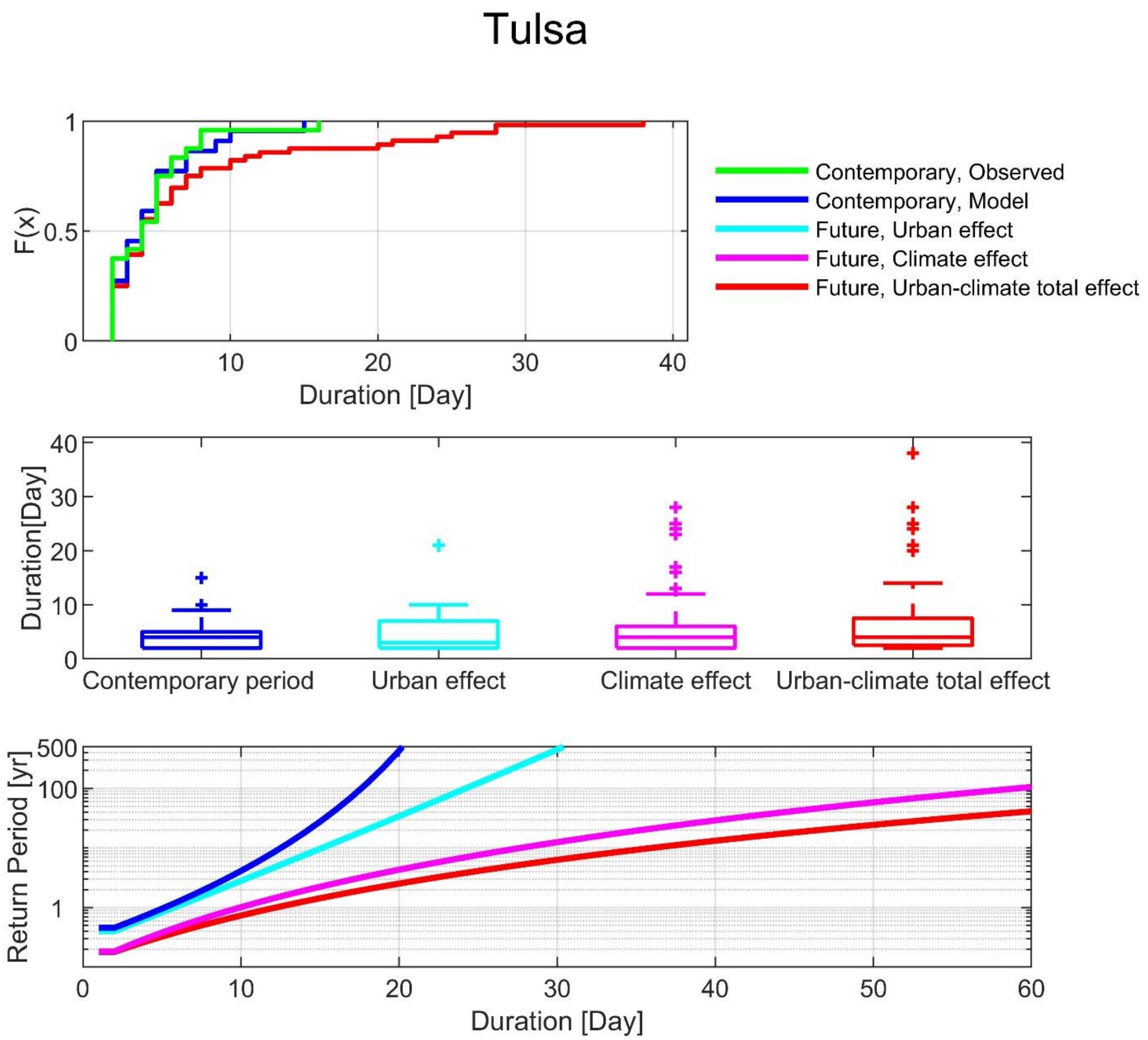

Figure S52. (Top panel) Cumulative distribution function (CDF) of compound dry-hot extreme (CDHE) events based on contemporary observed/model as well as future with consideration of the urban-climate total effect. (Middle panel) Boxplots of future CDHE events with consideration of the urban, climate, and urban-climate total effect. (Bottom panel) Return period return level plot for CDHE events for the contemporary period and future period with consideration of urban, climate, and urban-climate total effect.

Table S1. The name of major cities and corresponding weather station name

City	NOAA Station Name	Latitude	Langitude
Seattle	SEATTLE TACOMA AIRPORT, WA US	47.4444	-122.3138
Portland	PORTLAND INTERNATIONAL AIRPORT, OR US	45.5958	-122.6093
Minneapolis	MINNEAPOLIS ST. PAUL INTERNATIONAL AIRPORT, MN US	44.8831	-93.2289
Sioux Falls	SIOUX FALLS FOSS FIELD, SD US	43.5778	-96.7539
Boise	BOISE AIR TERMINAL, ID US	43.5666	-116.2406
Buffalo	BUFFALO NIAGARA INTERNATIONAL, NY US	42.9400	-78.7362
Boston	BOSTON, MA US	42.3606	-71.0097
Detroit	DETROIT METRO AIRPORT, MI US	42.2313	-83.3308
Chicago	CHICAGO OHARE INTERNATIONAL AIRPORT, IL US	41.9602	-87.9316
Cleveland	CLEVELAND HOPKINS INTERNATIONAL AIRPORT, OH US	41.4057	-81.8520
Omaha	OMAHA EPPLEY AIRFIELD, NE US	41.3102	-95.8991
Salt Lake City	SALT LAKE CITY INTERNATIONAL AIRPORT, UT US	40.7781	-111.9694
New York City	JFK INTERNATIONAL AIRPORT, NY US	40.6392	-73.7640
Pittsburgh	MCKEESPORT, PA US	40.3391	-79.8604
Columbus	COLUMBUS VLY CROSSING, OH US	39.9047	-82.9199
Philadelphia	PHILADELPHIA INTERNATIONAL AIRPORT, PA US	39.8733	-75.2268
Denver	DENVER INTERNATIONAL AIRPORT, CO US	39.8328	-104.6575
Indianapolis	INDIANAPOLIS INTERNATIONAL AIRPORT, IN US	39.7252	-86.2817
KansasCity	KANSAS CITY INTERNATIONAL AIRPORT, MO US	39.2972	-94.7306
Baltimore	MARYLAND SCIENCE CENTER, MD US	39.2814	-76.6111
Cincinnati	CINCINNATI NORTHERN KENTUCKY INTERNATIONAL AIRPORT, KY US	39.0444	-84.6724
StLouis	ST LOUIS SCIENCE CENTER, MO US	38.6308	-90.2708
Sacramento	SACRAMENTO AIRPORT ASOS, CA US	38.5069	-121.4950
Louisville	LOUISVILLE INTERNATIONAL AIRPORT, KY US	38.1811	-85.7391
Richmond	RICHMOND INTERNATIONAL AIRPORT, VA US	37.5115	-77.3234
Norfolk	NORFOLK INTERNATIONAL AIRPORT, VA US	36.9033	-76.1922
Fresno	FRESNO YOSEMITE INTERNATIONAL, CA US	36.7800	-119.7194
Nashville	NASHVILLE INTERNATIONAL AIRPORT, TN US	36.1189	-86.6892
LasVegas	MCCARRAN INTERNATIONAL AIRPORT, NV US	36.0719	-115.1634
Tulsa	TULSA RICHARD L JONES JR AIRPORT, OK US	36.0394	-95.9844
Raleigh	RALEIGH AIRPORT, NC US	35.8923	-78.7819
Oklahoma City	OKLAHOMA CITY WILL ROGERS WORLD AIRPORT, OK US	35.3889	-97.6006
Charlotte	CHARLOTTE DOUGLAS AIRPORT, NC US	35.2236	-80.9552
Albuquerque	ALBUQUERQUE FOOTHILLS NE, NM US	35.1316	-106.4952
Memphis	MEMPHIS INTERNATIONAL AIRPORT, TN US	35.0564	-89.9865
LosAngeles	LOS ANGELES DOWNTOWN USC, CA US	34.0236	-118.2911
Atlanta	ATLANTA DEKALB PEACHTREE AIRPORT, GA US	33.8750	-84.3022
Phoenix	PHOENIX AIRPORT, AZ US	33.4277	-112.0038
Dallas	DALLAS FAA AIRPORT, TX US	32.8519	-96.8555
SanDiego	SAN DIEGO INTERNATIONAL AIRPORT, CA US	32.7336	-117.1831
Tucson	TUCSON INTERNATIONAL AIRPORT, AZ US	32.1313	-110.9552
Tallahassee	TALLAHASSEE REGIONAL AIRPORT, FL US	30.3931	-84.3533
Austin	AUSTIN CAMP MABRY, TX US	30.3208	-97.7604
Jacksonville	JACKSONVILLE NAS, FL US	30.2333	-81.6667
New Orleans	NEW ORLEANS AIRPORT, LA US	29.9969	-90.2775
Houston	HOUSTON SUGARLAND MEM, TX US	29.6219	-95.6567
SanAntonio	SAN ANTONIO INTERNATIONAL AIRPORT, TX US	29.5443	-98.4839
Orlando	ORLANDO INTERNATIONAL AIRPORT, FL US	28.4339	-81.3250
Tampa	TAMPA INTERNATIONAL AIRPORT, FL US	27.9619	-82.5403
Miami	MIAMI INTERNATIONAL AIRPORT, FL US	25.7881	-80.3169
